# Bis(2-butoxyethyl) Ether-Promoted O_2_-Mediated Oxidation of Alkyl Aromatics to Ketones under Clean Conditions

**DOI:** 10.3390/molecules29204909

**Published:** 2024-10-17

**Authors:** Yangyang Xie, Zeping Li, Xudong Xu, Han Jiang, Keyi Chen, Jinhua Ou, Kaijian Liu, Yihui Zhou, Kejun Luo

**Affiliations:** 1 Department of Material and Chemical Engineering, Hunan Institute of Technology, Hengyang 421002, China; 19189513063@163.com (Y.X.); 18960398285@163.com (Z.L.); 15377337985@163.com (X.X.); 15773411989@163.com (H.J.); 19577219091@163.com (K.C.); 2 Collaborative Innovation Center, Hunan Automotive Engineering Vocational College, Zhuzhou 412001, China; 3 College of Chemistry and Chemical Engineering, Hunan University, Changsha 410082, China; 4 Changsha Research Institute of Mining and Metallurgy Co., Ltd., Changsha 410012, China

**Keywords:** oxidation, alkyl aromatics, ketones, bis(2-butoxyethyl) ether, metal-free

## Abstract

Conventional oxidation processes for alkyl aromatics to ketones employ oxidants that tend to generate harmful byproducts and cause severe equipment corrosion, ultimately creating critical environmental problems. Thus, in this study, a practical, efficient, and green method was developed for the synthesis of aromatic ketones by applying a bis(2-butoxyethyl) ether/O_2_ system under external catalyst-, additive-, and base-free conditions. This O_2_-mediated oxidation system can tolerate various functional groups and is suitable for large-scale synthesis. Diverse target ketones were prepared under clean conditions in moderate-to-high yields. The late-stage functionalization of drug derivatives with the corresponding ketones and one-pot sequential chemical conversions to ketone downstream products further broaden the application prospects of this approach.

## 1. Introduction

The selective oxidation of hydrocarbons to aromatic ketones is an important organic conversion process in both the laboratory and fine chemicals industry [1,2,3]. Compared with the selective oxidation of styrene [4,5,6], 1-phenyl ethanol [7,8,9], isopropyl benzene [10], etc., the oxygenation of alkyl aromatics is a more popular pathway for preparing ketones because of its low cost and easy availability [11,12,13]. In conventional oxidation processes for alkyl aromatics to ketones, stoichiometric oxidants such as Oxone [14], K_2_S_2_O_8_ [15], [PhIO]_n_/KBr [16], IBX [17], and *m*-CPBA [18] are typically adopted, but these oxidants tend to generate harmful byproducts and cause severe equipment corrosion, ultimately leading to critical environmental problems. Molecular oxygen is considered to be an attractive oxidant and O-atom source for the synthesis of O-containing compounds because it is environmentally friendly, abundant, and inexpensive [19,20,21,22,23]. In recent years, excellent C(sp^3^)–H bond oxygenation of aromatics to ketones has been achieved under an atmosphere of air or dioxygen by using metal salt [24,25,26], metal–organic complexes [27,28,29], metal-supported nanoparticles [30,31,32], zeolite [33,34], and carbon [35] as catalysts.

**Scheme 1:**
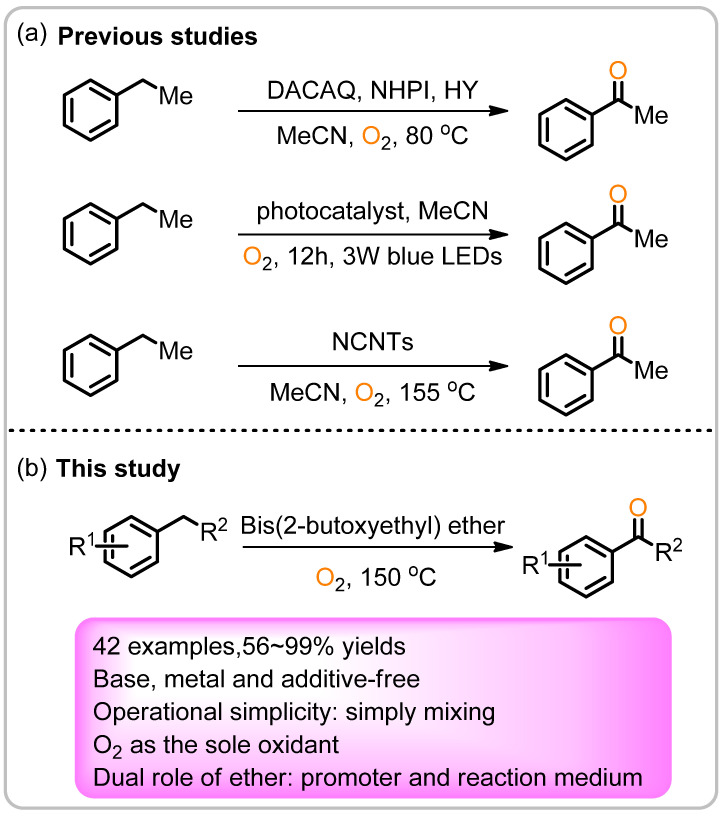
Oxidation of alkyl aromatics to ketones.

Compared to metal-based catalyzed reactions, metal-free oxidation is more practical in the production of agrochemical and pharmaceuticals [36,37,38], as it does not require the removal of metal residues and reduces the cost of wastewater treatment. In 2004, Xu et al. developed a three-component biomimetic catalytic system for the ketonization of hydrocarbons driven via electron transfer by using N-hydroxyphthalimide (NHPI) as a nonmetallic redox center and O_2_ as the ultimate oxidant [39]. In 2015, Yi et al. reported on Acr^+^-Mes ClO_4_^−^-promoted benzylic sp^3^ C–H oxidation by visible light, whereby Acr^+^-Mes ClO_4_^−^ radical anions were oxidized by O_2_ to ensure the regeneration of a photocatalyst; their findings offered a new perspective on obtaining various kinds of ketones under relatively mild conditions [40]. In 2021, Shi and colleagues applied N-doped carbon nanotubes and TBHP/O_2_ as the catalyst and oxidants, respectively, for ethylbenzene oxidation; they found that the addition of TBHP promoted α-H abstraction and radical propagation, thereby enabling a considerable degree of conversion [41]. Recently, Zhang et al. prepared an NHPI-functionalized activated carbon catalyst system for the aerobic oxidation of ethylbenzene and successfully enhanced the space–time yield of acetophenone [42] (Scheme 1a). Although metal-free catalytic systems have demonstrated significant potential for application in the process of oxygenating benzylic C-H bonds to convert them into the corresponding ketones, they all require an additional catalyst to activate O_2_ and achieve a sufficient level of conversion. Furthermore, they have limitations such as high reagent costs, poor thermal stability, overoxidized byproducts, and being difficult to separate. Additionally, their limited substrate generalizability, low atom efficiency, and poor reusability limit their industrial application.

Thus, researchers have channeled their efforts toward exploring novel green, highly efficient, and insignificant capital investment oxidation approaches for alkyl aromatics that are suitable for industrial needs [43,44,45]. However, there are no reports on the ether-promoted oxygenation of ketones from alkyl aromatics in the absence of an external catalyst or an additive. As an extension of our interest in green chemistry and O_2_-mediated oxygenation reactions [46,47,48,49,50], we present a practical, efficient, and green method for the synthesis of aromatic ketones in the presence of O_2_ that entails applying bis(2-butoxyethyl) ether as a promoter and solvent under external catalyst-, additive-, and base-free conditions (Scheme 1b).

## 2. Results and Discussion

The primary aim of this study was to identify a suitable promoter for the conversion of 4-ethyltoluene **1a** into the corresponding 4-acetyltoluene **2a** (Table 1). Firstly, we optimized the reaction conditions with 4-ethyltoluene as raw material and ether as promoter in the presence of O_2_ at 120 °C. Several common solvents were investigated in the presence of O_2,_ including 2-methoxyethyl ether, dimethoxydipropylene glycol, triethylene glycol, and dimethyl ether (Entries 1–8 in Table 1). Among these solvents, bis(2-butoxyethyl) ether, as a promoter, exhibited the highest catalytic efficiency, achieving a 70% yield. Methoxypropoxypropanol resulted in the lowest yield (31%), possibly because of the quenching of free radicals by the hydroxyl groups (Entry 8). The temperature optimization tests confirmed that 150 °C was an appropriate temperature in this oxidation reaction (Entries 9–14). It was also notable that, after the optimal promoter load was determined (Entries 15–17), 4-acetyltoluene **2a** was obtained in 97% GC-MS yield under an O_2_ atmosphere at 150 °C for 15 h (Entry 16). Extending the reaction time or conducting the experiment in a dark room minimally influenced oxidation (Entries 18 and 19). In contrast, oxidation either did not occur or was significantly constrained when dioxygen was replaced with N_2_ and air, respectively (Entries 20 and 21). This shows that molecular oxygen plays a key role in oxidation, and that the oxygen concentration significantly affects the system. No target product was obtained when the system was implemented without bis(2-butoxyethyl) ether, which shows that bis(2-butoxyethyl) ether promotes the oxidation reaction (Entry 22).

Subsequently, the scope and limitations of alkyl aromatics that exhibit a variety of substitution patterns were explored under the optimal conditions; the results are illustrated in Table 2. Ethyl aromatics with electron-withdrawing or electron-donating groups were successfully transformed into the desired ketones with good to excellent yield (**2a**–**2m**). It is noteworthy that substituent groups with an electron-withdrawing nature reduced the reaction efficiency of the substrate; this may be ascribed to the reduced electron cloud density in the benzene ring, which impeded benzyl radical formation. Additionally, compared with the para- and meta-substituted substrates (**2a** and **2l**), the use of the ortho-substituted ethylbenzene with strong steric hindrance effects also resulted in a relatively low yield (**2m**). Although the oxidation strategy of accessing di-oxygenated products by using di-alkyl aromatics as substrates is often ineffective because the electron-withdrawing effect of the carbonyl groups on the mono-ketones reduces the reactivity, our reaction proceeded well with di-ethyl benzene under standard conditions; particularly, we obtained the double-oxidation product (**2n**) at 78% yield. Poly-substituted ethylbenzenes were also evaluated, and the target ketones were obtained in excellent yields (**2o** and **2p**). Moreover, various aromatic hydrocarbons with (hetero)cyclic rings containing methylene were perfectly tolerated under the standard conditions, resulting in yields of 74–95% for the corresponding ketones (**2q**–**2t**). Notably, heterocyclic and polycyclic alkylbenzenes also yielded the desired product (**2u**–**2y**), but the presence of electron-withdrawing nitrogen atoms in the aromatic ring resulted in slightly lower yields of 4-acetylpyridine (**2w**), further confirming the influence of the electronic effect on the reaction. Similarly, a variety of β-substituted alkylbenzenes were also found to be compatible with the bis(2-butoxyethyl) ether-promoted oxidation reaction, and a moderate yield (61–78%) of product was obtained because of steric hindrance (**2z**–**2ab**). Moreover, substituted diarylmethane with H, Me, or Cl groups were readily converted, generating corresponding ketone yields within the range of 84–92% (**2ac**–**2af**). Unfortunately, no target product was observed with the less active 2-ethyl or 2-benzylpyridine as the substrate. It is also noteworthy that, when 9,10-dihydroanthracene was applied in this reaction, anthracene was obtained as the main product at 78% yield (**4a**); this is likely because the fully aromatized product was more stable. When benzyl alcohol was used as substrate, the deep oxidation product benzoic acid (**2ah**) was obtained with almost quantitative yield, which further expanded the adaptive range of the system.

In consideration of the greenness and practicability of the oxidation approach and the importance of late-stage functionalization of natural products for drug research, several drug derivatives were subjected to this system under optimal reaction conditions. Notably, the anti-inflammatory indomethacin (**2ah**); cholesterol (**2ai**), which sources cholic acid and hormones in the body; dehydroandrosterone (**2aj**), which is used for anti-aging and protein assimilation; and the lipid-lowering drug fenofibrate (**2ak**) were all successfully converted into the corresponding ketones with yields ranging from 69 to 77%. These findings indicate the wide-ranging applicability of the proposed oxidation system in the pharmaceutical industry.

**Figure 1:**
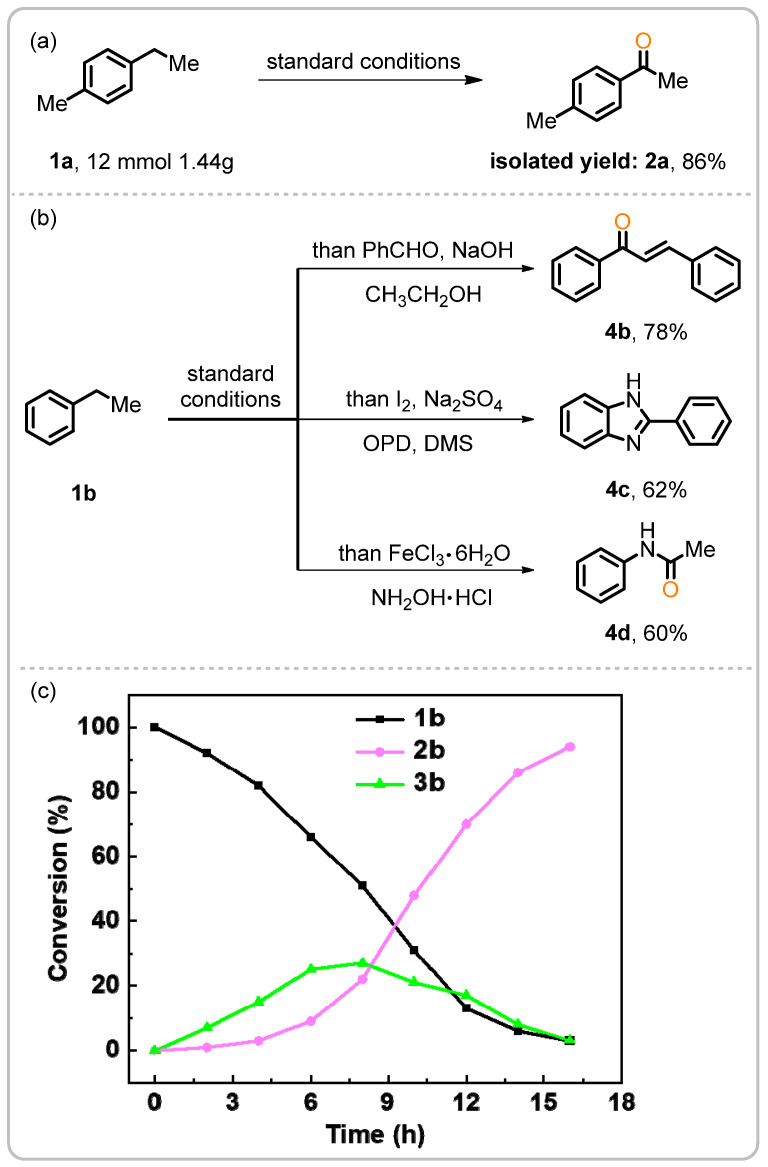
(**a**) Scaled-up oxidation reaction; (**b**) several one-pot sequential conversions; (**c**) time course of oxygenation.

We also attempted a scaled-up oxidation reaction with 4-ethyltoluene **1a** as the substrate under standard conditions; we obtained a 4-acetyltoluene **2a** yield of 86%, further revealing the superiority of the proposed green oxidation method (Figure 1a). To prove the operating convenience of the proposed oxidation system, we confirmed that several one-pot sequential chemical conversions, i.e., aldol condensation (**1b** → **4b**) [51], cyclization (**1b** → **4c**) [52], and amidation (**1b** → **4d**) [53], could be generated from a crude reaction mixture (Figure 1b). The desired ketone downstream products were smoothly isolated in overall yields ranging from 60 to 78%, demonstrating the efficiency and cleanliness of this method for subsequent direct transformations. The conversion of ethylbenzene **1b** over time was monitored by applying GC-MS under the optimal conditions; the relative results are displayed in Figure 1c. In the first 6 h, 9% acetophenone **2b** and 25% 1-phenylethanol **3b** yields were observed, in addition to a 34% consumption of the starting substrate **1b**, indicating that the transformation of **1b** into **3b** was the primary reaction in the initial phase. Subsequently, the target product **2b** rapidly increased until ethylbenzene **1b** was exhausted, whereas 1-phenylethanol **3b** gradually disappeared over time at a later stage. These trends may be attributable to the fact that the oxidation of **1b** was first converted into alcohol **3b** and then further oxidized to the target ketone **2b** [36,40,42].

**Figure 2:**
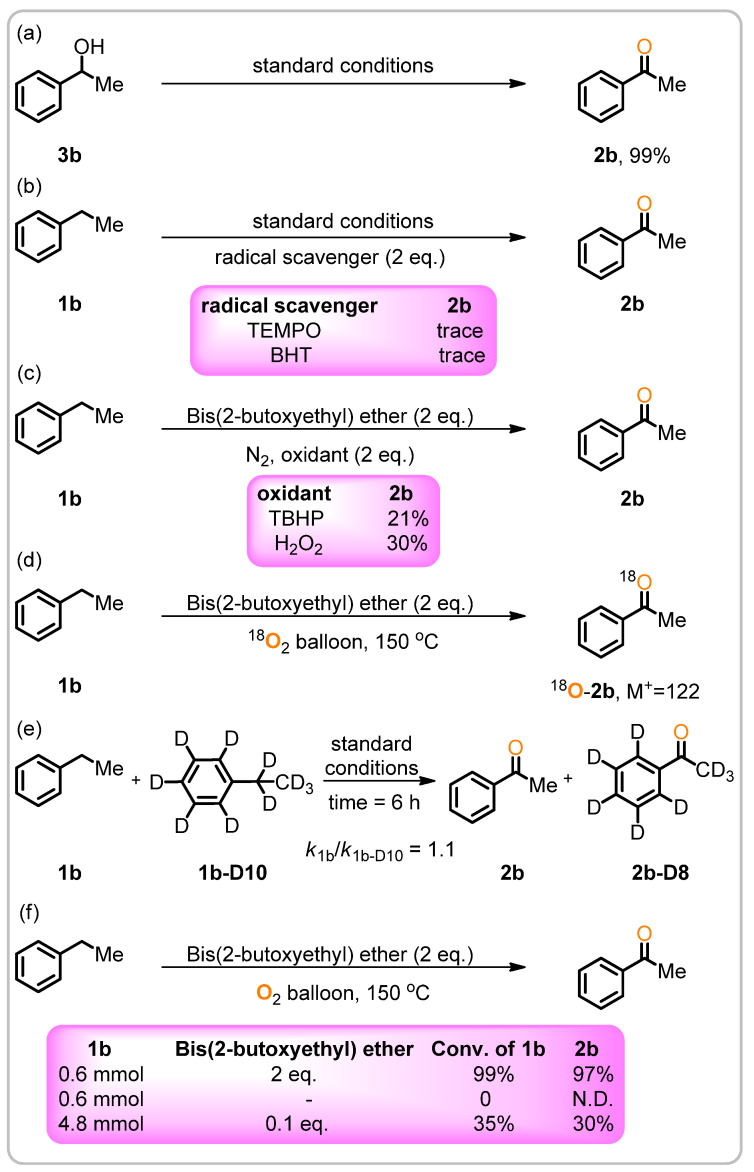
Control experiments.

The aforementioned results prompted us to gain insight into the reaction mechanism. Thus, several experiments were performed, as shown in Figure 2. In the first experiment, we induced the oxidation reaction by applying 1-phenylethanol **3b** as the substrate under the standard reaction conditions, consequently obtaining acetophenone **2b** in 99% yield (Figure 2a). This result, combined with the fact that 1-phenylethanol **3b** was present throughout the experimental monitoring period (Figure 1c), indicates that **3b** may be involved in oxygenation as a critical intermediate. The oxidation reaction was largely ineffective in the presence of a trapping agent, i.e., TEMPO or BHT (Figure 2b), suggesting that the system may involve a free radical mechanism [24,39,40]. In a subsequent oxidation reaction experiment, we replaced dioxygen with TBHP or H_2_O_2_ as an oxidizing agent in a N_2_ atmosphere and only obtained a 21–30% acetophenone **2b** yield (Figure 2c), further confirming the critical role of O_2_ in this oxidation approach.

When ^18^O_2_ was used as the sole oxidant under the optimized conditions, only ^18^O-labeled **2b** was detected (Figure 2d), revealing that the O atom of the carbonyl group was derived from molecular oxygen rather than bis(2-butoxyethyl) ether. We subsequently performed a kinetic isotope effect experiment by applying the same molar amount of ethylbenzene **1b** and ethylbenzene-D10 (**1b**–**D10**) to produce a mixed substrate (Figure 2e). The clear *k_1b_*/*k_1b-D10_* value of 1.1 obtained for this oxidation system indicates that the cleavage of the methylene C(sp^3^)–H bond may not be the rate-determining step. To further clarify the crucial role of bis(2-butoxyethyl) ether, we evaluated the promoter concentration (Figure 2f). No target compounds were observed when the reaction was performed in the absence of bis(2-butoxyethyl) ether. Reducing the concentration of the promoter also negatively impacted the oxidation process, as 0.1 eq. of bis(2-butoxyethyl) ether only resulted in a 30% yield of acetyltoluene **2b**. We can infer from this phenomenon that bis(2-butoxyethyl) ether promotes the oxidation reaction as both a catalyst and reaction medium.

**Figure 3:**
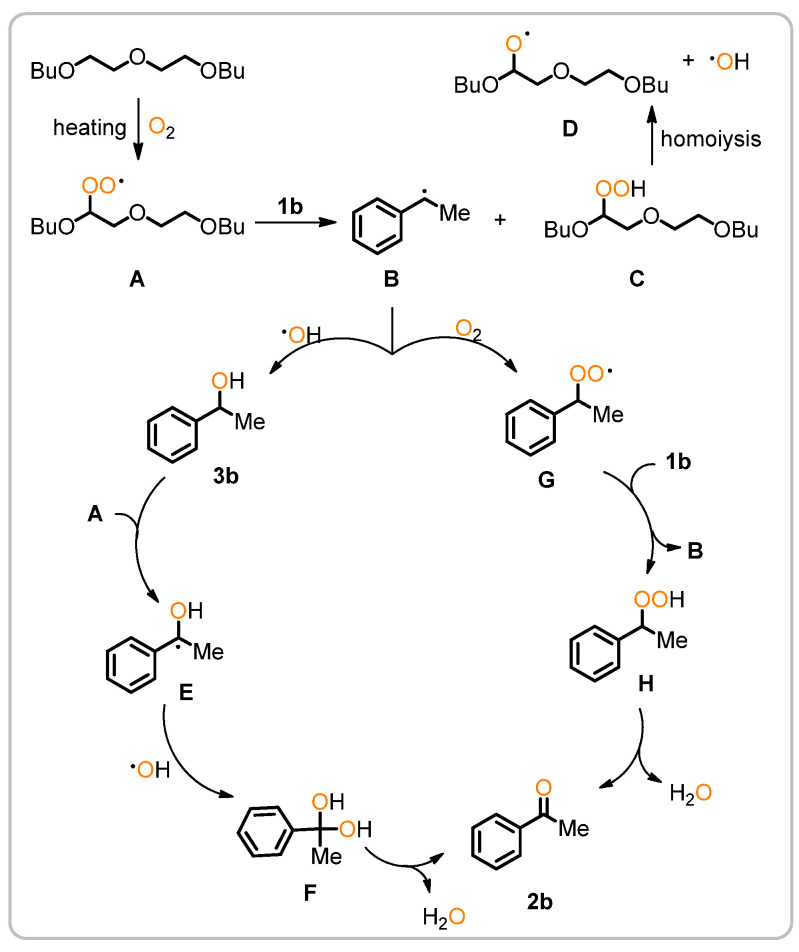
Plausible bis(2-butoxyethyl) ether promoted oxidation mechanism.

Based on the above-mentioned results and related reports [54,55,56,57], a possible bis(2-butoxyethyl) ether-promoted oxidation mechanism was constructed, as shown in Figure 3. First, the bis(2-butoxyethyl) ether reacted with O_2_ under heating conditions to form the peroxyl radical **A**, which seized an α-H atom from ethylbenzene **1b** to produce the benzyl radical intermediate **B** and peroxide **C**. Then, the peroxide **C** was converted into the oxygen radical **D** and hydroxyl radical by homolysis. Then, the hydroxyl radical induced the production of 1-phenylethanol **3b** through the benzyl radical **B** (detected via GC-MS), which was captured an α-H atom by peroxyl radical **A** and further transformed into the carbon-center radical **E**. The intermediate **E** was found to readily join with hydroxyl radicals to form the diol intermediate **F**, which is unstable and dehydrates to produce the ketone **2b**. Additionally, the benzyl radical **B** was found to be able to act with molecular oxygen to produce the peroxide radical **G**, which seized an α-H atom of ethylbenzene **1b** to form the peroxide molecule **H** with concomitant release of benzyl radical **B**. Lastly, the β-H cleavage of the peroxide molecule **H** and elimination of H_2_O resulted in the formation of the ketone **2b**.

## 3. Experimental Section

### 3.1. General Procedure for the Synthesis of Ketones 2

A mixture of alkyl benzene **1** (0.6 mmol) and bis(2-butoxyethyl) ether (1.2 mmol) was added to a 15 mL glass tube with an O2 balloon at room temperature. Then the contents were stirred at 150 °C for 15 h. The reaction mixture was purified by silica gel column chromatography using a mixture of petroleum ether and ethyl acetate as the eluent to afford the desired **2** (Sections S1 and S2).

### 3.2. Characterization Data of Products **2a**–**2ak** and **4a**–**4d**

*1-(p-tolyl)ethan-1-one* (**2a**) [56]: Colorless liquid (74 mg, 92%); ^1^H NMR (400 MHz, CDCl_3_) δ 7.93 (d, *J* = 8.0 Hz, 2H), 7.33 (d, *J* = 8.0 Hz, 2H), 2.65 (s, 3H), 2.48 (s, 3H); ^13^C NMR (100 MHz, CDCl_3_) δ 197.7, 143.8, 134.6, 129.1, 128.3, 26.4, 21.5.

*Acetophenone* (**2b**) [56]: Colorless liquid (65 mg, 90%); ^1^H NMR (400 MHz, CDCl_3_) δ 8.03 (d, *J* = 8.0 Hz, 2H), 7.63 (t, *J* = 7.6 Hz, 1H), 7.53 (t, *J* = 8.0 Hz, 2H), 2.67 (s, 3H); ^13^C NMR (100 MHz, CDCl_3_) δ 198.1, 137.0, 133.0, 128.5, 128.2, 26.5.

*1-(4-(tert-butyl)phenyl)ethan-1-one* (**2c**) [58]: Colorless liquid (98 mg, 93%); ^1^H NMR (400 MHz, CDCl_3_) δ 7.99 (d, *J* = 8.4 Hz, 2H), 7.47 (d, *J* = 8.0 Hz, 2H), 2.57 (s, 3H), 1.33 (s, 9H); ^13^C NMR (100 MHz, CDCl_3_) δ 197.7, 156.7, 134.5, 128.2, 125.4, 35.0, 31.0, 26.4.

*1-(4-methoxyphenyl)ethan-1-one* (**2d**) [56]: White solid (80 mg, 89%); ^1^H NMR (400 MHz, CDCl_3_) δ 7.88 (d, *J* = 8.8 Hz, 2H), 6.87 (d, *J* = 8.8 Hz, 2H), 3.80 (s, 3H), 2.49 (s, 9H); ^13^C NMR (100 MHz, CDCl_3_) δ 196.5, 163.3, 130.4, 130.1, 113.5, 55.2, 26.1.

*1-(4-fluorophenyl)ethan-1-one* (**2e**) [56]: Colorless liquid (72 mg, 87%); ^1^H NMR (400 MHz, CDCl_3_) δ 7.95 (dd, *J* = 8.80, 5.6 Hz, 2H), 7.09 (t, *J* = 8.4 Hz, 2H), 2.55 (s, 9H); ^13^C NMR (100 MHz, CDCl_3_) δ 196.4, 165.6 (d, *J* = 255.6 Hz), 133.5 (d, *J* = 3.0 Hz), 130.9 (d, *J* = 9.4 Hz), 115.5 (d, *J* = 21.9 Hz), 26.4; ^19^F NMR (376 MHz, CD_3_Cl) δ −105.4.

*1-(4-chlorophenyl)ethan-1-one* (**2f**) [56]: Colorless liquid (84 mg, 91%); ^1^H NMR (400 MHz, CDCl_3_) δ 7.85 (d, *J* = 8.4 Hz, 2H), 7.38 (d, *J* = 8.4 Hz, 2H), 2.55 (s, 3H); ^13^C NMR (100 MHz, CDCl_3_) δ 196.7, 139.4, 135.3, 129.6, 128.7, 26.4.

*1-(4-bromophenyl)ethan-1-one* (**2g**) [56]: White solid (95 mg, 80%); ^1^H NMR (400 MHz, CDCl_3_) δ 7.82 (d, *J* = 8.4 Hz, 2H), 7.60 (d, *J* = 8.4 Hz, 2H), 2.58 (s, 3H); ^13^C NMR (100 MHz, CDCl_3_) δ 197.0, 135.8, 131.9, 129.8, 128.3, 26.5.

*1-(4-iodophenyl)ethan-1-one* (**2h**) [56]: Brown solid (115 mg, 78%); ^1^H NMR (400 MHz, CDCl_3_) δ 7.82 (d, *J* = 8.0 Hz, 2H), 7.65 (d, *J* = 8.4 Hz, 2H), 2.56 (s, 3H); ^13^C NMR (100 MHz, CDCl_3_) δ 197.3, 137.9, 136.3, 129.7, 101.1, 26.4.

*1-(4-(trifluoromethyl)phenyl)ethan-1-one* (**2i**) [56]: Colorless liquid (81 mg, 72%); ^1^H NMR (400 MHz, CDCl_3_) δ 8.04 (d, *J* = 8.4 Hz, 2H), 7.71 (d, *J* = 8.0 Hz, 2H), 2.63 (s, 3H); ^13^C NMR (100 MHz, CDCl_3_) δ 196.9, 139.6, 134.4 (q, *J* = 32.8 Hz), 128.6, 125.6 (q, *J* = 63.3 Hz), 123.6 (q, *J* = 273.7 Hz), 26.7; ^19^F NMR (376 MHz, CD_3_Cl) δ −63.2.

*4-acetylbenzonitrile* (**2j**) [59]: Yellowish solid (60 mg, 69%); ^1^H NMR (400 MHz, CDCl_3_) δ 8.04 (d, *J* = 8.0 Hz, 2H), 7.77 (d, *J* = 8.4 Hz, 2H), 2.64 (s, 3H); ^13^C NMR (100 MHz, CDCl_3_) δ 196.5, 139.9, 132.5, 128.7, 117.9, 116.4, 26.7.

*methyl 4-acetylbenzoate* (**2k**) [59]: White solid (87 mg, 82%); ^1^H NMR (400 MHz, CDCl_3_) δ 8.13 (d, *J* = 8.4 Hz, 2H), 8.01 (d, *J* = 8.4 Hz, 2H), 3.95 (s, 3H), 2.65 (s, 3H); ^13^C NMR (100 MHz, CDCl_3_) δ 197.6, 166.2, 140.2, 133.9, 129.8, 128.2, 52.5, 26.9.

*1-(m-tolyl)ethan-1-one* (**2l**) [56]: Colorless liquid (68 mg, 85%); ^1^H NMR (400 MHz, CDCl_3_) δ 7.74–7.71 (m, 2H), 7.35–7.29 (m, 2H), 2.55 (s, 3H), 2.37 (s, 3H); ^13^C NMR (100 MHz, CDCl_3_) δ 198.2, 138.1, 137.0, 133.7, 128.6, 128.3, 125.4, 26.5, 21.1.

*1-(o-tolyl)ethan-1-one* (**2m**) [56]: Colorless liquid (60 mg, 75%); ^1^H NMR (400 MHz, CDCl_3_) δ 8.02 (d, *J* = 7.6 Hz, 1H), 7.45 (t, *J* = 7.6 Hz, 1H), 7.35–7.30 (m, 2H), 2.65 (s, 3H), 2.61 (s, 3H); ^13^C NMR (100 MHz, CDCl_3_) δ 201.6, 138.3, 137.5, 131.9, 131.4, 129.3, 125.6, 29.4, 21.5.

*1,1’-(1,4-phenylene)bis(ethan-1-one)* (**2n**) [56]: Grayish white solid (76 mg, 78%); ^1^H NMR (400 MHz, CDCl_3_) δ 8.02 (s, 4H), 2.63 (s, 6H); ^13^C NMR (100 MHz, CDCl_3_) δ 197.5, 140.1, 128.4, 26.9.

*1-(3,4-dimethylphenyl)ethan-1-one* (**2o**) [58]: Colorless liquid (82 mg, 92%); ^1^H NMR (400 MHz, CDCl_3_) δ 7.69 (s, 1H), 7.65 (d, *J* = 7.6 Hz, 1H), 7.16 (d, *J* = 7.6 Hz, 1H), 2.52 (s, 3H), 2.27 (s, 6H); ^13^C NMR (100 MHz, CDCl_3_) δ 197.8, 142.4, 136.6, 134.9, 129.5, 129.2, 125.9, 26.3, 19.7, 19.5.

*1-(3,4-dichlorophenyl)ethan-1-one* (**2p**) [60]: white solid (102 mg, 90%); ^1^H NMR (400 MHz, CDCl_3_) δ 8.00 (s, 1H), 7.76 (d, *J* = 8.4 Hz, 1H), 7.53 (d, *J* = 8.4 Hz, 1H), 2.57 (s, 3H); ^13^C NMR (100 MHz, CDCl_3_) δ 195.6, 137.6, 136.5, 133.2, 130.7, 130.3, 127.3, 26.5.

*2,3-dihydro-1H-inden-1-one* (**2q**) [56]: Light yellow solid (59 mg, 74%); ^1^H NMR (400 MHz, CDCl_3_) δ 7.74 (d, *J* = 8.0 Hz, 1H), 7.57 (t, *J* = 7.6 Hz, 1H), 7.46 (d, *J* = 7.2 Hz, 1H), 7.35 (t, *J* = 7.2 Hz, 1H), 3.12 (t, 6.0 Hz, 2H), 2.67 (t, *J* = 6.0 Hz, 2H); ^13^C NMR (100 MHz, CDCl_3_) δ 207.0, 155.1, 137.0, 134.5, 127.2, 126.6, 123.6, 36.1, 25.7.

*9H-fluoren-9-one* (**2r**) [56]: Yellow solid (103 mg, 95%); ^1^H NMR (400 MHz, CDCl_3_) δ 7.59 (d, *J* = 7.2 Hz, 2H), 7.44–7.39 (m, 4H), 7.25–7.21 (m, 2H); ^13^C NMR (100 MHz, CDCl_3_) δ 193.8, 144.3, 134.5, 134.0, 128.9, 124.1, 120.2.

*2-amino-9H-fluoren-9-one* (**2s**) [60]: Brown solid (95 mg, 81%); ^1^H NMR (400 MHz, DMSO-*d6*) δ 7.43–7.39 (m, 3H), 7.35 (d, *J* = 8.0 Hz, 1H), 7.14–7.09 (m, 1H), 6.78 (d, *J* = 2.4 Hz, 1H), 6.66 (dd, *J* = 8.0, 2.0 Hz, 1H); ^13^C NMR (100 MHz, DMSO-*d6*) δ 194.7, 150.9, 146.4, 135.9, 135.5, 127.4, 124.2, 122.6, 119.7, 119.0, 109.9.

*9H-xanthen-9-one* (**2t**) [56]: Grayish white solid (108 mg, 92%); ^1^H NMR (400 MHz, CDCl_3_) δ 8.33 (d, *J* = 8.0 Hz, 2H), 7.71 (t, *J* = 7.6 Hz, 2H), 7.47 (d, *J* = 8.4 Hz, 2H), 7.36 (t, *J* = 7.6 Hz, 2H); ^13^C NMR (100 MHz, CDCl_3_) δ 177.2, 156.1, 134.8, 126.7, 123.8, 121.8, 117.9.

*1-(naphthalen-1-yl)ethan-1-one* (**2u**) [56]: Colorless liquid (84 mg, 82%); ^1^H NMR (400 MHz, CDCl_3_) δ 8.79 (d, *J* = 8.8 Hz, 1H), 7.96 (d, *J* = 8.4 Hz, 1H), 7.91 (d, *J* = 7.2 Hz, 1H), 7.86 (d, *J* = 8.4 Hz, 1H), 7.61 (t, *J* = 7.6 Hz, 1H), 7.53 (t, *J* = 7.6 Hz, 1H), 7.47 (t, *J* = 8.0 Hz, 1H), 2.73 (s, 3H); ^13^C NMR (100 MHz, CDCl_3_) δ 201.7, 135.2, 133.8, 132.9, 130.0, 128.6, 128.3, 127.9, 126.3, 125.9, 124.2, 29.8.

*1-(naphthalen-2-yl)ethan-1-one* (**2v**) [56]: White solid (87 mg, 85%); ^1^H NMR (400 MHz, CDCl_3_) δ 8.46 (s, 1H), 8.03 (d, *J* = 8.4 Hz, 1H), 7.96 (d, *J* = 8.0 Hz, 1H), 7.90–7.86 (m, 2H), 7.62–7.53 (m, 2H), 2.72 (s, 3H); ^13^C NMR (100 MHz, CDCl_3_) δ 198.1, 135.5, 134.4, 132.5, 130.1, 129.5, 128.4, 128.4, 127.7, 126.7, 123.8, 26.6.

*1-(pyridin-4-yl)ethan-1-one* (**2w**) [56]: Colorless liquid (40 mg, 56%); ^1^H NMR (400 MHz, CDCl_3_) δ 8.74 (d, *J* = 4.8 Hz, 2H), 7.66 (d, *J* = 4.8 Hz, 2H), 2.56 (s, 3H); ^13^C NMR (100 MHz, CDCl_3_) δ 197.2, 150.8, 142.5, 121.0, 26.5.

*1-(thiophen-2-yl)ethan-1-one* (**2x**) [56]: Light yellow liquid (49 mg, 65%);^1^H NMR (400 MHz, CDCl_3_) δ 7.66 (d, *J* = 3.6 Hz, 1H), 7.60 (d, *J* = 5.2 Hz, 1H), 7.08 (t, *J* = 4.0 Hz, 1H), 2.51 (s, 3H); ^13^C NMR (100 MHz, CDCl_3_) δ 190.6, 144.3, 133.7, 132.4, 128.0, 26.7.

*1-(benzo[b]thiophen-5-yl)ethan-1-one* (**2y**) [61]: White solid (93 mg, 88%); ^1^H NMR (400 MHz, CDCl_3_) δ 7.94 (s, 1H), 7.88 (t, *J* = 8.4 Hz, 2H), 7.47 (t, *J* = 7.6 Hz, 1H), 7.41 (t, *J* = 7.2 Hz, 1H), 2.67 (s, 3H); ^13^C NMR (100 MHz, CDCl_3_) δ 192.2, 143.9, 142.6, 139.1, 129.6, 127.4, 125.9, 125.0, 123.0, 26.8.

*propiophenone* (**2z**) [56]: Colorless liquid (57 mg, 71%); ^1^H NMR (400 MHz, CDCl_3_) δ 7.96 (d, *J* = 7.6 Hz, 2H), 7.55 (t, *J* = 7.2 Hz, 1H), 7.45 (t, *J* = 7.6 Hz, 2H), 3.01 (q, *J* = 7.2 Hz, 2H), 1.23 (t, *J* = 7.2 Hz, 3H); ^13^C NMR (100 MHz, CDCl_3_) δ 200.8, 136.9, 132.8, 128.5, 127.9, 31.7, 8.2.

*1-phenylbutan-1-one* (**2aa**) [61]: Colorless liquid (61 mg, 69%); ^1^H NMR (400 MHz, CDCl_3_) δ 7.97 (d, *J* = 7.6 Hz, 2H), 7.56 (t, *J* = 7.2 Hz, 1H), 7.46 (t, *J* = 7.6 Hz, 2H), 2.96 (t, *J* = 7.2 Hz, 2H), 1.83–1.74 (m, 2H), 1.02 (t, *J* = 7.6 Hz, 3H); ^13^C NMR (100 MHz, CDCl_3_) δ 200.3, 137.0, 132.8, 128.4, 127.9, 40.4, 17.7, 13.8.

*1-phenyloctan-1-one* (**2ab**) [60]: Colorless liquid (76 mg, 62%); ^1^H NMR (400 MHz, CDCl_3_) δ 7.96 (d, *J* = 7.2 Hz, 2H), 7.55 (t, *J* = 7.2 Hz, 1H), 7.46 (t, *J* = 7.6 Hz, 2H), 2.96 (t, *J* = 7.2 Hz, 2H), 1.77–1.70 (m, 2H), 1.36–1.25 (m, 8H), 0.88 (t, *J* = 6.8 Hz, 3H); ^13^C NMR (100 MHz, CDCl_3_) δ 200.7, 137.1, 132.8, 128.5, 128.0, 38.6, 31.7, 29.3, 29.1, 24.4, 22.6, 14.1.

*Benzophenone* (**2ac**) [56]: White solid (100 mg, 92%); ^1^H NMR (400 MHz, CDCl_3_) δ 7.81 (d, *J* = 7.6 Hz, 4H), 7.59 (t, *J* = 7.6 Hz, 2H), 7.48 (t, *J* = 7.6 Hz, 4H); ^13^C NMR (100 MHz, CDCl_3_) δ 196.7, 137.6, 132.4, 130.0, 128.2.

*phenyl(p-tolyl)methanone* (**2ad**) [56]: White solid (100 mg, 85%); ^1^H NMR (400 MHz, CDCl_3_) δ 7.78 (d, *J* = 7.6 Hz, 2H), 7.72 (t, *J* = 7.6 Hz, 2H), 7.56 (t, *J* = 7.2 Hz, 1H), 7.46 (t, *J* = 7.2 Hz, 2H), 7.27 (d, *J* = 8.0 Hz, 2H), 2.43 (s, 3H); ^13^C NMR (100 MHz, CDCl_3_) δ 196.4, 143.2, 137.9, 134.8, 132.1, 130.2, 129.9, 128.9, 128.1, 21.6.

*(4-methoxyphenyl)(phenyl)methanone* (**2ae**) [56]: White solid (114 mg, 90%); ^1^H NMR (400 MHz, CDCl_3_) δ 7.83 (d, *J* = 8.8 Hz, 2H), 7.75 (t, *J* = 7.6 Hz, 2H), 7.56 (t, *J* = 7.6 Hz, 1H), 7.47 (t, *J* = 7.2 Hz, 2H), 6.96 (d, *J* = 8.8 Hz, 2H), 3.89 (s, 3H); ^13^C NMR (100 MHz, CDCl_3_) δ 195.6, 163.2, 138.3, 132.5, 131.9, 130.1, 129.7, 128.2, 113.5, 55.5.

*(4-chlorophenyl)(phenyl)methanone* (**2af**) [56]: White solid (109 mg, 84%); ^1^H NMR (400 MHz, CDCl_3_) δ 7.76 (t, *J* = 8.0 Hz, 4H), 7.59 (t, *J* = 7.6 Hz, 1H), 7.48 (q, *J* = 8.4 Hz, 4H); ^13^C NMR (100 MHz, CDCl_3_) δ 195.4, 138.8, 137.2, 135.8, 132.6, 131.4, 129.9, 128.6, 128.3.

*Anthracene-9,10-dione* (**2ag**) [56]: Yellow solid (22 mg, 18%); ^1^H NMR (400 MHz, CDCl_3_) δ 8.32 (dd, *J* = 5.2, 3.2 Hz, 4H), 7.81 (dd, *J* = 5.2, 3.2 Hz, 4H); ^13^C NMR (100 MHz, CDCl_3_) δ 183.2, 134.1, 133.5, 127.2.

*Benzoic acid* (**2ah**) [46]: White solid (72 mg, 99%); ^1^H NMR (400 MHz, DMSO-d6) δ 12.99 (s, 1H), 7.95 (d, *J* = 6.8 Hz, 2H), 7.62 (t, *J* = 7.2 Hz, 1H), 7.49 (t, *J* = 7.6 Hz, 2H); ^13^C NMR (100 MHz, DMSO-d6) δ 167.9, 133.4, 131.3, 129.8, 129.1.

*Methyl 2-(1-(4-acetylbenzoyl)-5-methoxy-2-methyl-1H-indol-3-yl)acetate* (**2ai**) [56]: White solid (175 mg, 77%); ^1^H NMR (400 MHz, CDCl_3_) δ 8.06 (d, *J* = 8.0 Hz, 2H), 7.79 (d, *J* = 7.6 Hz, 2H), 6.96 (s, 1H), 6.85 (d, *J* = 8.8 Hz, 1H), 6.65 (d, *J* = 8.8 Hz, 1H), 3.83 (s, 3H), 3.71 (s, 3H), 3.67 (s, 2H), 2.68 (s, 3H), 2.36 (s, 3H); ^13^C NMR (100 MHz, CDCl_3_) δ 197.3, 171.3, 168.5, 156.2, 139.8, 139.6, 135.9, 130.8, 130.7, 129.7, 128.6, 115.1, 112.9, 111.7, 101.4, 55.7, 52.2, 30.1, 29.7, 26.9, 13.5.

*(3S,8S,9S,10R,13R,14S,17R)-10,13-dimethyl-17-((R)-6-methylheptan-2-yl)-2,3,4,7,8,9,10,11,12,13,14,15,16,17-tetradecahydro-1H-cyclopenta[a]phenanthren-3-yl 4-acetylbenzoate* (**2aj**): White solid (233 mg, 73%); ^1^H NMR (400 MHz, CDCl_3_) δ 8.12 (d, *J* = 8.4 Hz, 2H), 8.00 (d, *J* = 8.4 Hz, 2H), 5.43 (d, *J* = 4.4 Hz, 1H), 4.92–4.84 (m, 1H), 2.65 (s, 3H), 2.48 (d, *J* = 7.6 Hz, 2H), 2.04–1.98 (m, 3H), 1.86–1.74 (m, 2H), 1.58–1.43 (m, 6H), 1.37–1.21 (m, 6H), 1.19–1.09 (m, 5H), 1.07 (s, 3H), 1.06–0.96 (m, 4H), 0.92 (d, *J* = 6.8 Hz, 3H), 0.87 (d, *J* = 2.0 Hz, 3H), 0.86 (d, *J* = 2.0 Hz, 3H), 0.69 (s, 3H); ^13^C NMR (100 MHz, CDCl_3_) δ 197.6, 165.1, 140.0, 139.4, 134.6, 129.8, 128.1, 123.0, 75.2, 56.7, 56.1, 50.0, 42.3, 39.5, 36.6, 35.8, 31.8, 28.0, 26.9, 23.8, 22.8, 22.6, 19.4, 18.7, 11.9. HRMS (ESI) calcd for C_36_H_52_O_3_Na [M + Na]^+^: 555.3809, found 555.3818.

*(3S,8R,9S,10R,13S,14S)-10,13-dimethyl-17-oxo-2,3,4,7,8,9,10,11,12,13,14,15,16,17-tetradecahydro-1H-cyclopenta[a]phenanthren-3-yl 4-acetylbenzoate* (**2ak**): White solid (195 mg, 75%); ^1^H NMR (400 MHz, CDCl_3_) δ 8.12 (d, *J* = 8.4 Hz, 2H), 8.00 (d, *J* = 8.4 Hz, 2H), 5.47 (d, *J* = 4.8 Hz, 1H), 4.93–4.84 (m, 1H), 2.65 (s, 3H), 2.51–2.46 (m, 2H), 2.17–2.08 (m, 2H), 1.98–1.92 (m, 2H), 1.89–1.78 (m, 2H), 1.73–1.67 (m, 4H), 1.55–1.41 (m, 2H), 1.34–1.25 (m, 4H), 1.10 (s, 3H), 0.96 (t, *J* = 7.2 Hz, 1H), 0.90 (s, 3H); ^13^C NMR (100 MHz, CDCl_3_) δ 197.7, 165.1, 140.1, 139.7, 134.5, 129.8, 128.2, 122.2, 74.9, 51.7, 50.2, 47.6, 38.1, 37.0, 36.8, 35.9, 31.5, 31.4, 30.8, 27.8, 26.9, 21.9, 20.4, 19.4, 13.6. HRMS (ESI) calcd for C_28_H_34_O_4_Na [M + Na]^+^: 457.2349, found 457.2354.

*Isopropyl 2-(4-(4-acetylbenzoyl)phenoxy)-2-methylpropanoate* (**2al**) [56]: White solid (152 mg, 69%); ^1^H NMR (400 MHz, CDCl_3_) δ 8.03 (d, *J* = 8.0 Hz, 2H), 7.77 (dd, *J* = 19.2, 8.0 Hz, 4H), 6.86 (d, *J* = 8.4 Hz, 2H), 5.12–5.04 (m, 1H), 2.65 (s, 3H), 1.65 (s, 6H), 1.19 (d, *J* = 6.4 Hz, 6H); ^13^C NMR (100 MHz, CDCl_3_) δ 197.5, 194.6, 173.0, 160.0, 142.0, 139.2, 132.1, 129.8, 129.6, 128.1, 117.2, 79.4, 69.3, 26.8, 25.3, 21.5.

*Anthracene* (**4a**) [62]: Grayish white solid (83 mg, 78%); ^1^H NMR (400 MHz, CDCl_3_) δ 8.44 (s, 2H), 8.02 (q, *J* = 3.2 Hz, 4H), 7.48 (q, *J* = 3.2 Hz, 4H); ^13^C NMR (100 MHz, CDCl_3_) δ 131.7, 128.1, 126.2, 125.3.

*Chalcone* (**4b**) [61]: Yellow solid (97 mg, 78%);^1^H NMR (400 MHz, CDCl_3_) δ 8.03 (d, *J* = 7.6 Hz, 2H), 7.82 (d, *J* = 15.6 Hz, 1H), 7.66–7.42 (m, 9H); ^13^C NMR (100 MHz, CDCl_3_) δ 190.6, 144.9, 138.2, 134.9, 132.8, 130.5, 128.9, 128.6, 128.5, 128.4, 122.1.

*2-phenyl-1H-benzo[d]imidazole* (**4c**) [61]: White solid (72 mg, 62%); ^1^H NMR (400 MHz, DMSO-*d*_6_) δ 8.15 (d, *J* = 7.2 Hz, 2H), 7.60–7.48 (m, 5H), 7.24–7.19 (m, 2H); ^13^C NMR (100 MHz, DMSO-*d*_6_) δ 152.1, 130.9, 130.7, 129.9, 127.3, 123.2.

*N-phenylacetamide* (**4d**) [61]: Grayish white solid (49 mg, 60%); ^1^H NMR (400 MHz, CDCl_3_) δ 8.49 (s, 1H), 8.05–8.03 (m, 2H), 7.85 (d, *J* = 8.0 Hz, 2H), 7.55–7.48 (m, 4H), 2.82 (s, 3H); ^13^C NMR (100 MHz, CDCl_3_) δ 208.2, 136.7, 131.0, 128.8, 128.2, 126.8, 126.6, 125.5, 124.3, 33.8.

## 4. Conclusions

We have presented an efficient and clean approach for the synthesis of ketones from alkyl aromatics through the use of bis(2-butoxyethyl) ether as the catalyst and reaction medium and O_2_ as the sole oxidant. This new oxidation protocol can tolerate various functional groups and is suitable for large-scale synthesis. Notably, diverse target ketones were prepared in moderate to high yields in the absence of an external catalyst or additive. The late-stage functionalization of drug derivatives with the corresponding ketones and one-pot sequential chemical conversions to ketone downstream products that we have demonstrated here further broadens their application prospects, particularly in the pharmaceutical industry. Furthermore, by monitoring the reaction progress and conducting mechanistic experiments, we developed a bis(2-butoxyethyl) ether-promoted O_2_-mediated oxidation pathway for alkyl aromatics to ketones.

## Figures and Tables

**Table 1:** Optimization of reaction conditions ^a^.

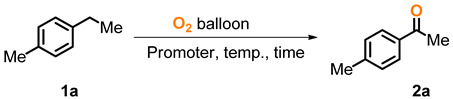					
Entry	Promoter (eq.)	[O]	T/°C	Time	Yield ^b^ (%)
1	2-Methoxyethyl ether (1)	O_2_	120	15 h	63
2	Bis(methoxypropyl) ether (1)	O_2_	120	15 h	60
3	Triethylene glycol dimethyl ether (1)	O_2_	120	15 h	37
4	Tetraethylene glycol dimethyl ether (1)	O_2_	120	15 h	40
5	2-Ethoxyethyl ether (1)	O_2_	120	15 h	65
6	Bis(2-butoxyethyl) ether (1)	O_2_	120	15 h	70
7	1,2-Dibutoxyethane (1)	O_2_	120	15 h	47
**8**	Methoxypropoxypropanol (1)	O_2_	120	15 h	31
9	Bis(2-butoxyethyl) ether (1)	O_2_	130	15 h	77
10	Bis(2-butoxyethyl) ether (1)	O_2_	140	15 h	90
11	Bis(2-butoxyethyl) ether (1)	O_2_	150	15 h	92
12	Bis(2-butoxyethyl) ether (1)	O_2_	110	15 h	31
13	Bis(2-butoxyethyl) ether (1)	O_2_	100	15 h	-
14	Bis(2-butoxyethyl) ether (1)	O_2_	r.t.	15 h	-
15	Bis(2-butoxyethyl) ether (3)	O_2_	150	15 h	91
16	Bis(2-butoxyethyl) ether (2)	O_2_	150	15 h	97
17	Bis(2-butoxyethyl) ether (0.5)	O_2_	150	15 h	76
18	Bis(2-butoxyethyl) ether (2)	O_2_	150	18 h	97
19 ^c^	Bis(2-butoxyethyl) ether (2)	O_2_	150	15 h	97
20	Bis(2-butoxyethyl) ether (2)	N_2_	150	15 h	-
21	Bis(2-butoxyethyl) ether (2)	air	150	15 h	58
22	-	O_2_	150	15 h	-

^a^ Conditions: unless others noted, **1a** (0.6 mmol, 1 eq.), Promoter (2 eq.), 150 °C, 15 h. ^b^ Estimated based GC-MS results. ^c^ This oxidation was carried out in the dark.

**Table 2:** Reaction scope.

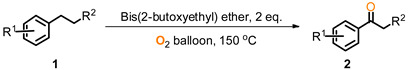
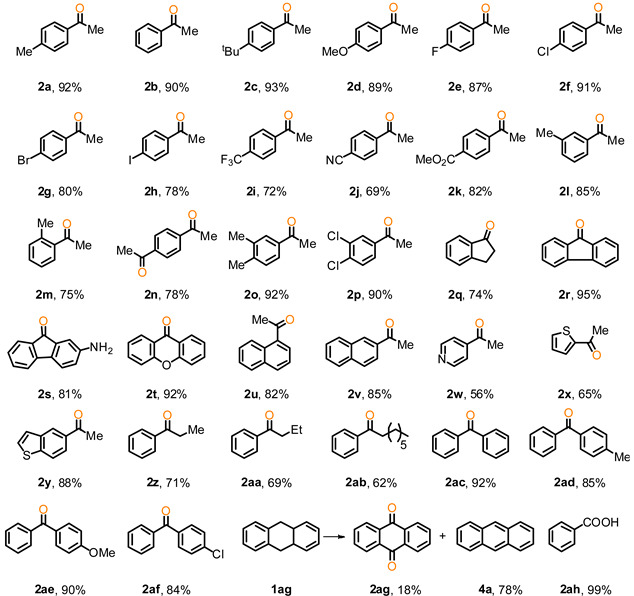
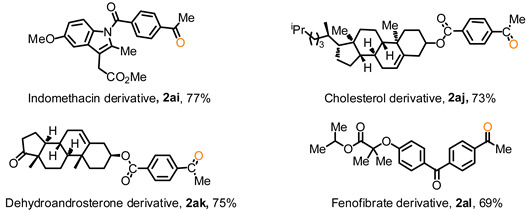

Conditions: **1a** (0.6 mmol, 1 eq.), bis(2-butoxyethyl) ether (2 eq.), O_2_ balloon, 150 °C, 15 h, isolated yields.

## Data Availability

The data presented in this study are available on request from the corresponding authors.

## Supplementary Materials

The following supporting information can be downloaded at: https://www.mdpi.com/article/10.3390/molecules29204909/s1, Section S1: General information; Section S2: Experimental procedure; Section S3: Mechanism Research; Copies of the ^1^H NMR and ^13^C NMR spectra for compounds **2a**–**2ak** and **4a**–**4d**.
