# Supplementary material for: Bis(2-butoxyethyl) Ether-Promoted O2-Mediated Oxidation of Alkyl Aromatics to Ketones under Clean Conditions
Source: Molecules. 2024 Oct 17;29(20):4909. doi: 10.3390/molecules29204909 (PMC11510689; doi:10.3390/molecules29204909)
Supplement: Supplementary file 1 [file molecules-29-04909-s001.zip › molecules-3254726-supplementary.pdf]

Bis(2-butoxyethyl) ether-promoted O_2-mediated oxidation of alkyl aromatics to ketone under clean conditions

Yangyang Xie[1], Zeping Li[1], Xudong, Xu[1], Han Jiang[1], Keyi Chen[1], Jinhua Ou[1*],

Kaijian Liu[1*], Yihui Zhou[2,3*], Kejun Luo[4*]

[1]Department of Material and Chemical Engineering, Hunan Institute of Technology, Hengyang, 421002, China.

[2]College of Chemistry and Chemical Engineering, Hunan University, Changsha, 410082, China.

[3]Hunan Automotive Engineering Vocational College, 412001, Zhuzhou, China.

[4]Changsha Research Institute of Mining and Metallurgy Co.，Ltd, Changsha, 410012, China.

S1. General information

Unless otherwise specified, all reagents and solvents were obtained from commercial suppliers and used without further purification. All reagents were weighed and handled in air at room temperature. ^{1}H NMR spectra were recorded at 400 MHz and ^{13}C NMR spectra were recorded at 100 MHz by using a Bruker Avance 400 spectrometer. Chemical shifts were calibrated using residual undeuterated solvent as an internal reference (^{1}H NMR: $CDCl_3$ 7.26 ppm, ^{13}C NMR: $CDCl_3$ 77.0 ppm, ^{1}H NMR: DMSO 2.50 ppm, ^{13}C NMR: 40.0 ppm). The following abbreviations were used to describe peak splitting patterns when appropriate: s = singlet, d = doublet, t = triplet, q = quartet, m = multiplet. Chromatographic purifications were carried out on a Biotage Isolera Four instrument. GC-MS were obtained by EI on a Shimadzu GC-MS 2010, Conditions: Flow ripples：1 mL/min; column oven initial temperature 70°C; injection port temperature: 310°C; temperature range: 70~310°C.

S2. Experimental procedure

S2.1 General procedure for the synthesis of ketones 2

A mixture of alkyl benzene **1** (0.6 mmol) and bis(2-butoxyethyl) ether (1.2 mmol) was added to a 15 mL glass tube with an O_2 balloon at room temperature. Then the contents were stirred at 150 °C for 15 hours. The progress of the reaction was monitored by TLC or GC-MS. Upon completion, the reaction was cooled down to room temperature. The reaction mixture was purified by silica gel column chromatography to afford the desired **2**.

S2.2 Oxidation of 1a to 2a

A mixture of 1-ethyl-4-methylbenzene **1a** (1.44 g, 12.0 mol) and bis(2-butoxyeth yl) ether (6.0 mL, 24.0 mmol) was added to a 50 mL round-bottomed flask with an O_2 balloon at room temperature, then the contents were stirred at 150 °C for 18 hours. Upon completion, the reaction was cooled down to room temperature and analyzed by GC-MS. The product was obtained in a 90% yield.

S2.3 One-pot synthesis of chalcone

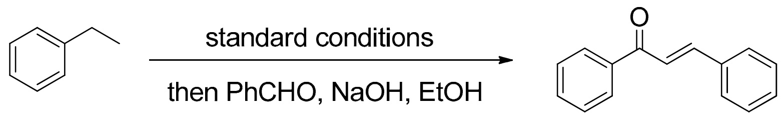

1b **4b**

A mixture of ethylbenzene **1b** (0.6 mmol) and bis(2-butoxyethyl) ether (1.2 mmol) was added to a 15 mL glass tube with an O_2 balloon at room temperature. Then the contents were stirred at 150 °C for 15 hours. The contents were cooled to room temperature, benzaldehyde (0.6 mmol), EtOH (3 mL), sodium hydroxide (0.06 mmol) was added and stirred for 5 hours at room temperature. The progress of the reaction was monitored by TLC. Upon completion, cold stone ether (5 mL) was added and the crude product was obtained. Above crude product is recrystallized in alcohol.

S2.4 One-pot synthesis of 2-phenyl-1*H*-benzo[*d*]imidazole

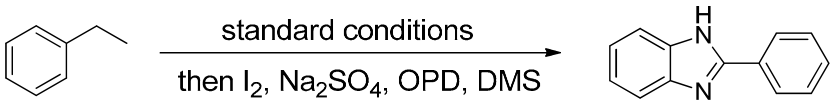

1b **4c**

A mixture of ethylbenzene **1b** (0.6 mmol) and bis(2-butoxyethyl) ether (1.2 mmol) was added to a 15 mL glass tube with an O_2 balloon at room temperature. Then the contents were stirred at 150 °C for 15 hours. The contents were cooled to room temperature. I_2 (0.06 mmol), DMSO (3.0 mL), Na_2SO_4 (3.0 mmol) and benzene-1,2-di amine (OPD, 0.6 mmol) were added to above contents. The reaction mixture was allowed to stir at 120 °C for about 12 h. After completion of the reaction (as monitored by TLC), the mixture was diluted with water and filtered. The filtrate was extracted with EtOAc (4 ×15 mL), and the extract was washed with brine, dried over Na_2SO_4, and evaporated; the crude product was further purified by silica gel column chromatography with petroleum ether/ethyl acetate as eluent. The overall yield was 62%.

S2.5 One-pot synthesis of *N*-phenylacetamide

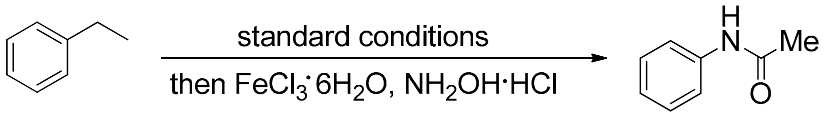

1b **4d**

A mixture of ethylbenzene **1b** (0.6 mmol) and bis(2-butoxyethyl) ether (1.2 mmol) was added to a 15 mL glass tube with an O_2 balloon at room temperature. Then the contents were stirred at 150 °C for 15 hours. The contents were cooled to room temperature. $FeCl_3 \cdot H_2O$ (0.06 mmol) and $NH_2OH \cdot HCl$ (1.8 mmol) were added to above contents under N_2 atmosphere. The reaction mixture was allowed to stir at 70 °C for about 20 h. The reaction was quenched by adding saturated sodium bicarbonate and extracted with CH_2Cl_2. The organic layer was washed with water and brine, dried over $MgSO_4$, filtered and evaporated under reduced pressure to afford the crude product, which was further purified by silica gel column chromatography with petroleum ether/ethyl acetate as eluent. The overall yield was 60%.

S3. Mechanism Research

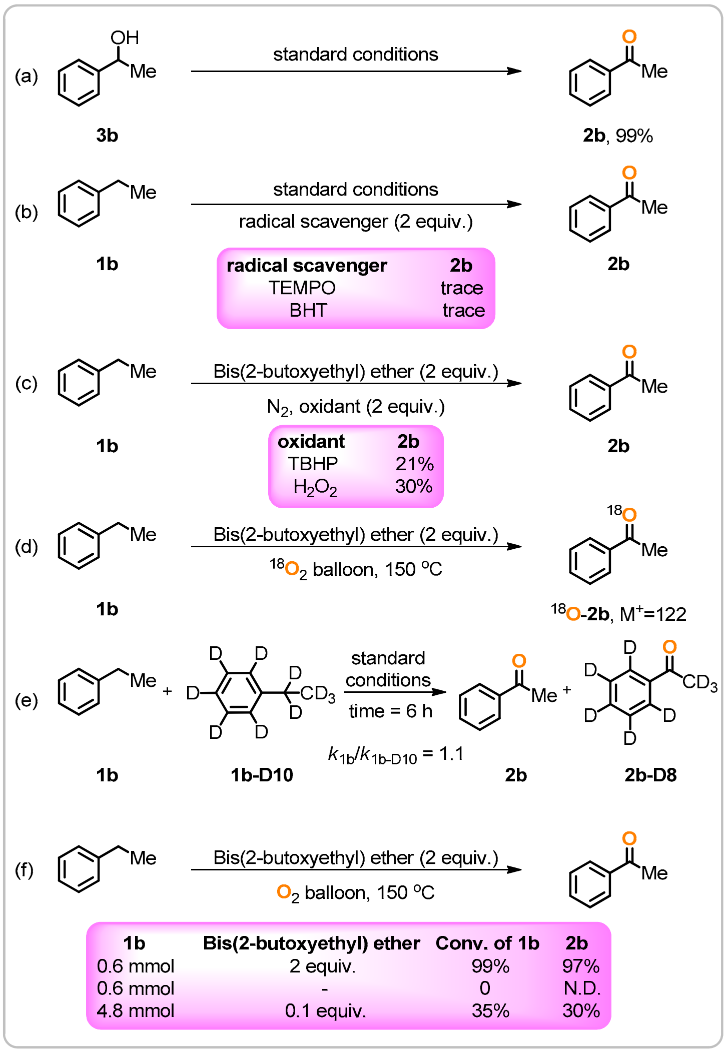

(a) Reaction benzaldedyde under standard conditions:

A mixture of 1-phenylethanol **3b** (0.6 mmol) and bis(2-butoxyethyl) ether (1.2 mmol) was added to a 15 mL glass tube with an O_2 balloon at room temperature, then the contents were stirred at 150 ºC for 15 hours. Upon completion, the reaction was cooled down to room temperature and analyzed by GC-MS. The product **2b** was obtained in a 99% yield.

(b) Radical trapped experiment:

A mixture of ethylbenzene **1b** (0.6 mmol), bis(2-butoxyethyl) ether (1.2 mmol) and 2,2,6,6-tetramethyl-1-piperidyloxy (TEMPO, 186 mg, 1.2 mmol) was added to a 15 mL glass tube with an oxygen balloon at room temperature, then the contents were stirred at 150 ºC for 15 hours. Upon completion, the reaction was cooled down to room temperature and analyzed by GC-MS, which showed trace product was formed.

A mixture of ethylbenzene **1b** (0.6 mmol), bis(2-butoxyethyl) ether (1.2 mmol) and butyleret hydroxytoluen (BHT, 264 mg, 1.2 mmol) was added to a 15 mL glass tube with an oxygen balloon at room temperature, then the contents were stirred at 150 ºC for 15 hours. Upon completion, the reaction was cooled down to room temperature and analyzed by GC-MS, which showed trace product was formed.

(c) Role of oxidant:

A mixture of ethylbenzene **1b** (0.6 mmol), bis(2-butoxyethyl) ether (1.2 mmol) and 2-hydroperoxy-2-methylpropane (TBHP, 115 μL, 1.2 mmol) was added to a 15 mL glass tube with an N_2 balloon at room temperature, then the contents were stirred at 150 ºC for 15 hours. Upon completion, the reaction was cooled down to room temperature and analyzed by GC-MS. The product **2b** was obtained in a 21% yield..

A mixture of ethylbenzene **1b** (0.6 mmol), bis(2-butoxyethyl) ether (1.2 mmol) and hydrogen peroxide (103 μL, 1.2 mmol) was added to a 15 mL glass tube with an N_2 balloon at room temperature, then the contents were stirred at 150 ºC for 15 hours. Upon completion, the reaction was cooled down to room temperature and analyzed by GC-MS. The product **2b** was obtained in a 30% yield..

(d) Role of $^{18}O_2$:

A mixture of ethylbenzene **1b** (0.6 mmol) and bis(2-butoxyethyl) ether (1.2 mmol)

was added to a 15 mL glass tube with an $^{18}O_2$ balloon at room temperature. Then the contents were stirred at 150 °C for 15 hours. Upon completion, the reaction was cooled down to room temperature and analyzed by GC-MS, which showed ^{18}O-**2b** product was formed.

(e) KIE Experiment:

A mixture of ethylbenzene **1b** (0.3 mmol), ethylbenzene-D10 (0.3 mmol) and bis(2-butoxyethyl) ether (1.2 mmol) was added to a 15 mL glass tube with an O_2 balloon at room temperature, then the contents were stirred at 150 °C for 15 hours. Upon completion, the reaction was cooled down to room temperature and a KIE value of 1.1 was obtained.

(f) Role of 1,2-dibutoxyethane:

A mixture of ethylbenzene **1b** (0.6 mmol) and bis(2-butoxyethyl) ether (1.2 mmol) was added to a 15 mL glass tube with an O_2 balloon at room temperature, then the contents were stirred at 150 °C for 15 hours. The reaction was cooled down to room temperature and analyzed by GC-MS. The product **2b** was obtained in a 97% yield.

A mixture of ethylbenzene **1b** (0.6 mmol) was added to a 15 mL glass tube with an O_2 balloon at room temperature, then the contents were stirred at 150 °C for 15 hours. The reaction was cooled down to room temperature and analyzed by GC-MS, No target **2b** was found.

A mixture of ethylbenzene **1b** (4.8 mmol) and bis(2-butoxyethyl) ether (0.48 mmol) was added to a 15 mL glass tube with an O_2 balloon at room temperature, then the contents were stirred at 150 °C for 15 hours. The reaction was cooled down to room temperature and analyzed by GC-MS. The product **2b** was obtained in a 30% yield.

S4. ¹H and ¹³C NMR spectra

¹H NMR spectrum of 1-(*p*-tolyl)ethan-1-one (2a, 400 MHz, CDCl₃)

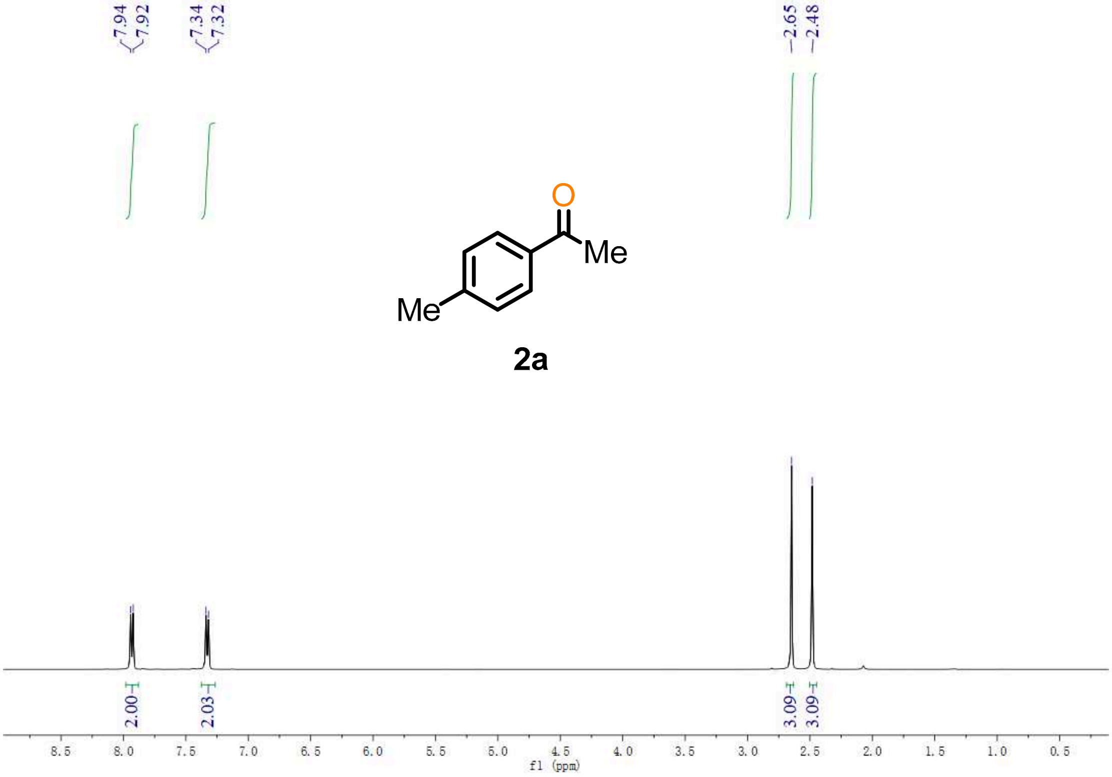

¹³C NMR spectrum of 1-(*p*-tolyl)ethan-1-one (2a, 100 MHz, CDCl₃)

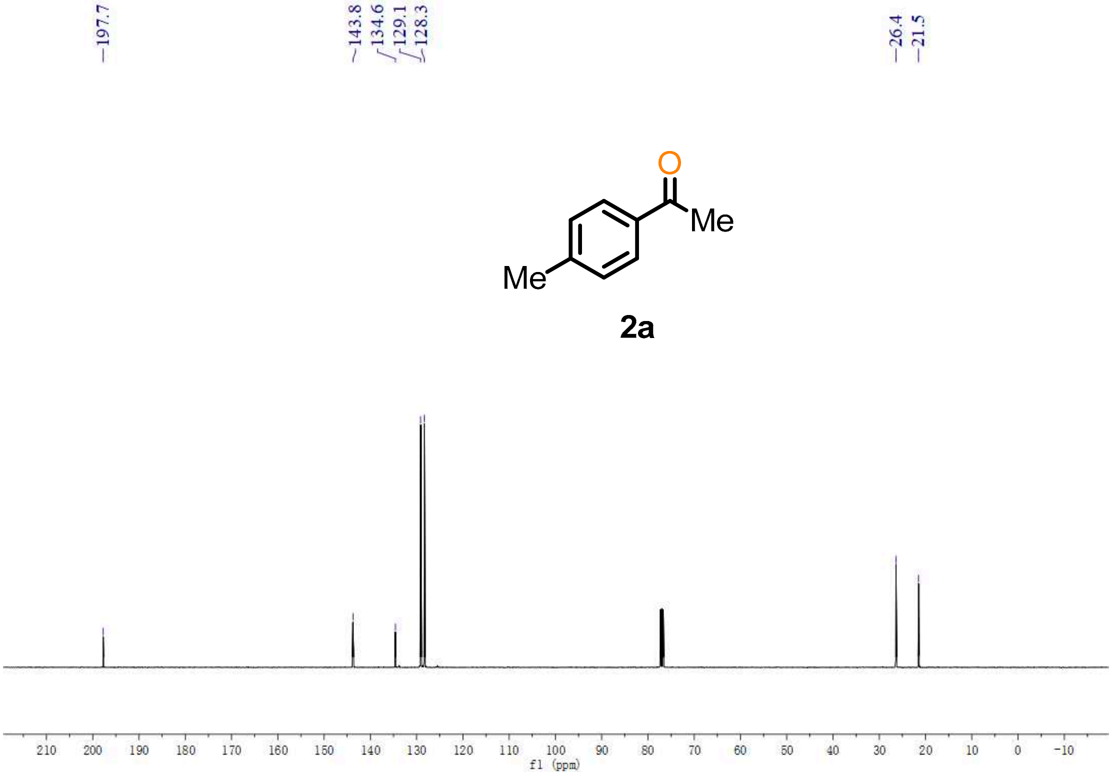

^{1}H NMR spectrum of acetophenone (2b, 400 MHz, $CDCl_3$)

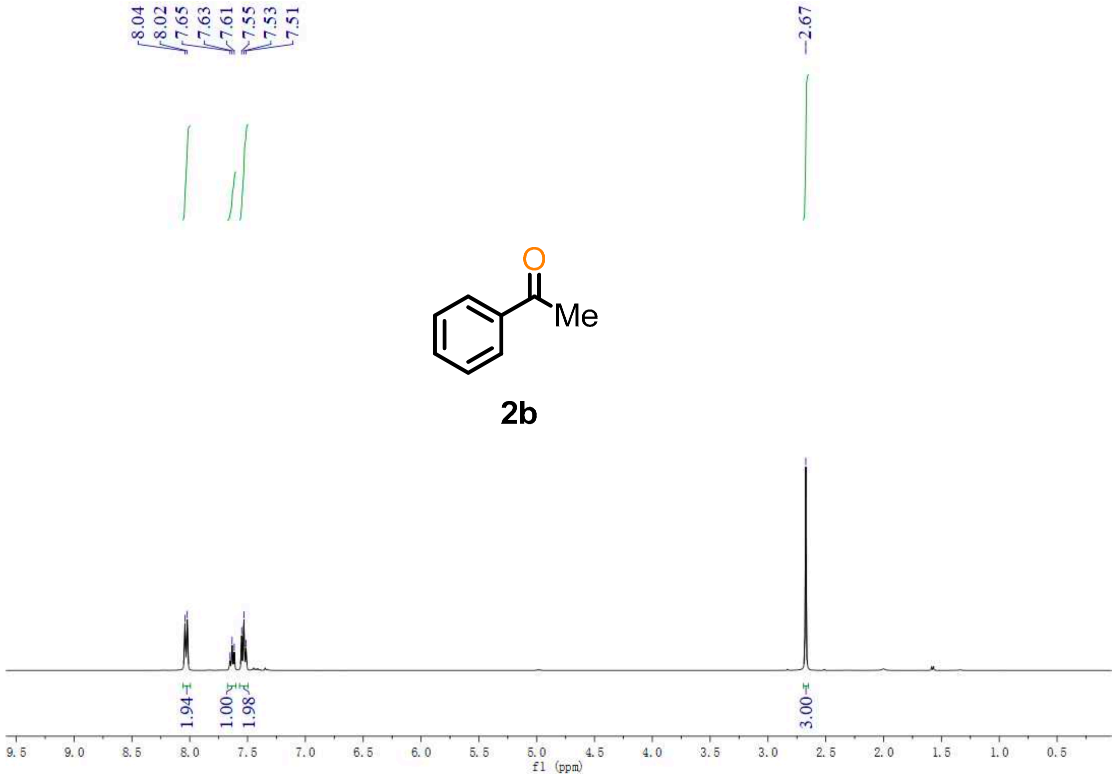

^{13}C NMR spectrum of acetophenone (2b, 100 MHz, $CDCl_3$)

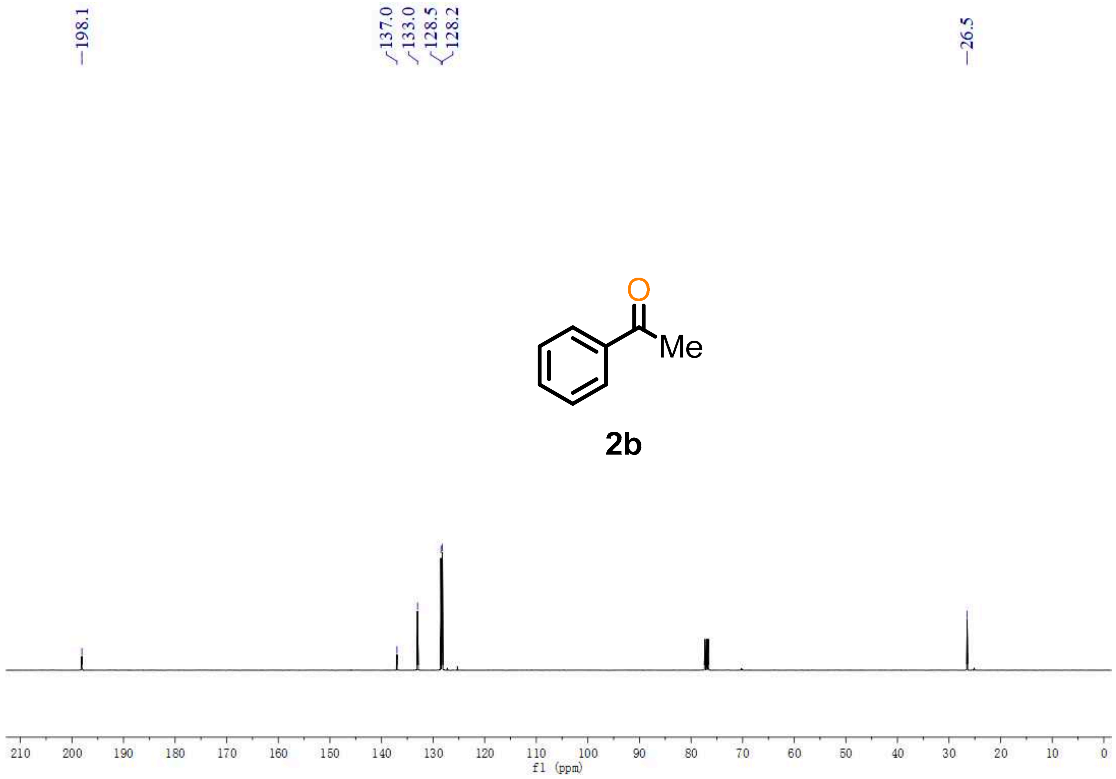

^{1}H NMR spectrum of 1-(4-(*tert*-butyl)phenyl)ethan-1-one (2c, 400 MHz, $CDCl_3$)

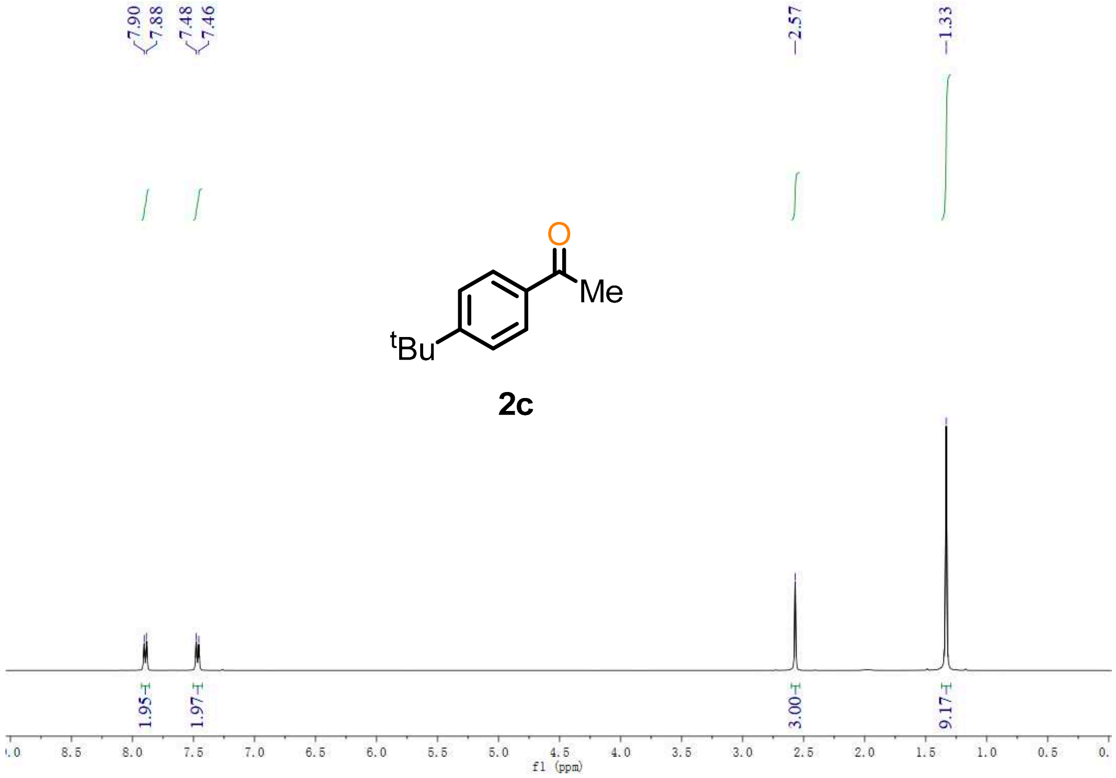

^{13}C NMR spectrum of 1-(4-(*tert*-butyl)phenyl)ethan-1-one (2c, 100 MHz, $CDCl_3$)

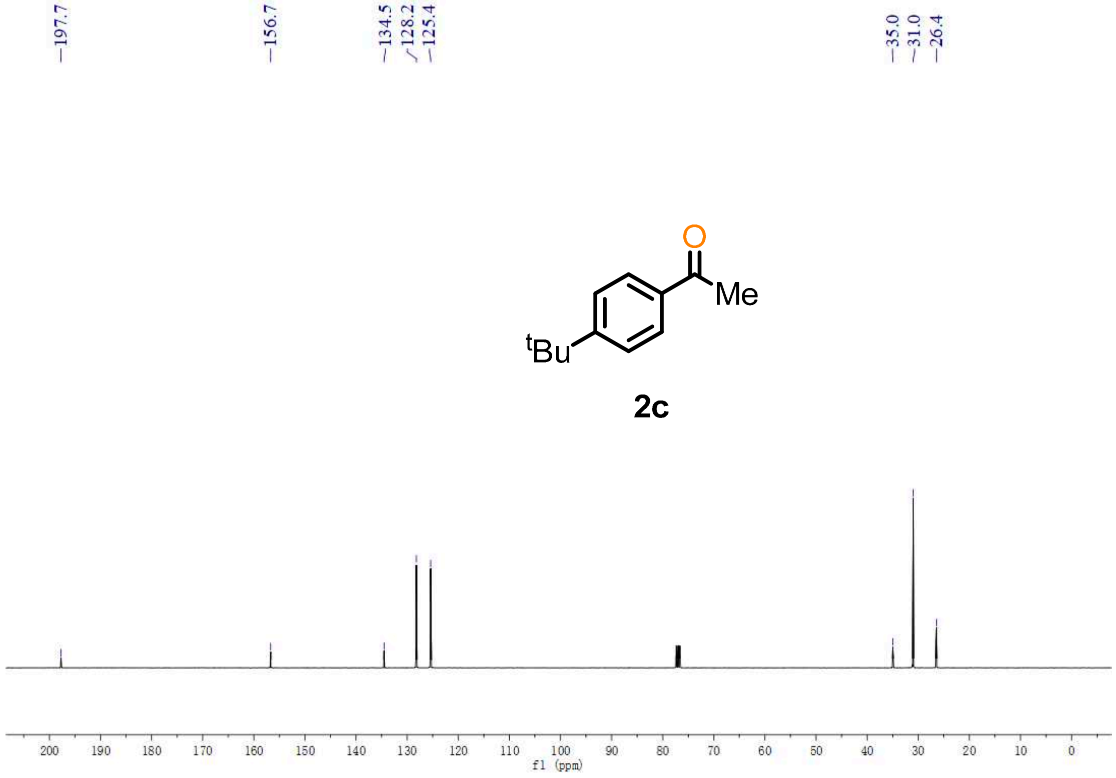

^{1}H NMR spectrum of 1-(4-methoxyphenyl)ethan-1-one (2d, 400 MHz, CDCl$_3$)

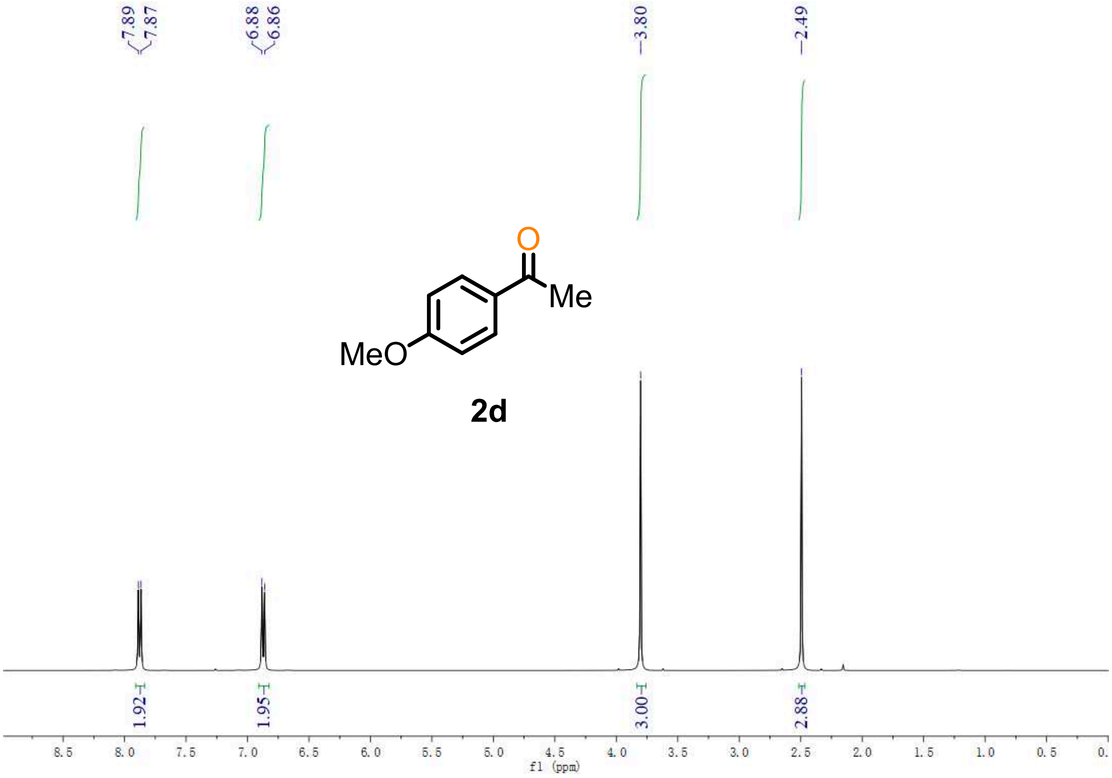

^{13}C NMR spectrum of 1-(4-methoxyphenyl)ethan-1-one (2d, 100 MHz, CDCl$_3$)

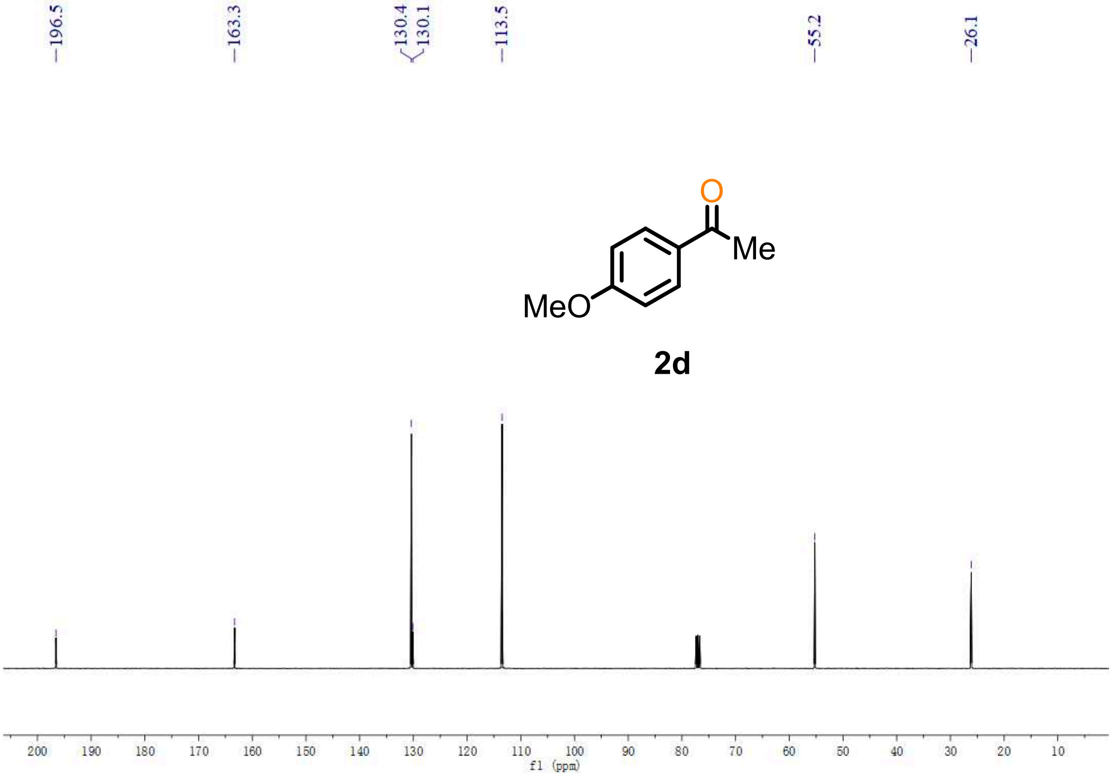

^{1}H NMR spectrum of 1-(4-fluorophenyl)ethan-1-one (2e, 400 MHz, CDCl$_3$)

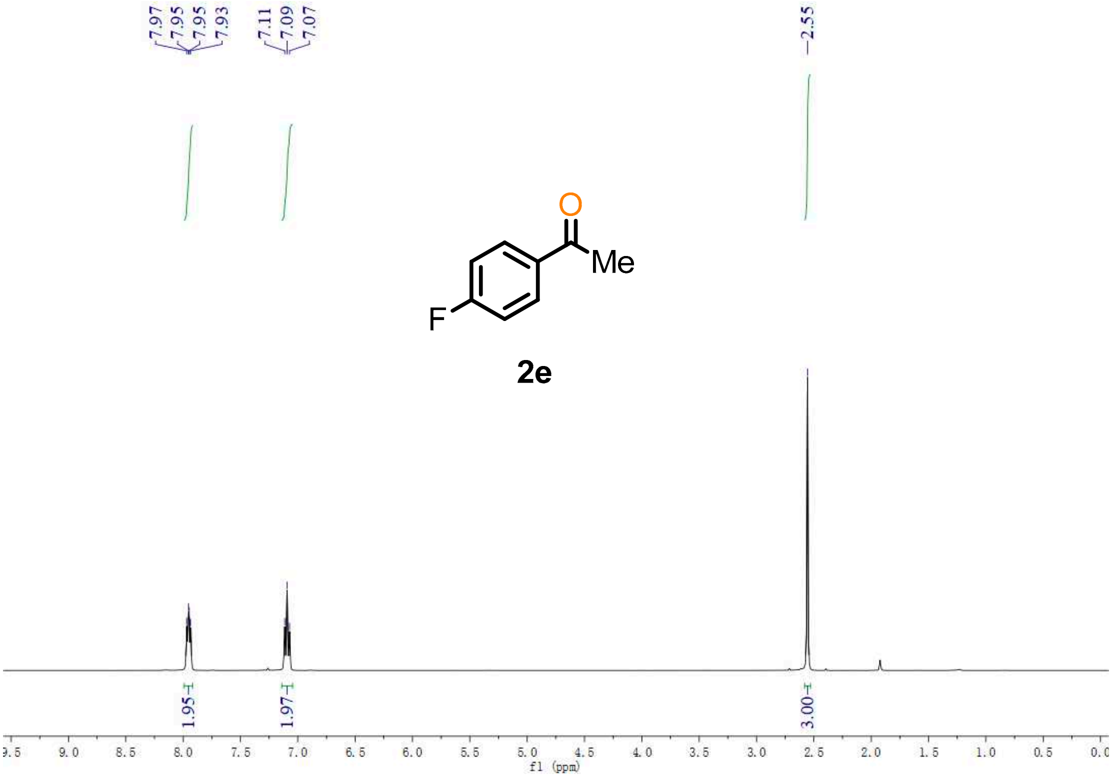

^{13}C NMR spectrum of 1-(4-fluorophenyl)ethan-1-one (2e, 100 MHz, CDCl$_3$)

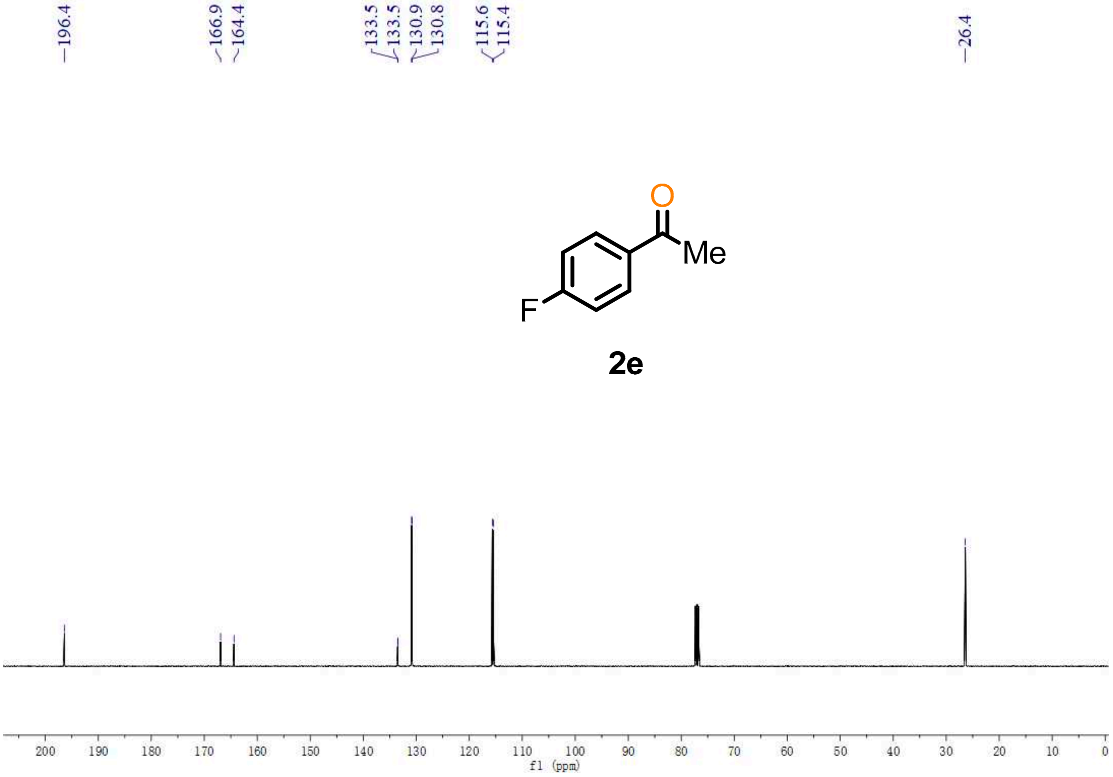

^{19}F NMR spectrum of 1-(4-fluorophenyl)ethan-1-one (2e, 376 MHz, $CDCl_3$)

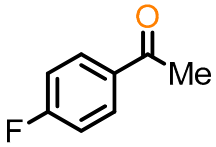

2e

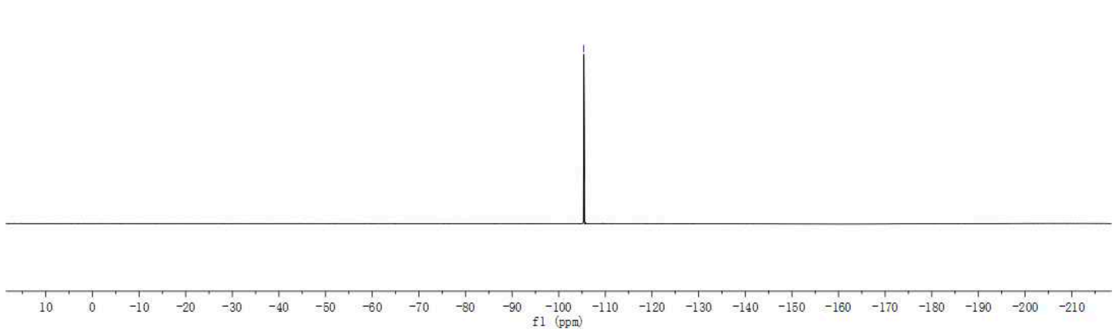

^{1}H NMR spectrum of 1-(4-chlorophenyl)ethan-1-one (2f, 400 MHz, $CDCl_3$)

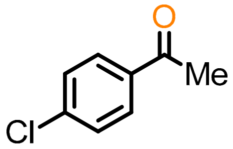

2f

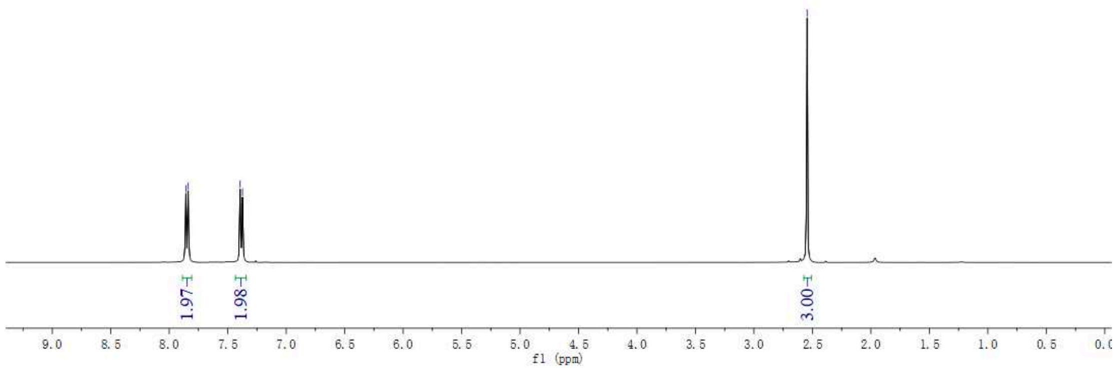

^{13}C NMR spectrum of 1-(4-chlorophenyl)ethan-1-one (2f, 100 MHz, $CDCl_3$)

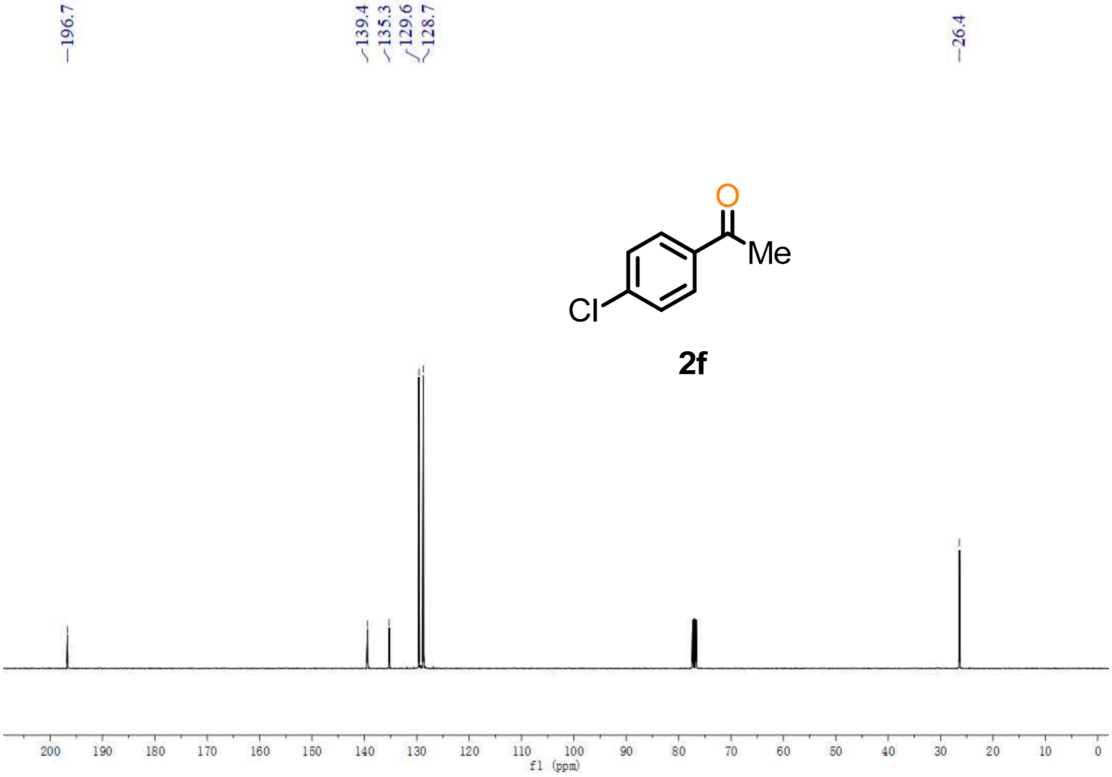

^{1}H NMR spectrum of 1-(4-bromophenyl)ethan-1-one (2g, 400 MHz, $CDCl_3$)

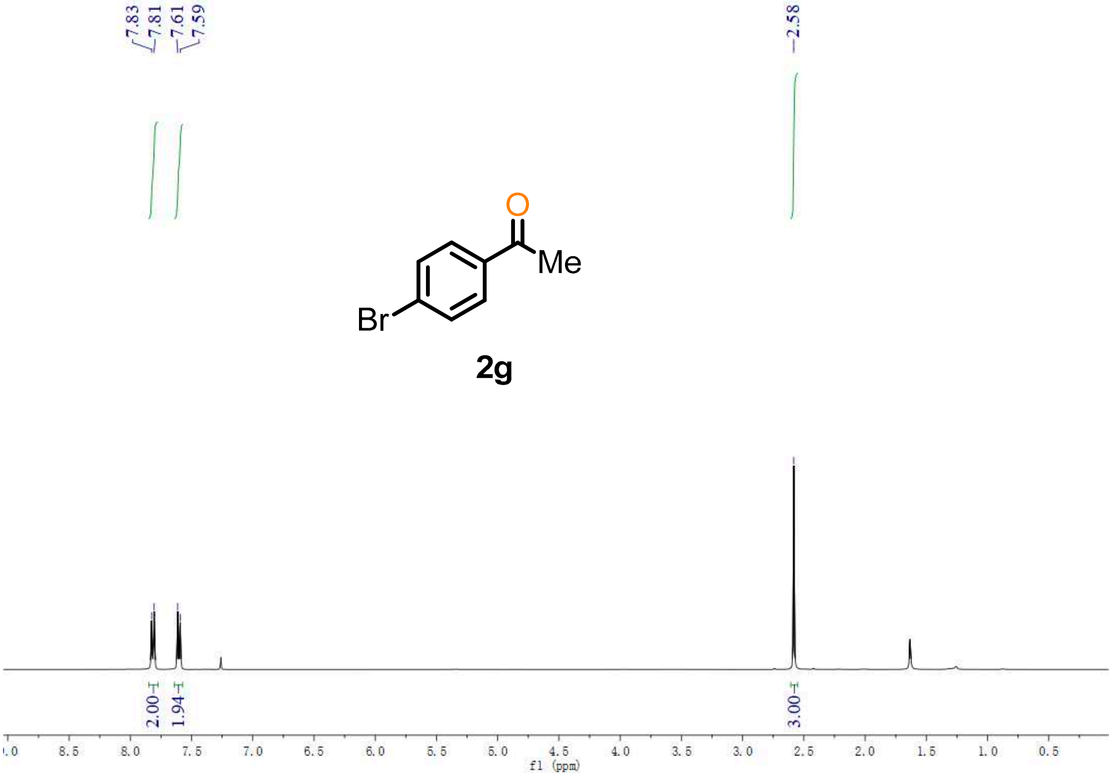

^{13}C NMR spectrum of 1-(4-bromophenyl)ethan-1-one (2g, 100 MHz, $CDCl_3$)

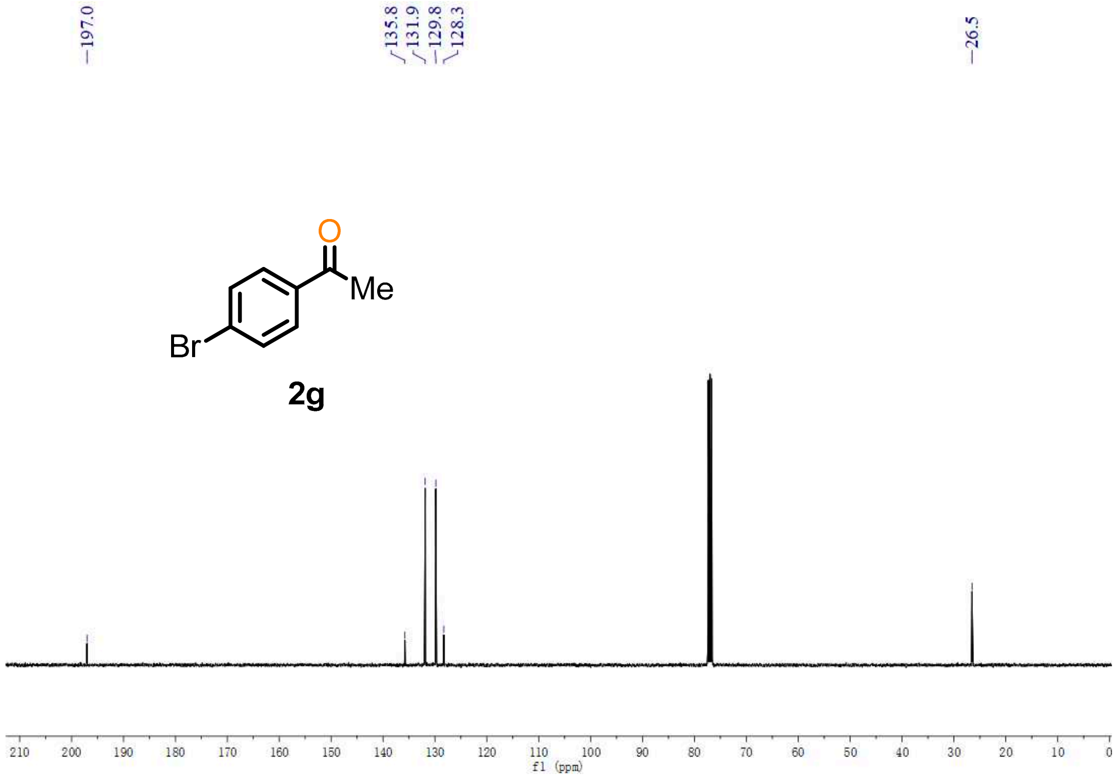

^{1}H NMR spectrum of 1-(4-iodophenyl)ethan-1-one (2h, 400 MHz, $CDCl_3$)

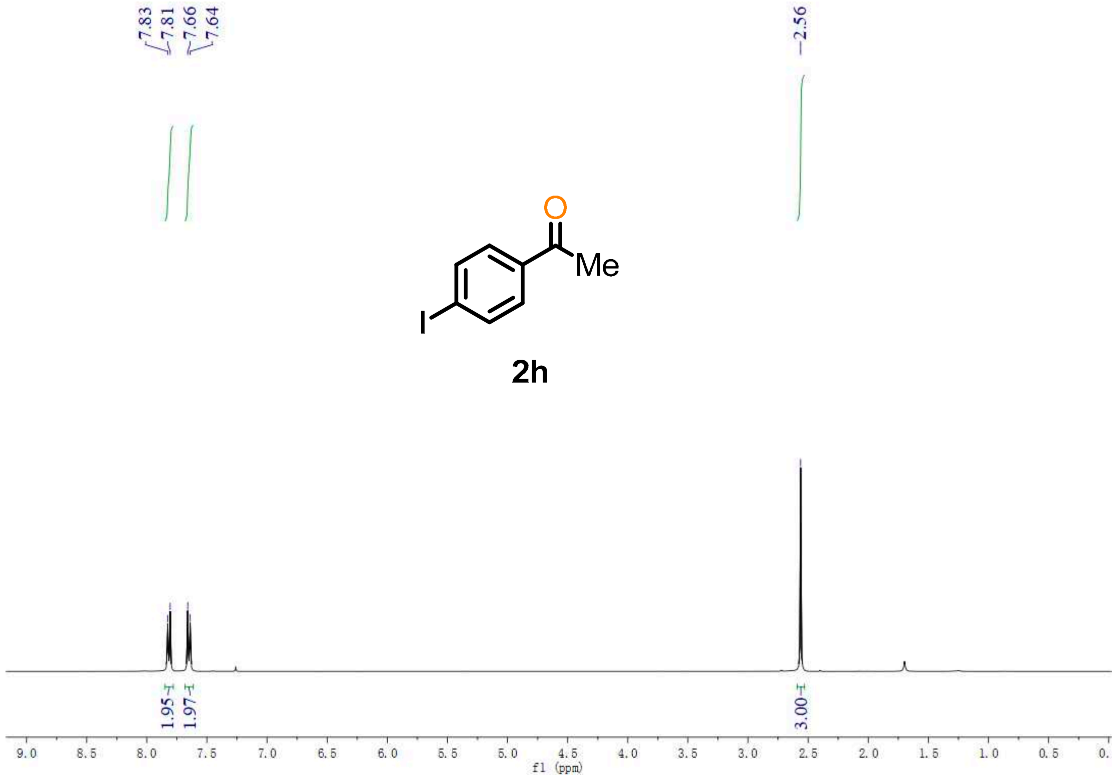

^{13}C NMR spectrum of 1-(4-iodophenyl)ethan-1-one (2h, 100 MHz, $CDCl_3$)

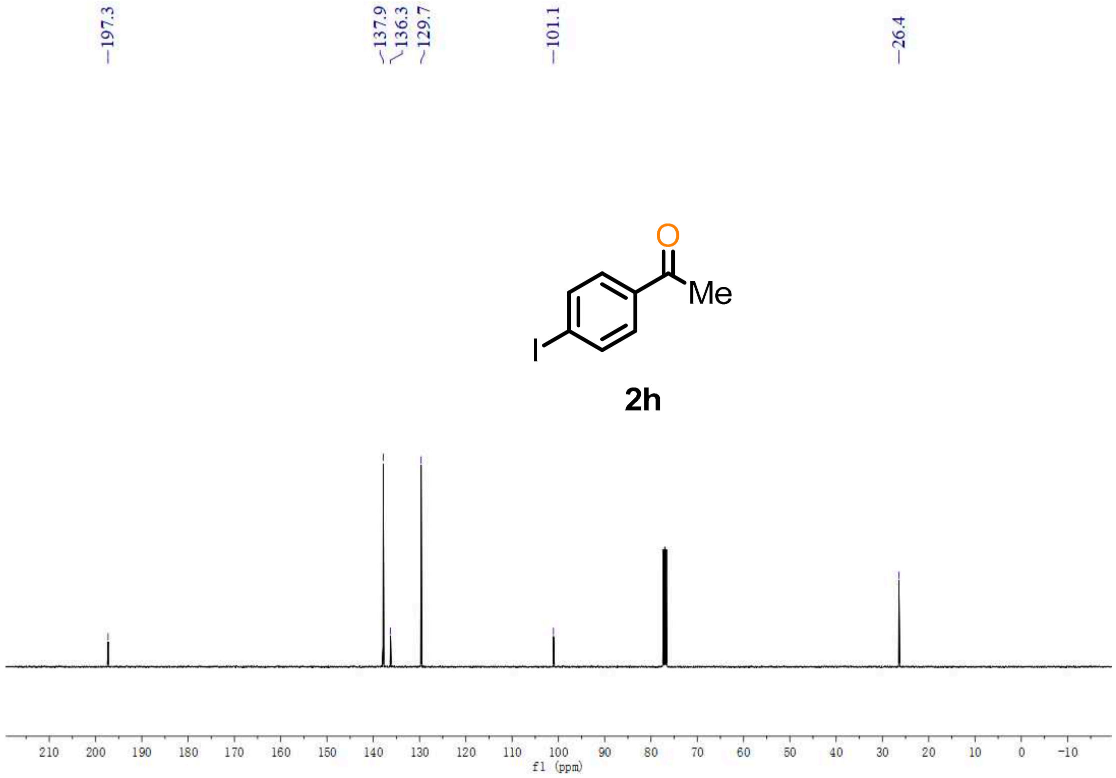

^{1}H NMR spectrum of 1-(4-(trifluoromethyl)phenyl)ethan-1-one (2i, 400 MHz, $CDCl_3$)

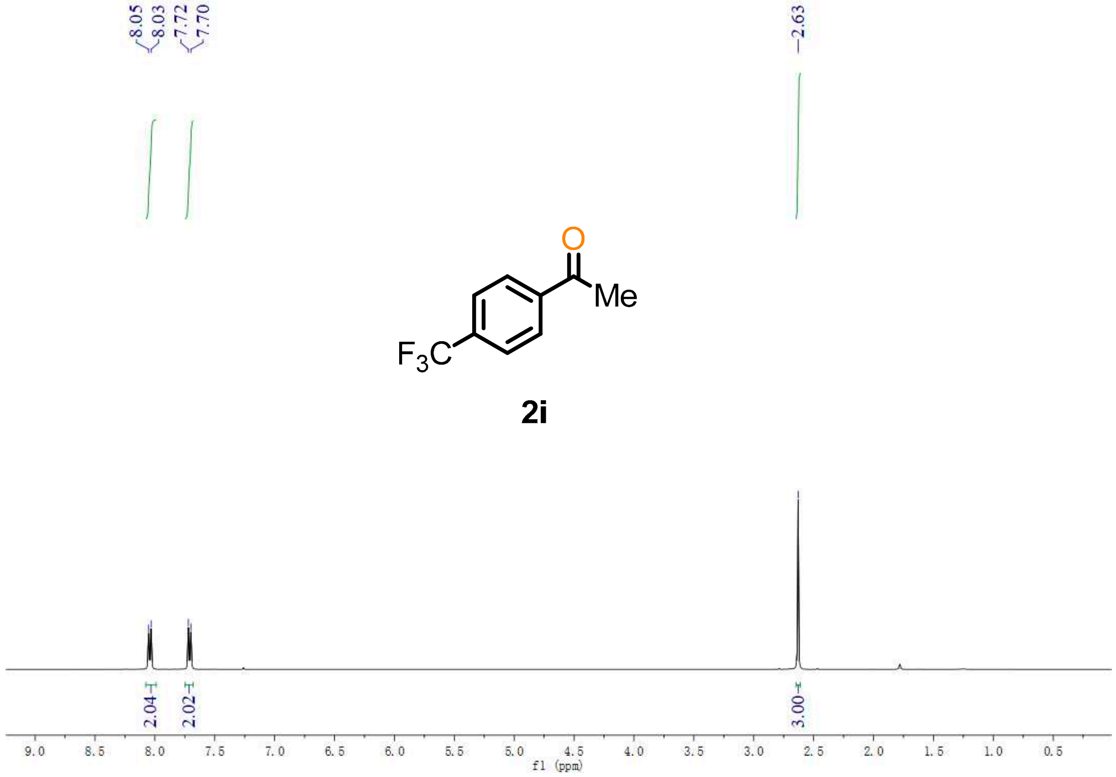

^{13}C NMR spectrum of 1-(4-(trifluoromethyl)phenyl)ethan-1-one (2i, 100 MHz, $CDCl_3$)

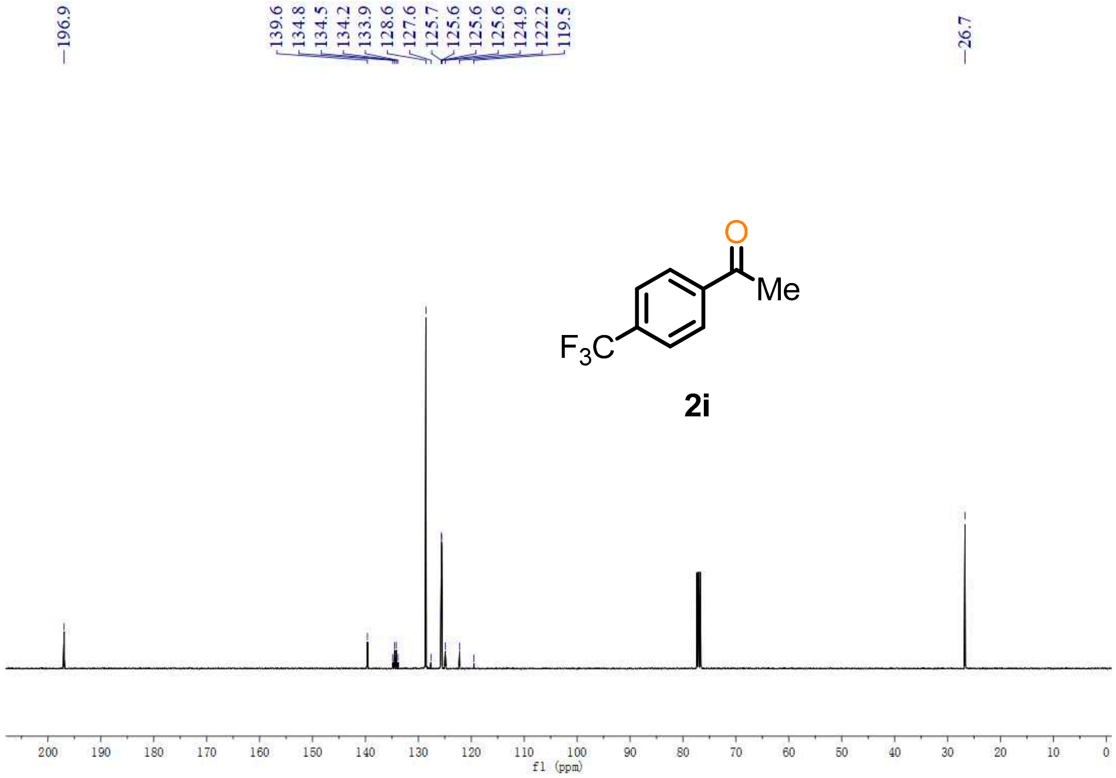

^{19}F NMR spectrum of 1-(4-(trifluoromethyl)phenyl)ethan-1-one (2i, 376 MHz, $CDCl_3$)

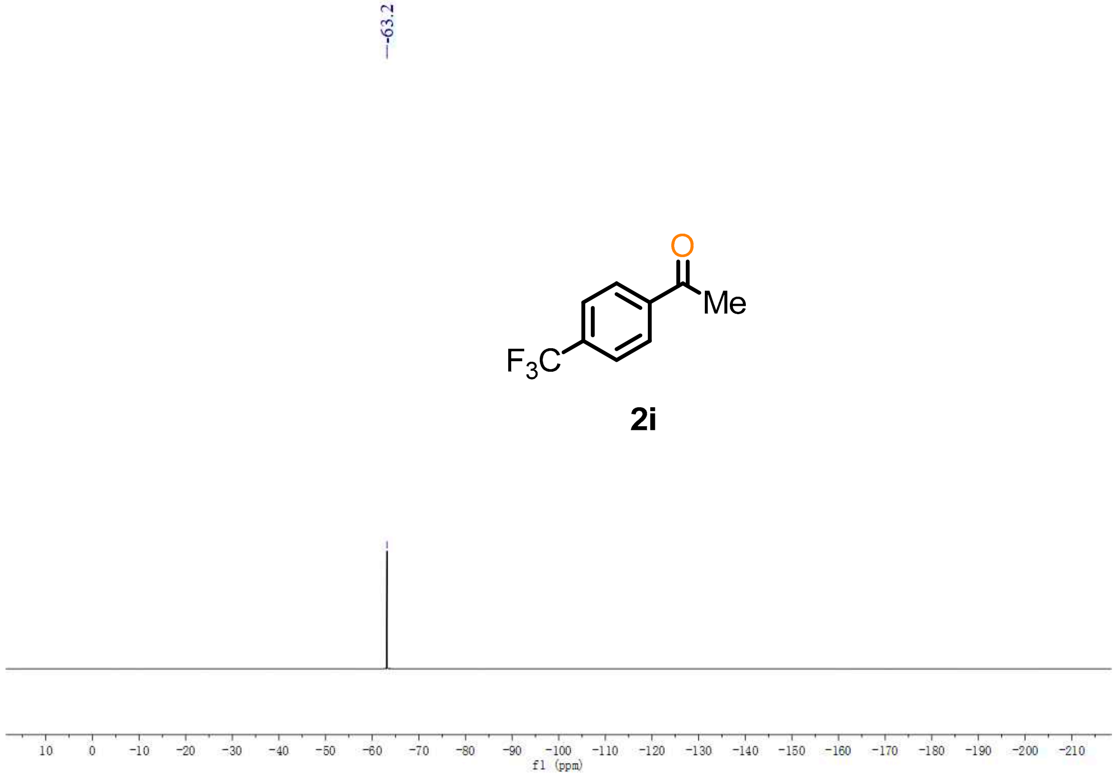

^{1}H NMR spectrum of 4-acetylbenzonitrile (2j, 400 MHz, $CDCl_3$)

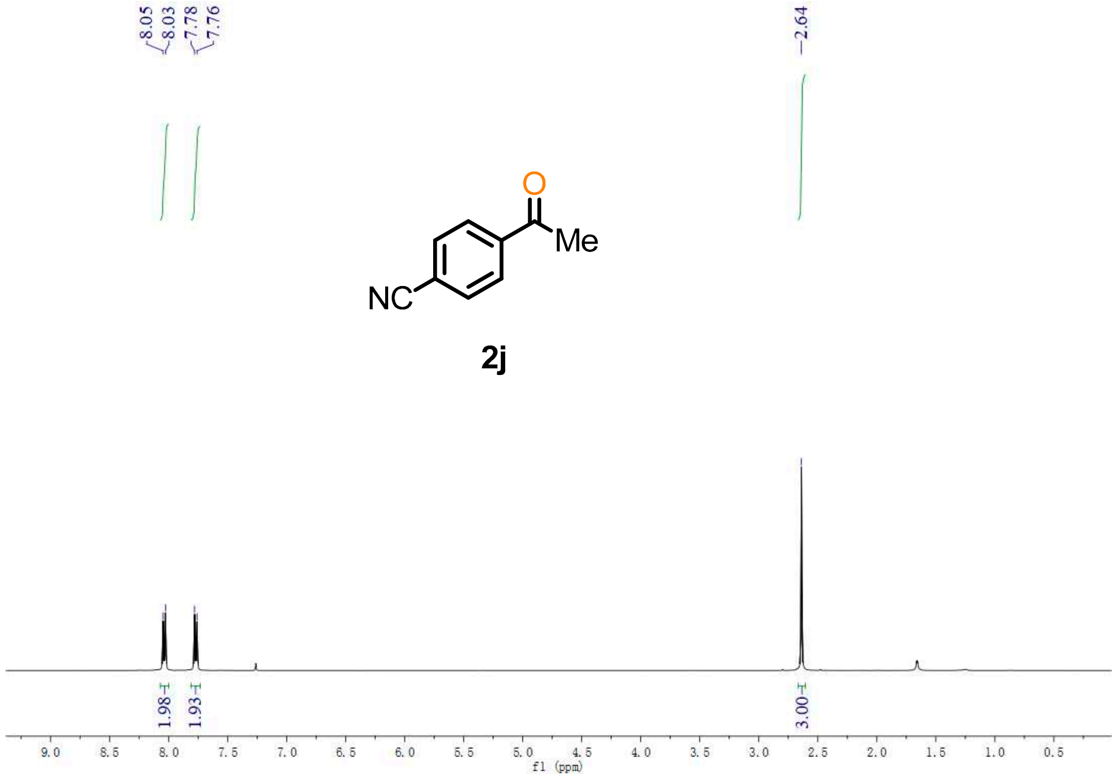

^{13}C NMR spectrum of 4-acetylbenzonitrile (2j, 100 MHz, $CDCl_3$)

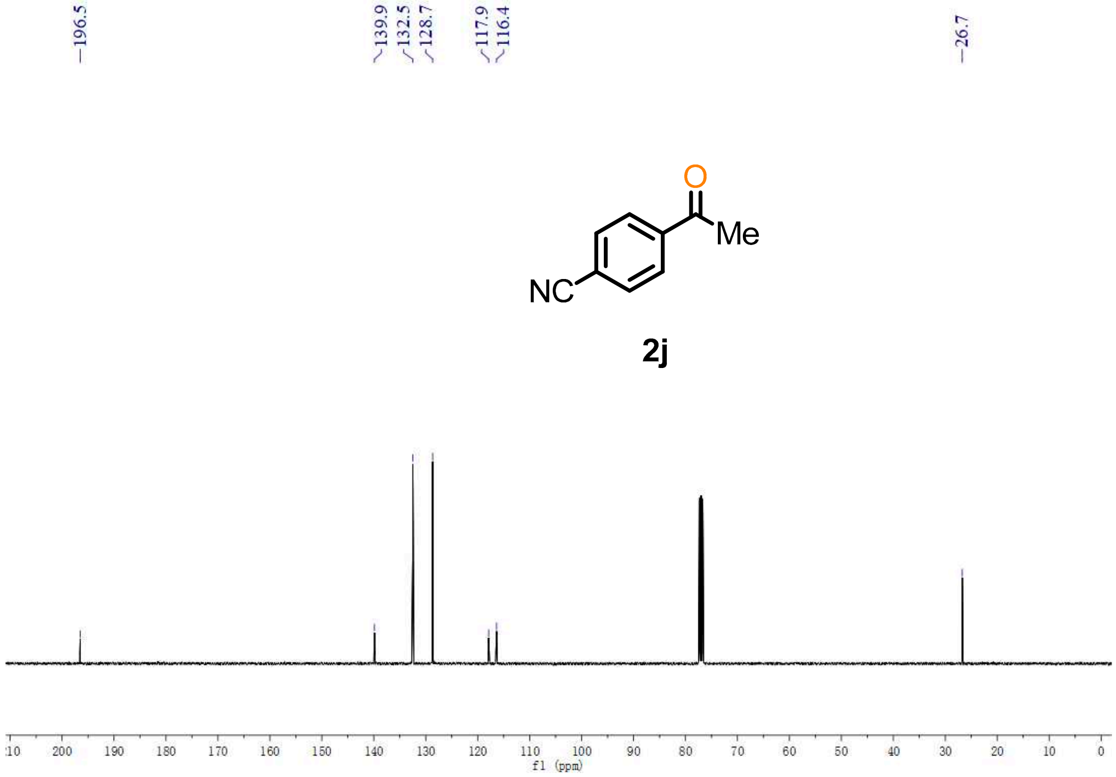

^{1}H NMR spectrum of methyl 4-acetylbenzoate (2k, 400 MHz, $CDCl_3$)

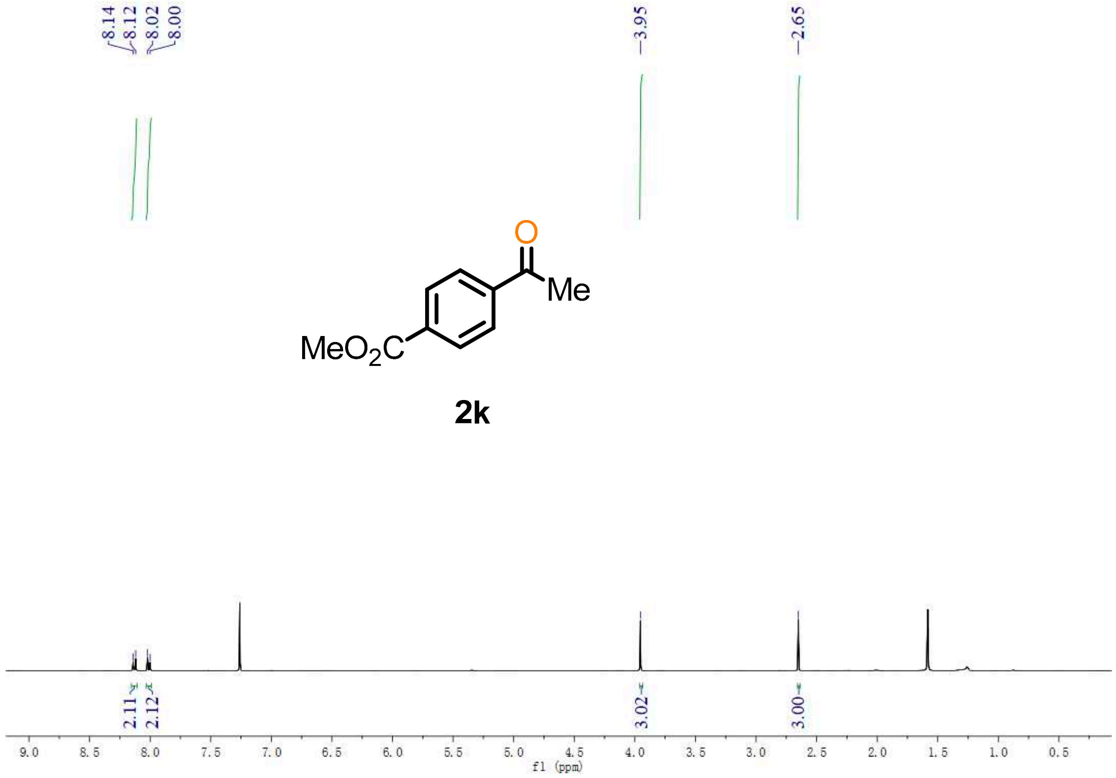

^{13}C NMR spectrum of methyl 4-acetylbenzoate (2k, 100 MHz, $CDCl_3$)

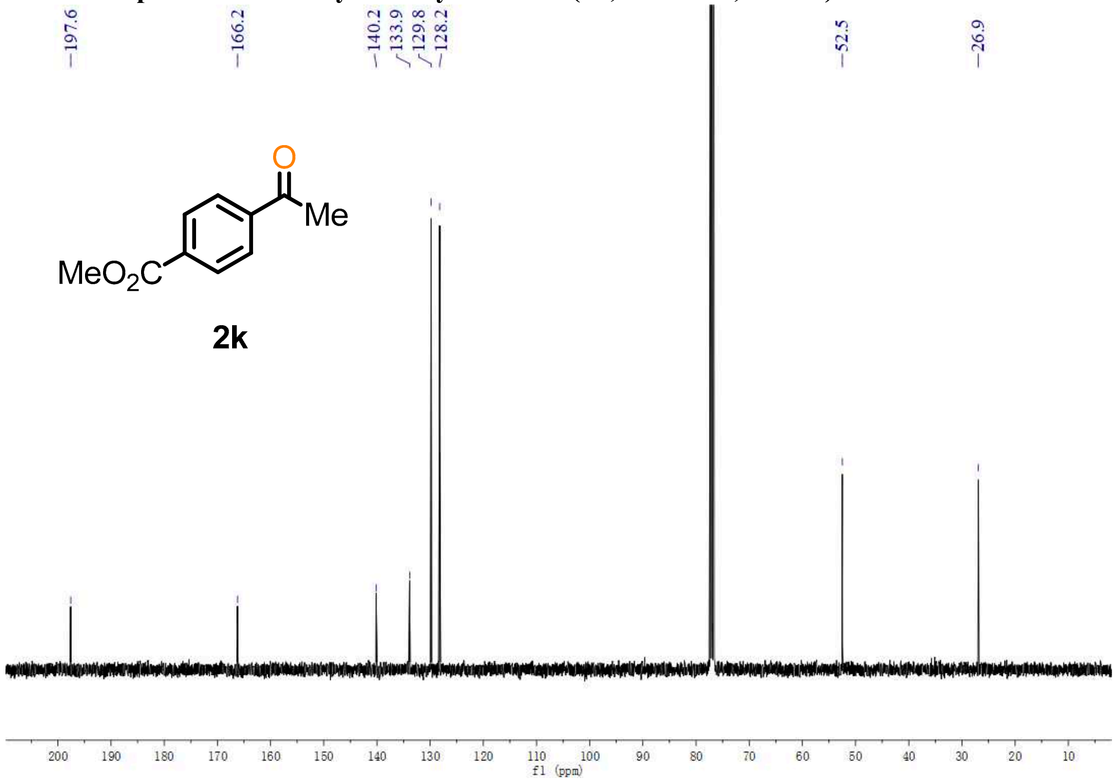

^{1}H NMR spectrum of 1-(*m*-tolyl)ethan-1-one (2l, 400 MHz, CDCl$_3$)

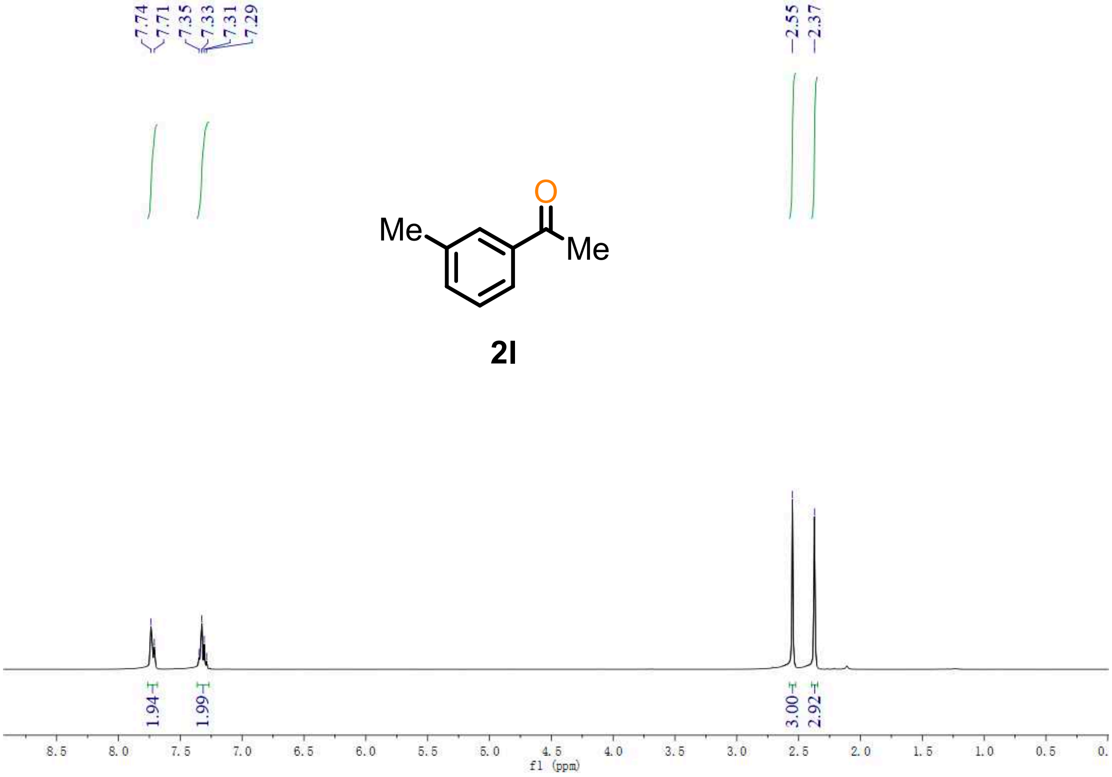

^{13}C NMR spectrum of 1-(*m*-tolyl)ethan-1-one (2l, 100 MHz, CDCl$_3$)

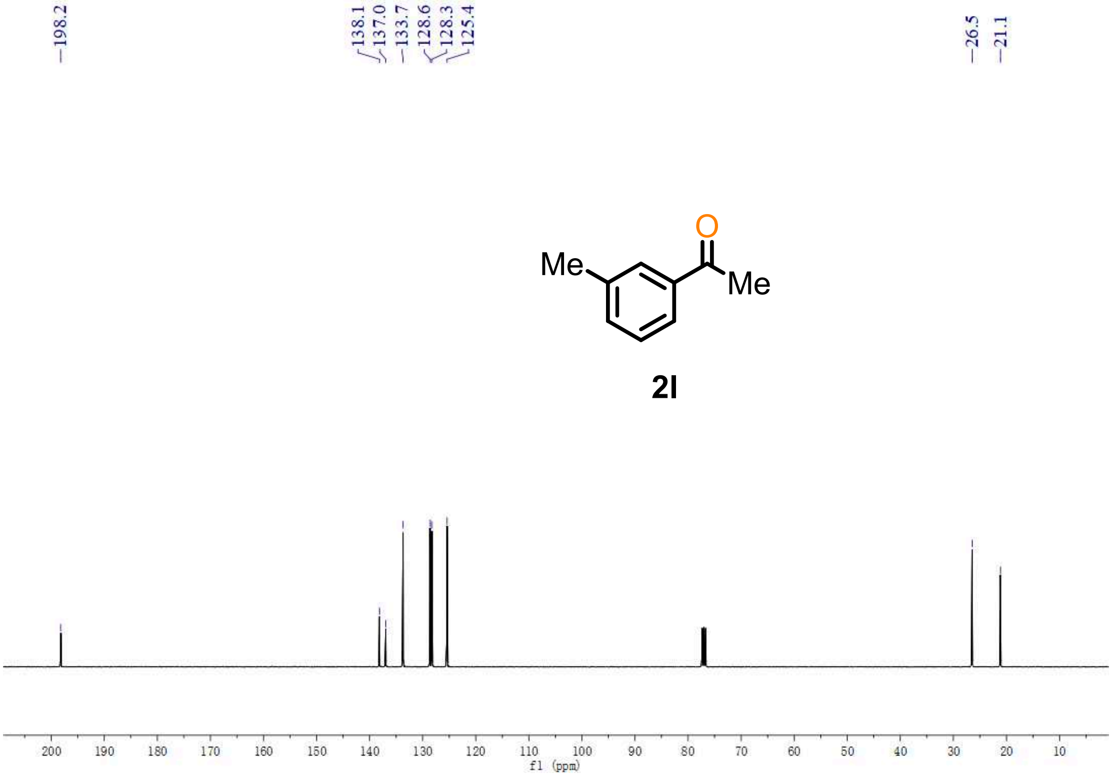

^{1}H NMR spectrum of 1-(*o*-tolyl)ethan-1-one (2m, 400 MHz, $CDCl_3$)

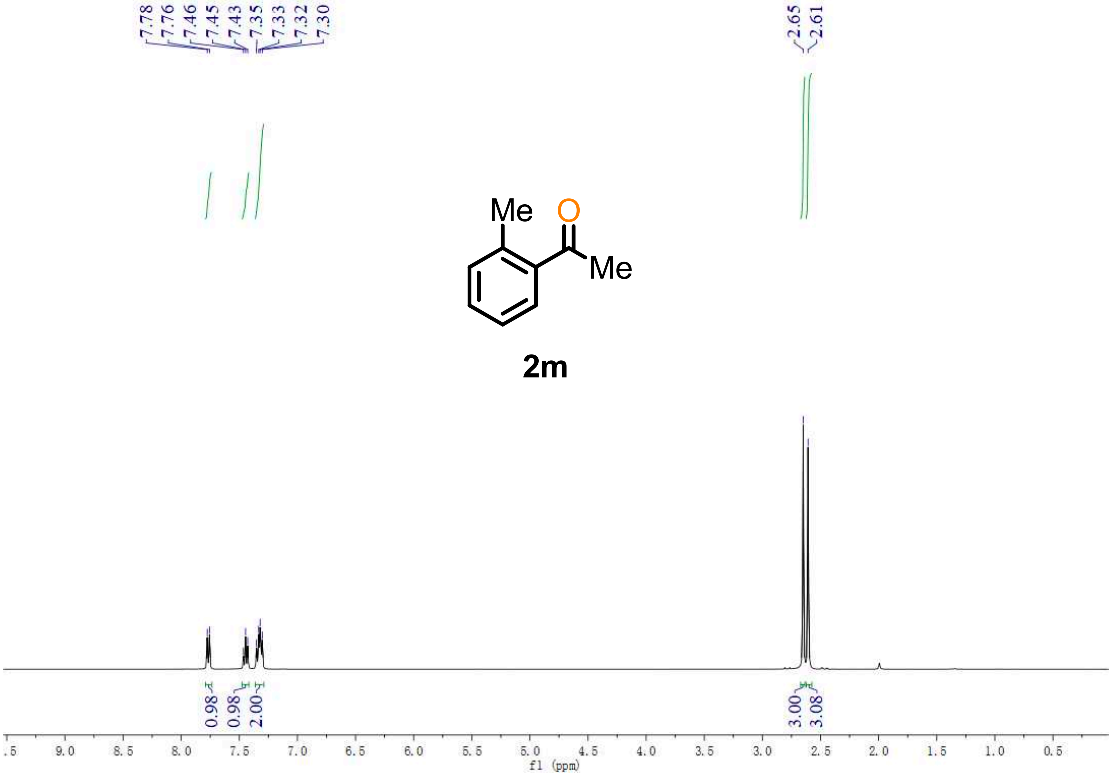

^{13}C NMR spectrum of 1-(*o*-tolyl)ethan-1-one (2m, 100 MHz, $CDCl_3$)

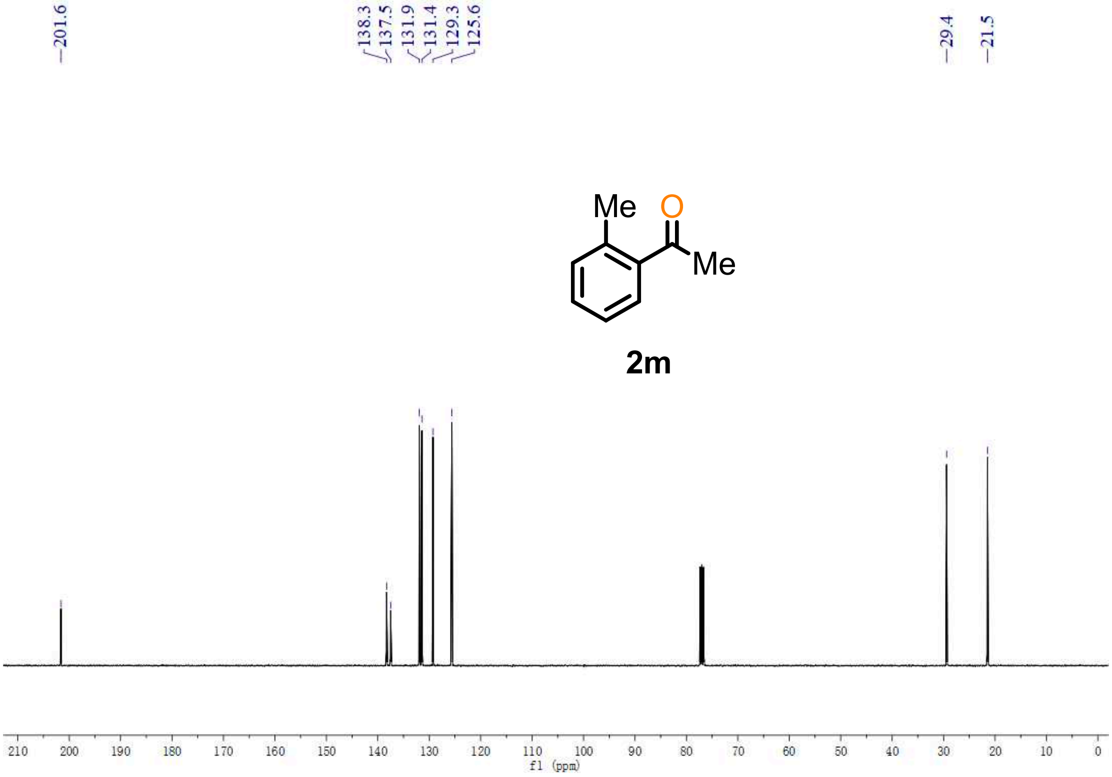

^{1}H NMR spectrum of 1,1'-(1,4-phenylene)bis(ethan-1-one) (2n, 400 MHz, $CDCl_3$)

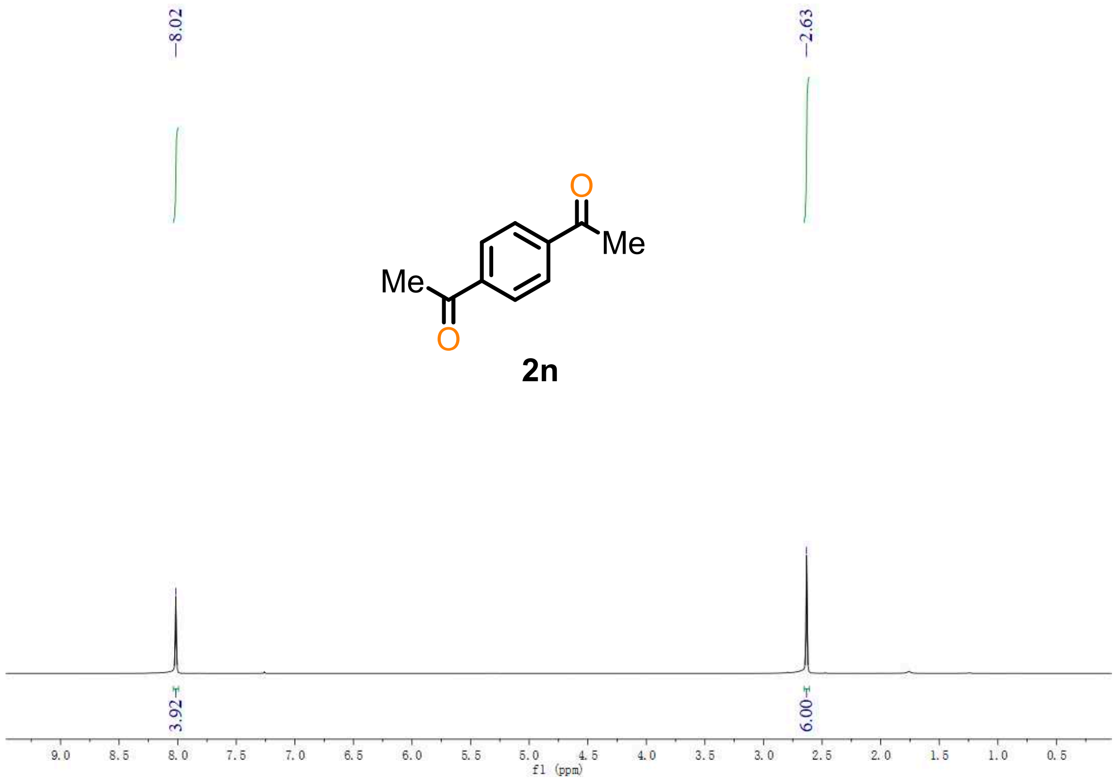

^{13}C NMR spectrum of l 1,1'-(1,4-phenylene)bis(ethan-1-one) (2n, 100 MHz, $CDCl_3$)

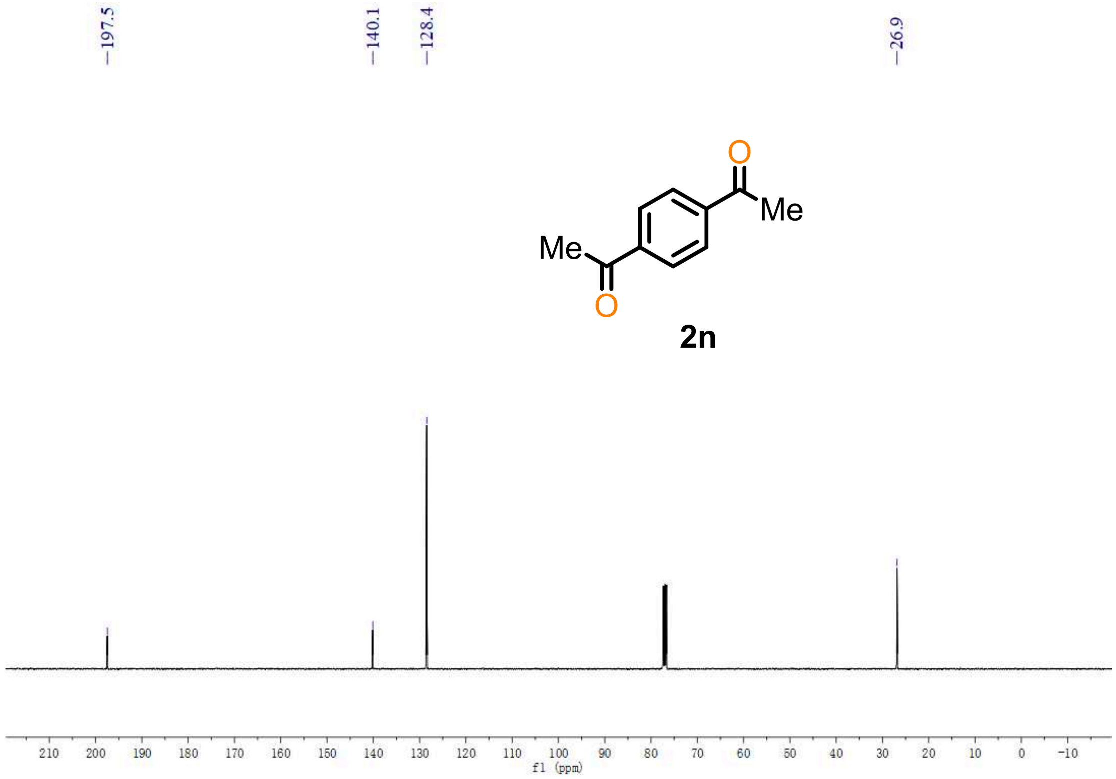

^{1}H NMR spectrum of 1-(3,4-dimethylphenyl)ethan-1-one (2o, 400 MHz, $CDCl_3$)

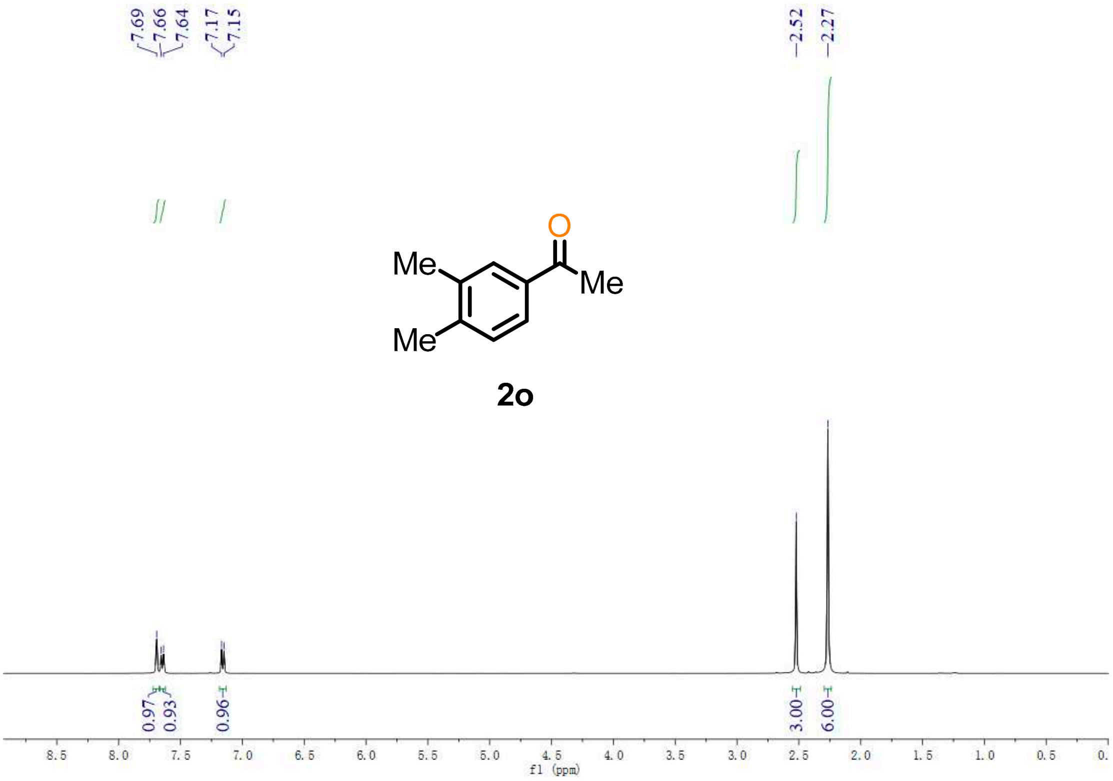

^{13}C NMR spectrum of 1-(3,4-dimethylphenyl)ethan-1-one (2o, 100 MHz, $CDCl_3$)

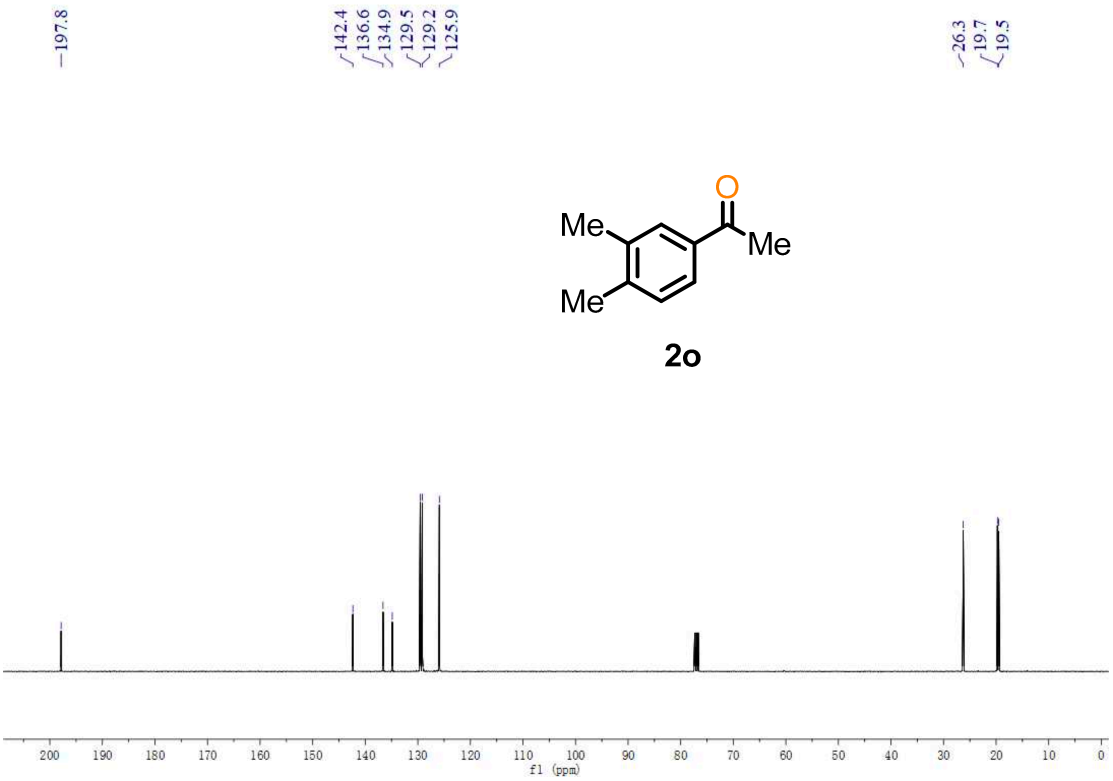

^{1}H NMR spectrum of 1-(3,4-dichlorophenyl)ethan-1-one (2p, 400 MHz, $CDCl_3$)

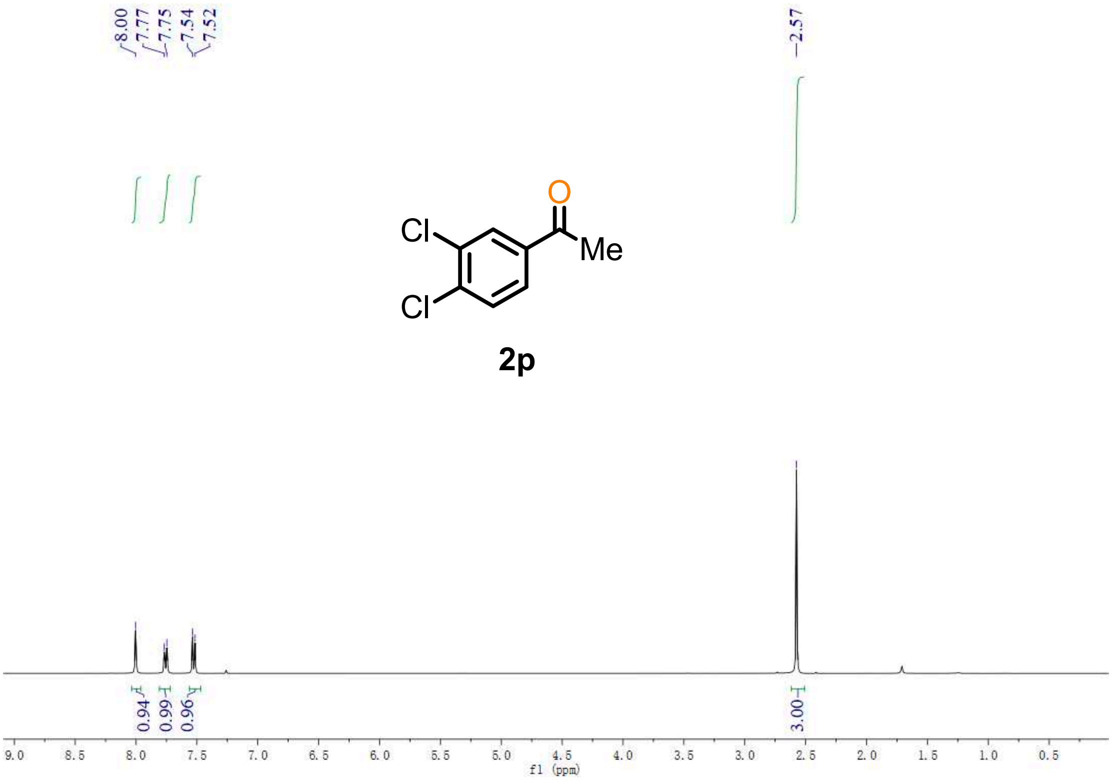

^{13}C NMR spectrum of 1-(3,4-dichlorophenyl)ethan-1-one (2p, 100 MHz, $CDCl_3$)

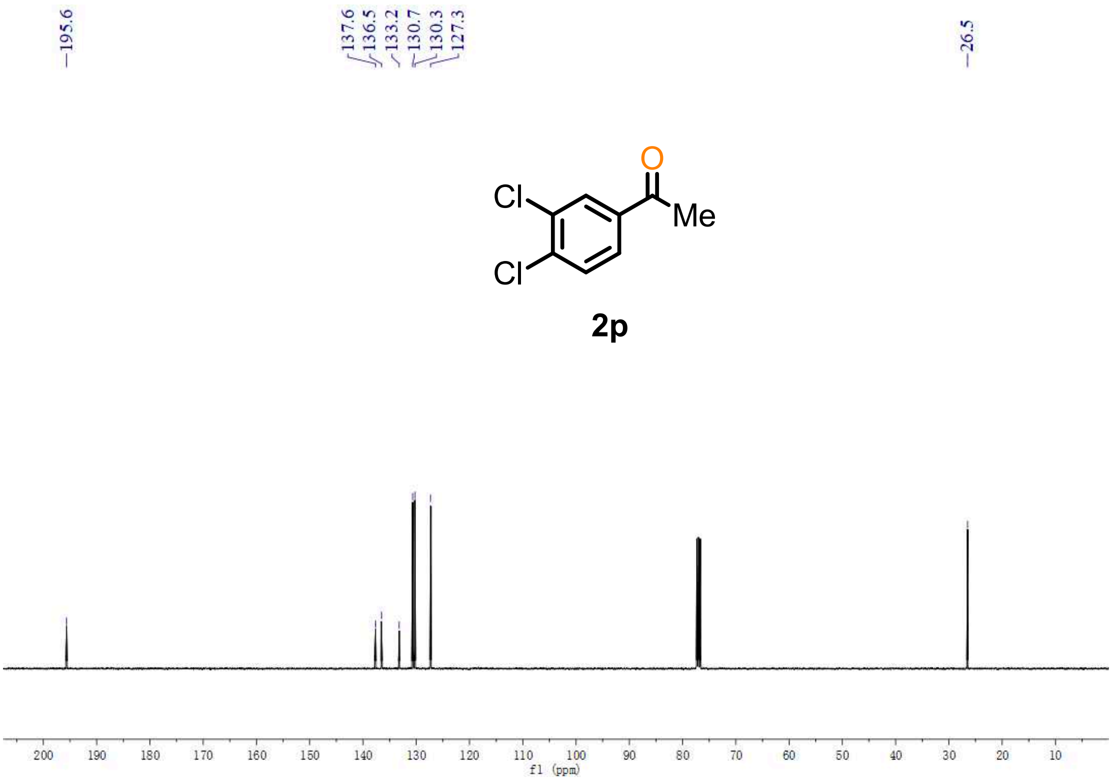

^{1}H NMR spectrum of 2,3-dihydro-1*H*-inden-1-one (2q, 400 MHz, $CDCl_3$)

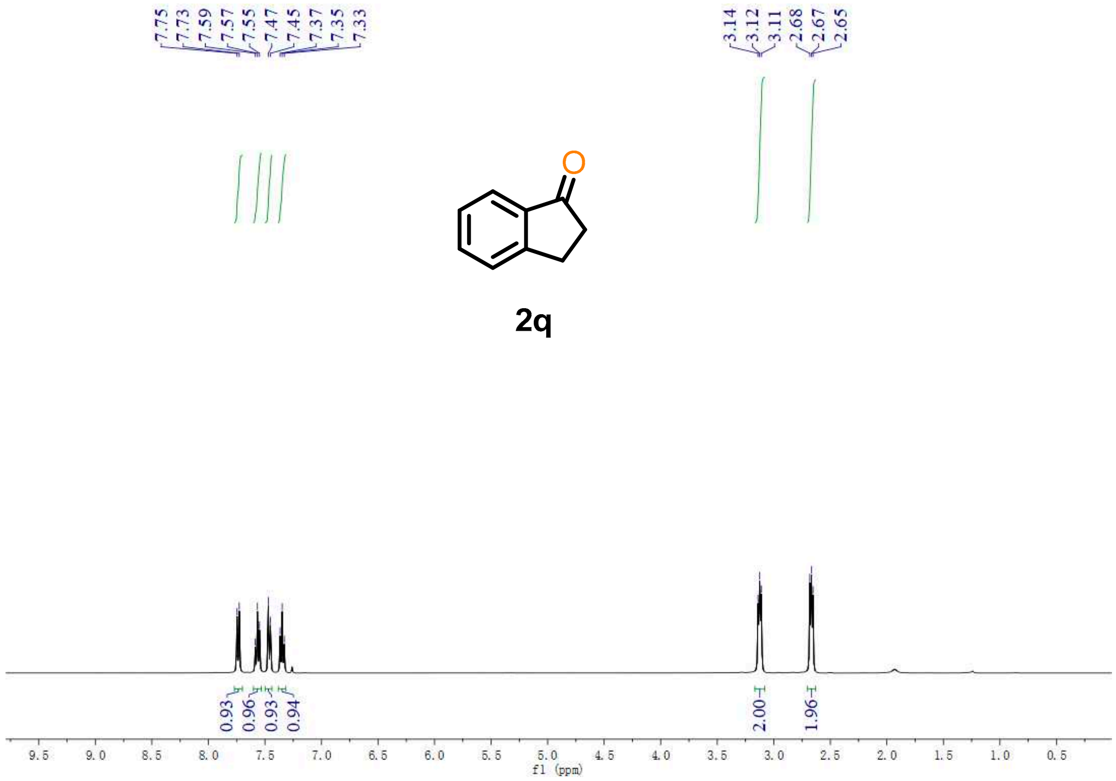

^{13}C NMR spectrum of 2,3-dihydro-1*H*-inden-1-one (2q, 100 MHz, $CDCl_3$)

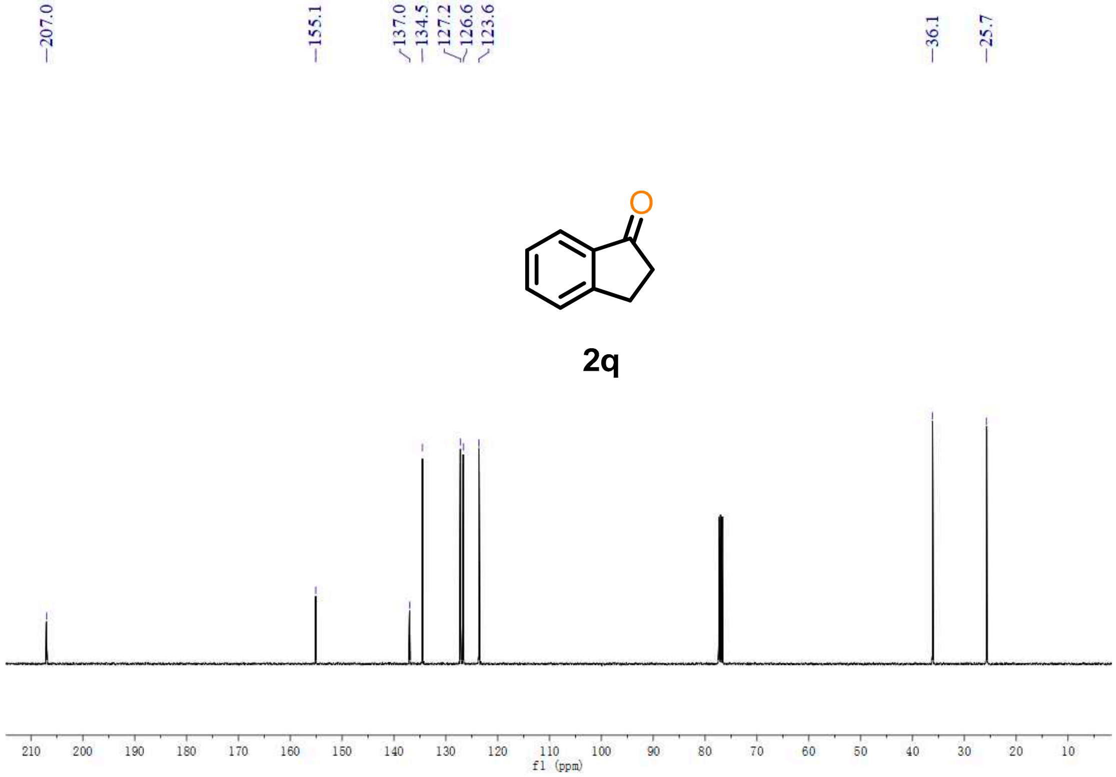

^{1}H NMR spectrum of 9*H*-fluoren-9-one (2r, 400 MHz, $CDCl_3$)

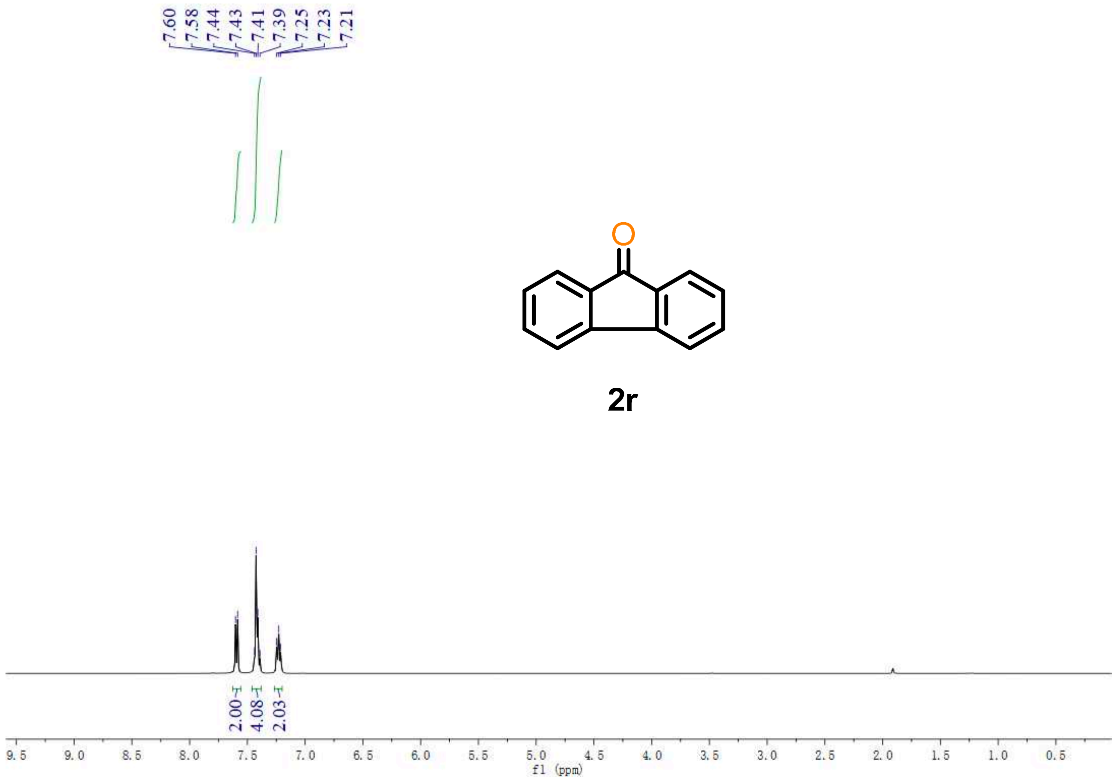

^{13}C NMR spectrum of 9*H*-fluoren-9-one (2r, 100 MHz, $CDCl_3$)

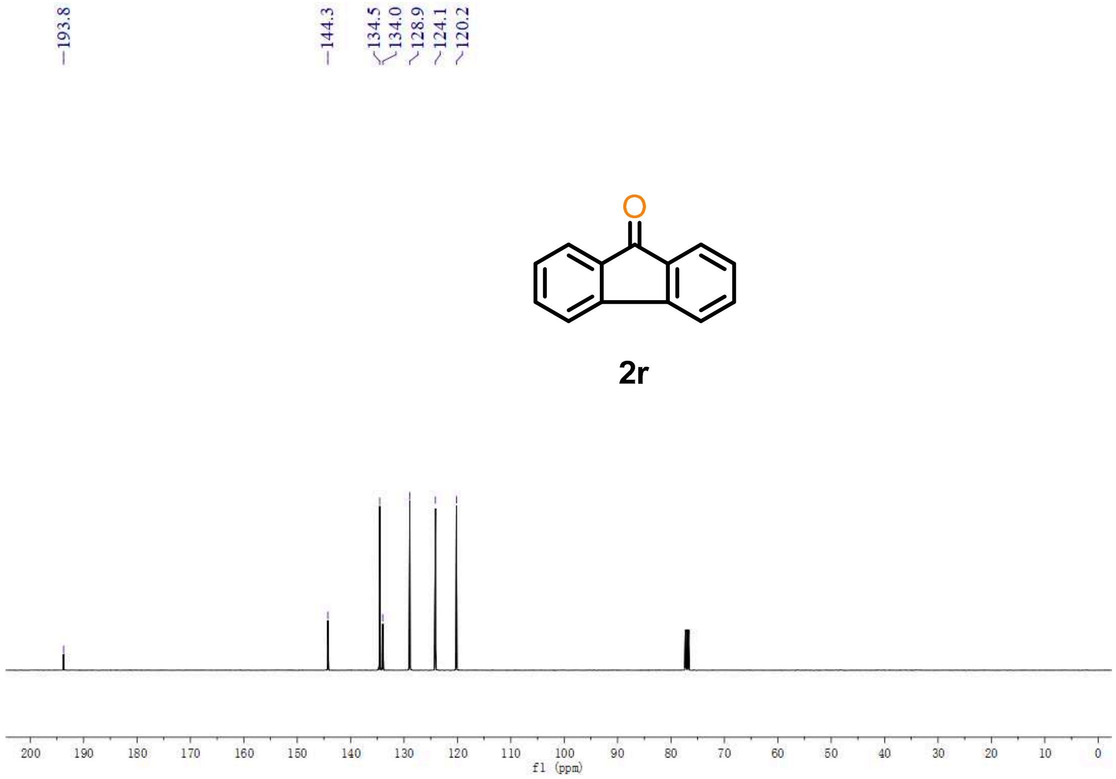

^{1}H NMR spectrum of 2-amino-9*H*-fluoren-9-one (2s, 400 MHz, DMSO-*d6*)

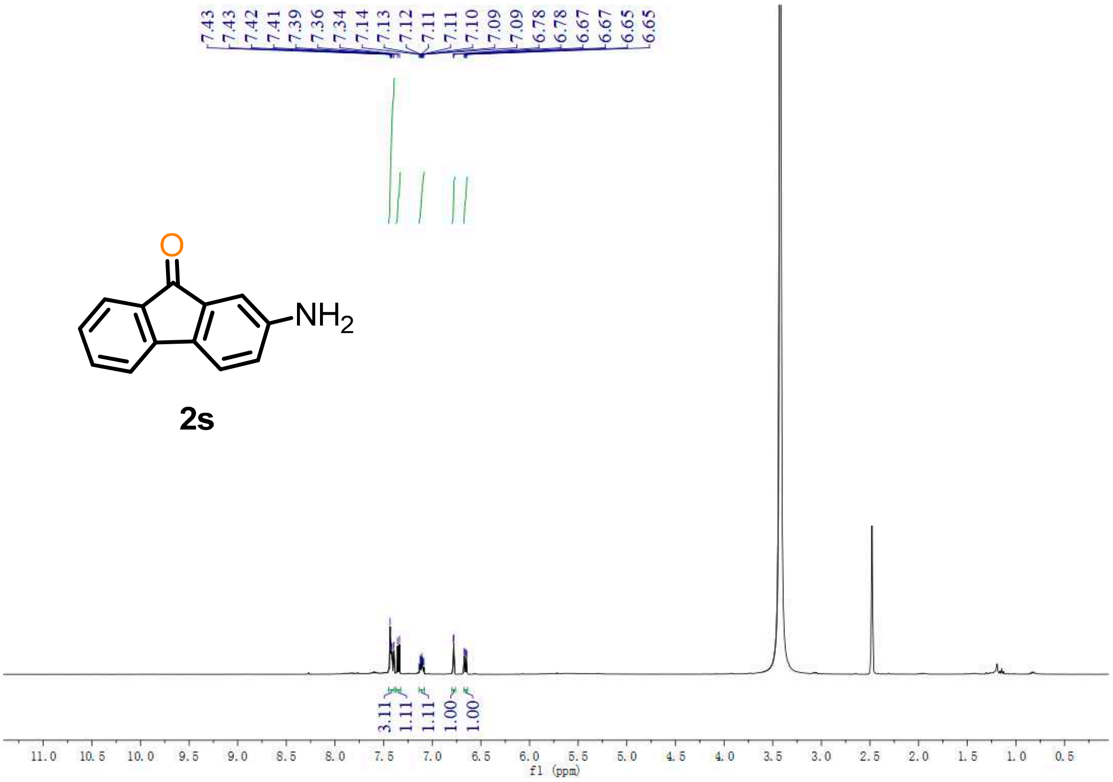

^{13}C NMR spectrum of 2-amino-9*H*-fluoren-9-one (2s, 100 MHz, DMSO-*d6*)

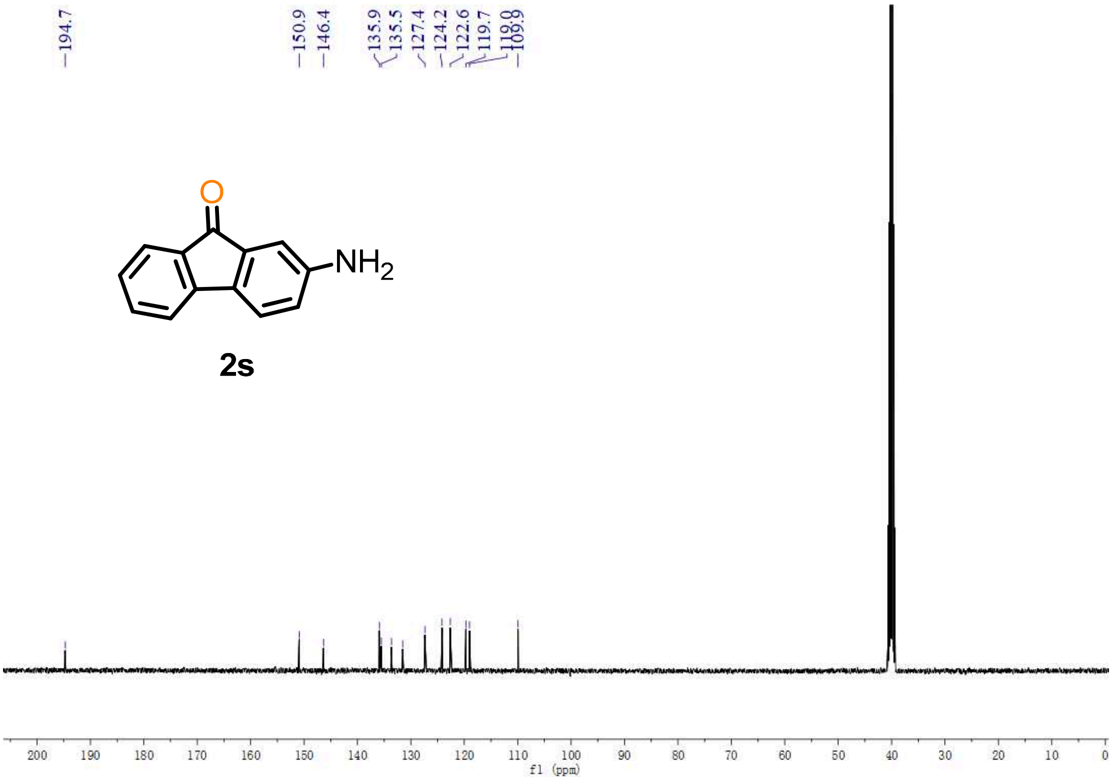

^{1}H NMR spectrum of 9*H*-xanthen-9-one (2t, 400 MHz, $CDCl_3$)

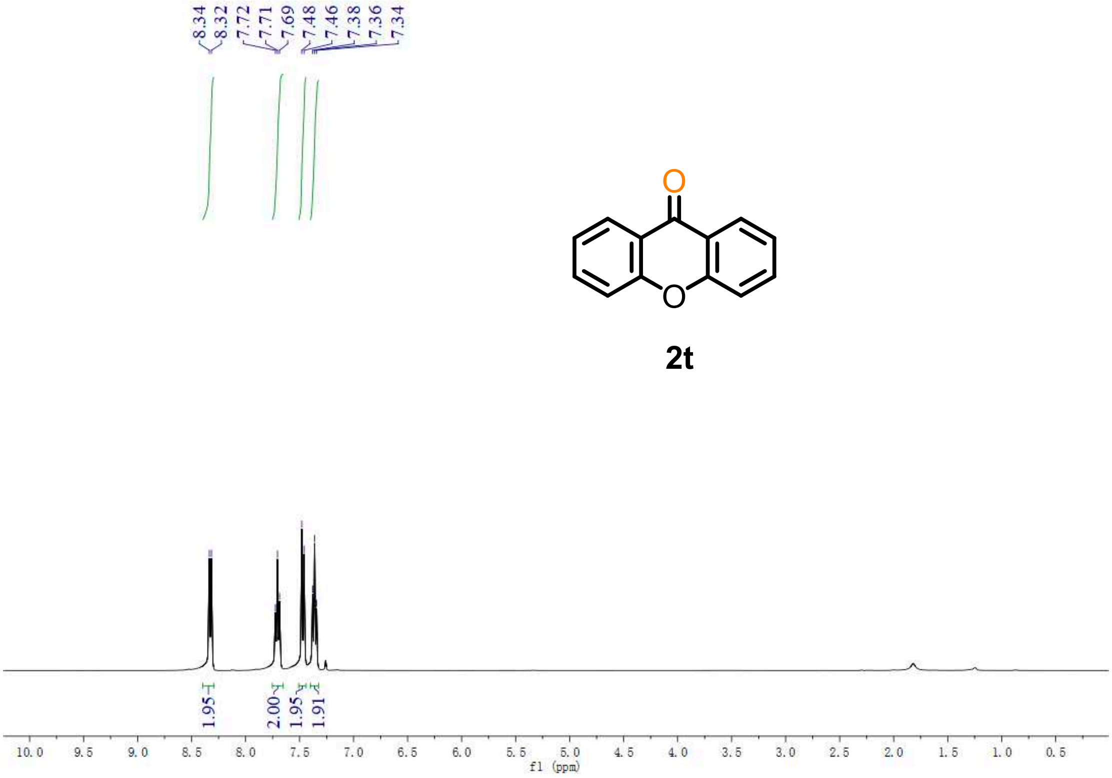

^{13}C NMR spectrum of 9*H*-xanthen-9-one (2t, 100 MHz, $CDCl_3$)

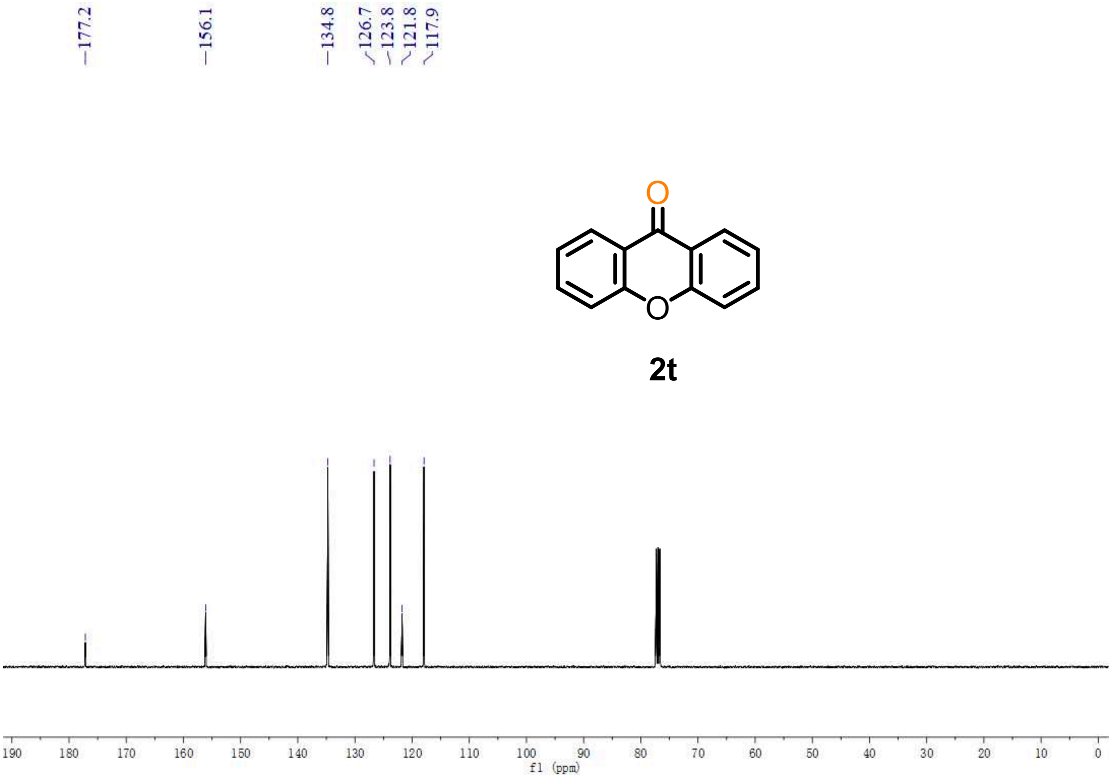

^{1}H NMR spectrum of 1-(naphthalen-1-yl)ethan-1-one (2u, 400 MHz, $CDCl_3$)

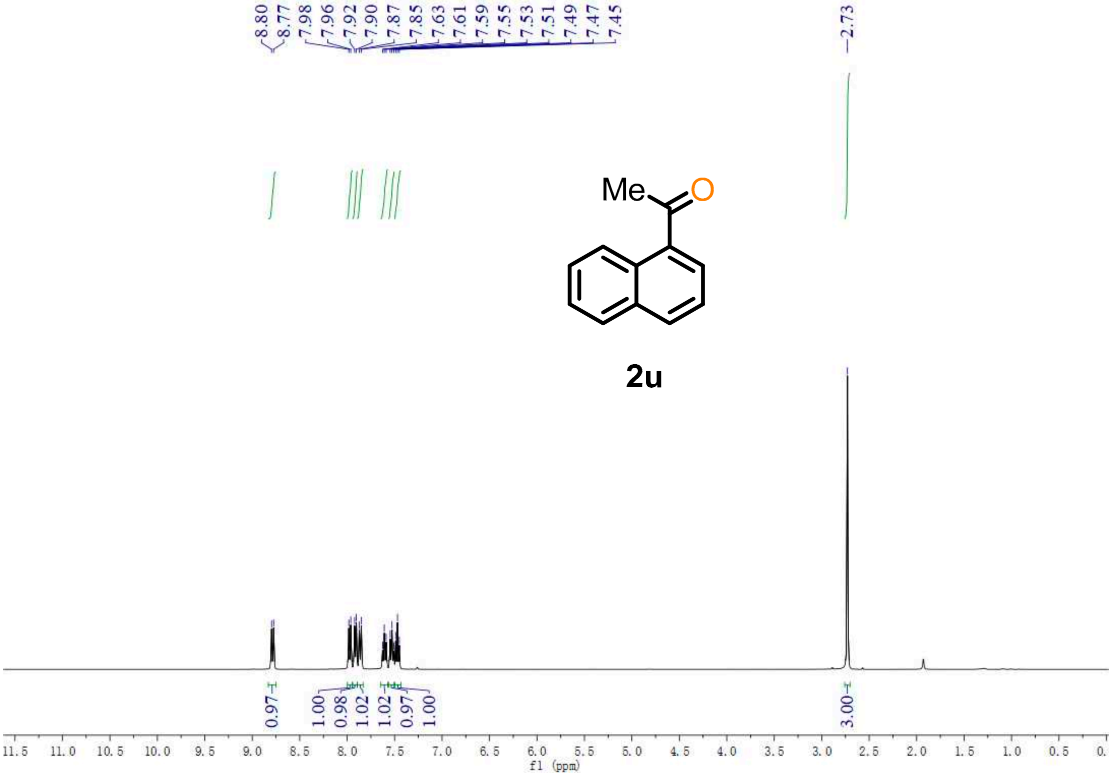

^{13}C NMR spectrum of 1-(naphthalen-1-yl)ethan-1-one (2u, 100 MHz, $CDCl_3$)

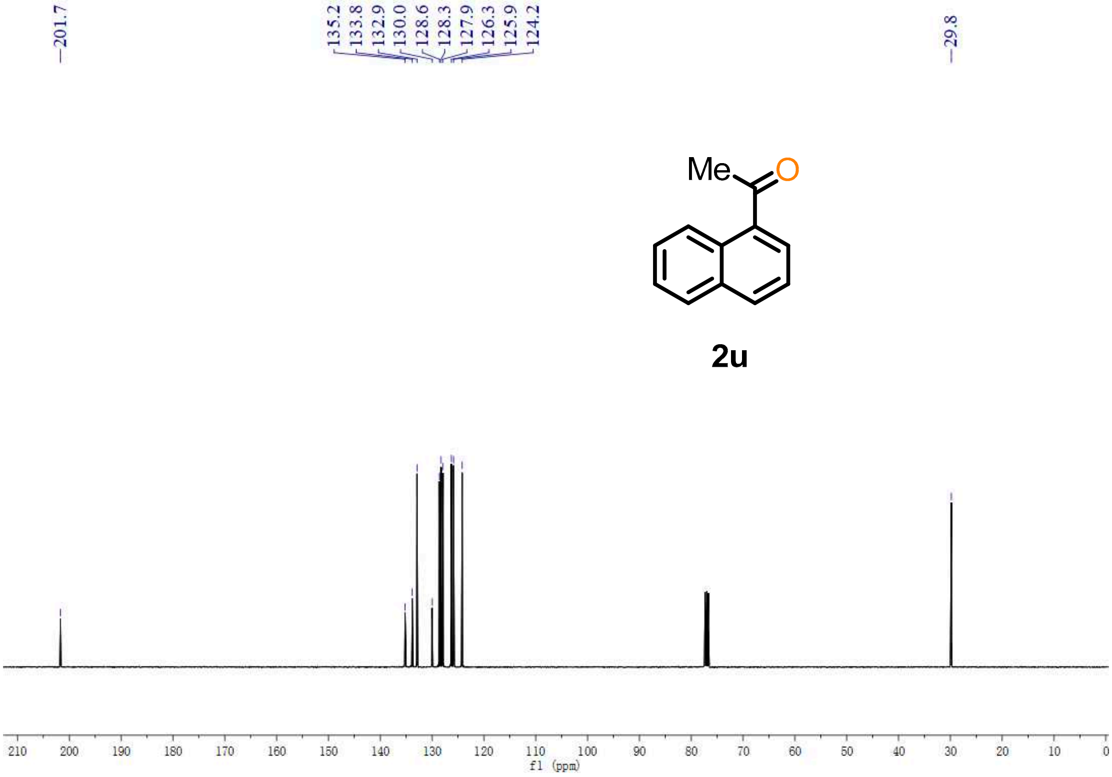

^1H NMR spectrum of 1-(naphthalen-2-yl)ethan-1-one (2v, 400 MHz, $CDCl_3$)

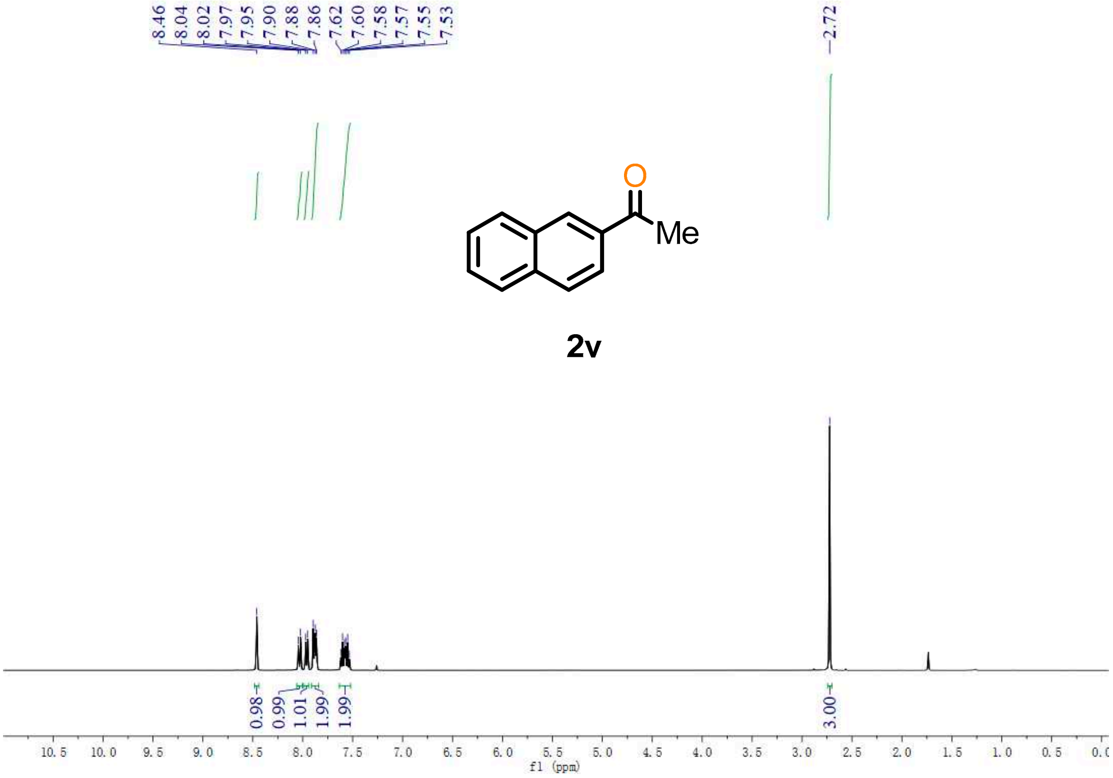

^{13}C NMR spectrum of 1-(naphthalen-2-yl)ethan-1-one (2v, 100 MHz, $CDCl_3$)

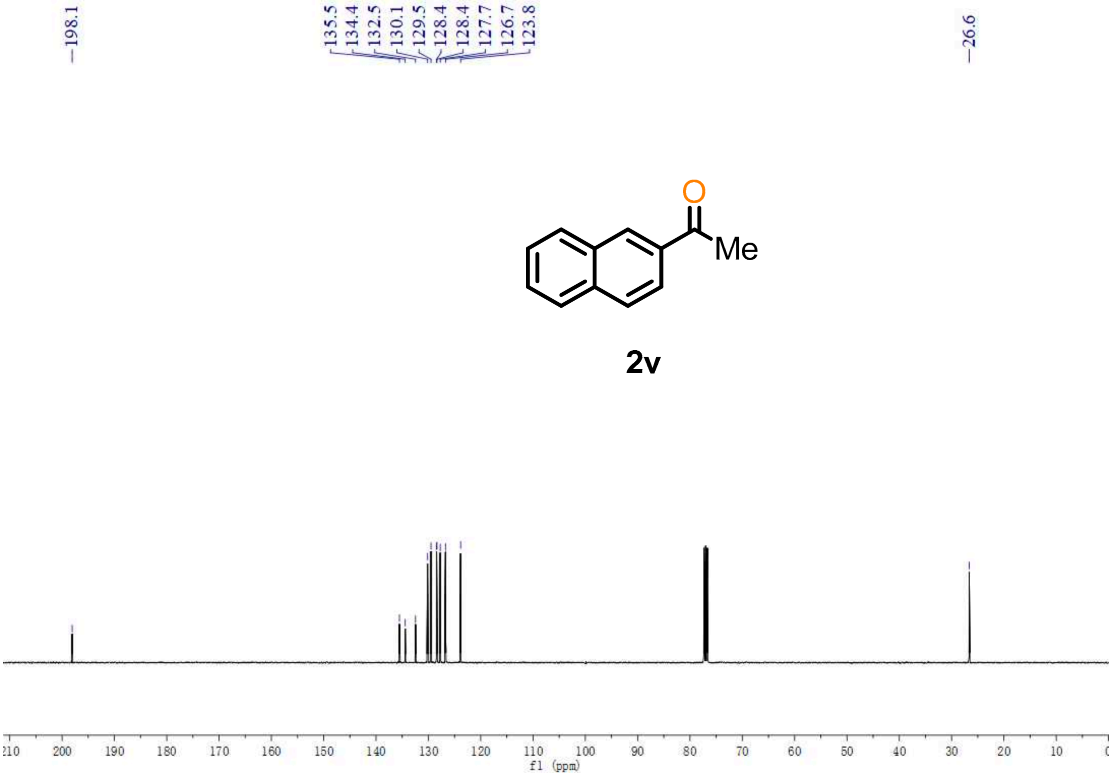

^{1}H NMR spectrum of 1-(pyridin-4-yl)ethan-1-one (2w, 400 MHz, $CDCl_3$)

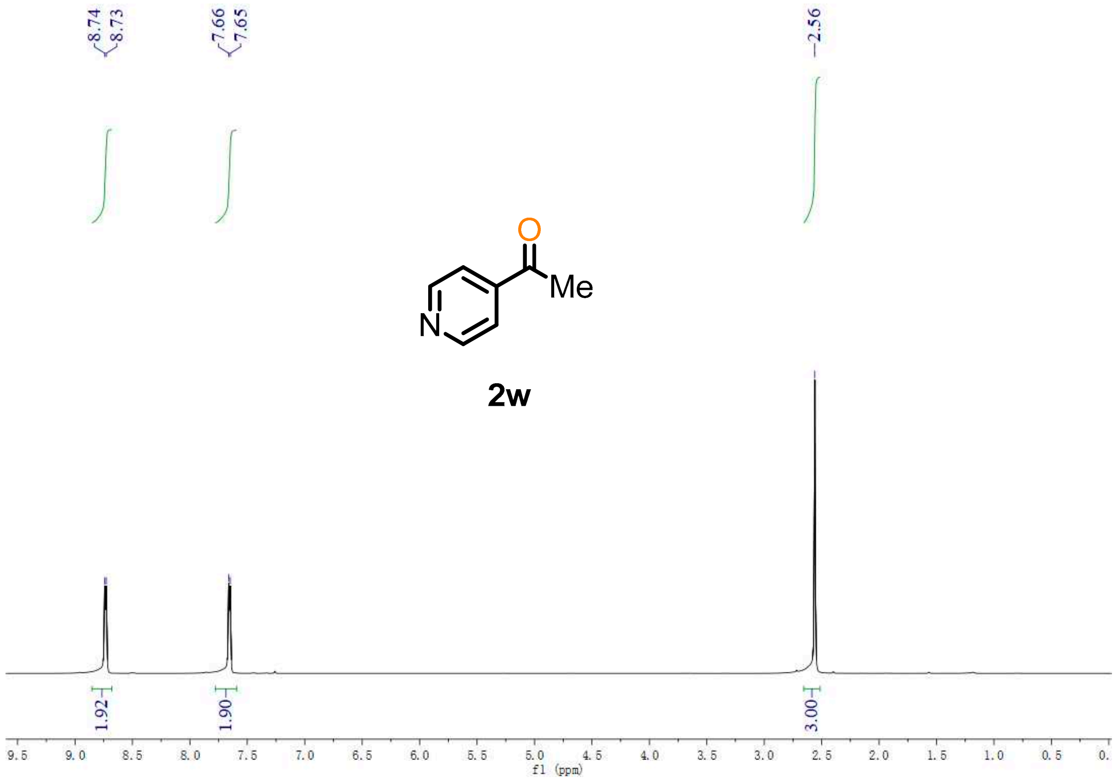

^{13}C NMR spectrum of 1-(pyridin-4-yl)ethan-1-one (2w, 100 MHz, $CDCl_3$)

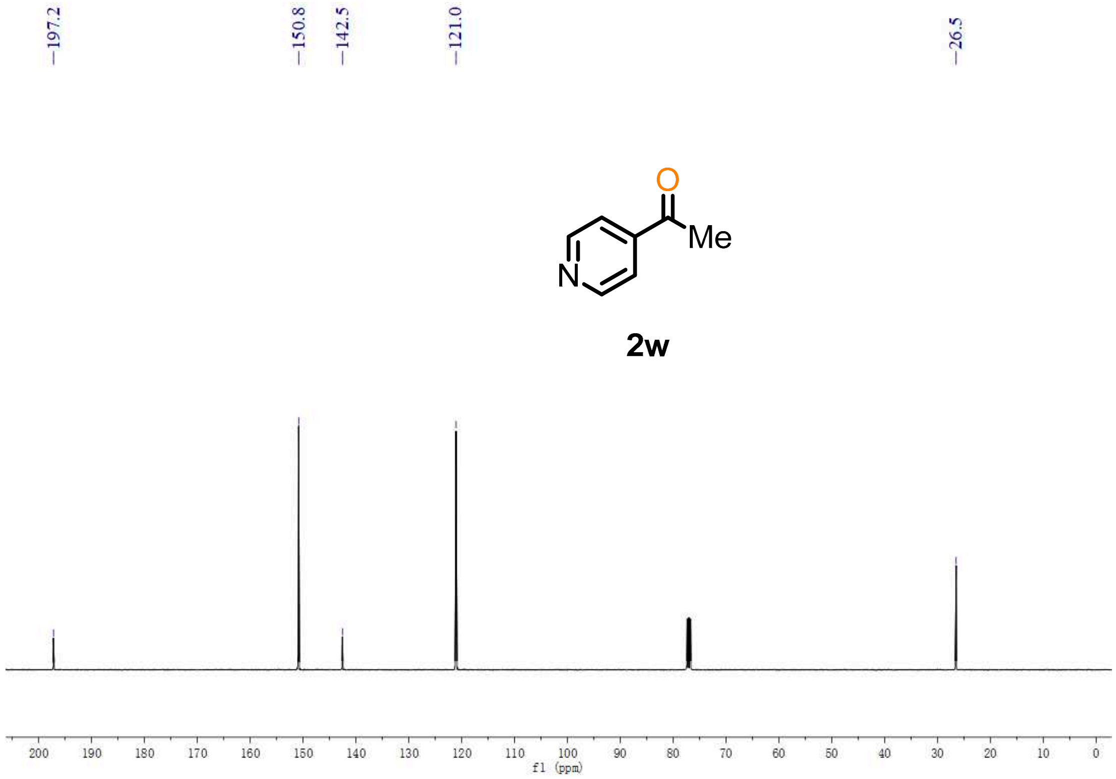

^{1}H NMR spectrum of 1-(thiophen-2-yl)ethan-1-one (2x, 400 MHz, $CDCl_3$)

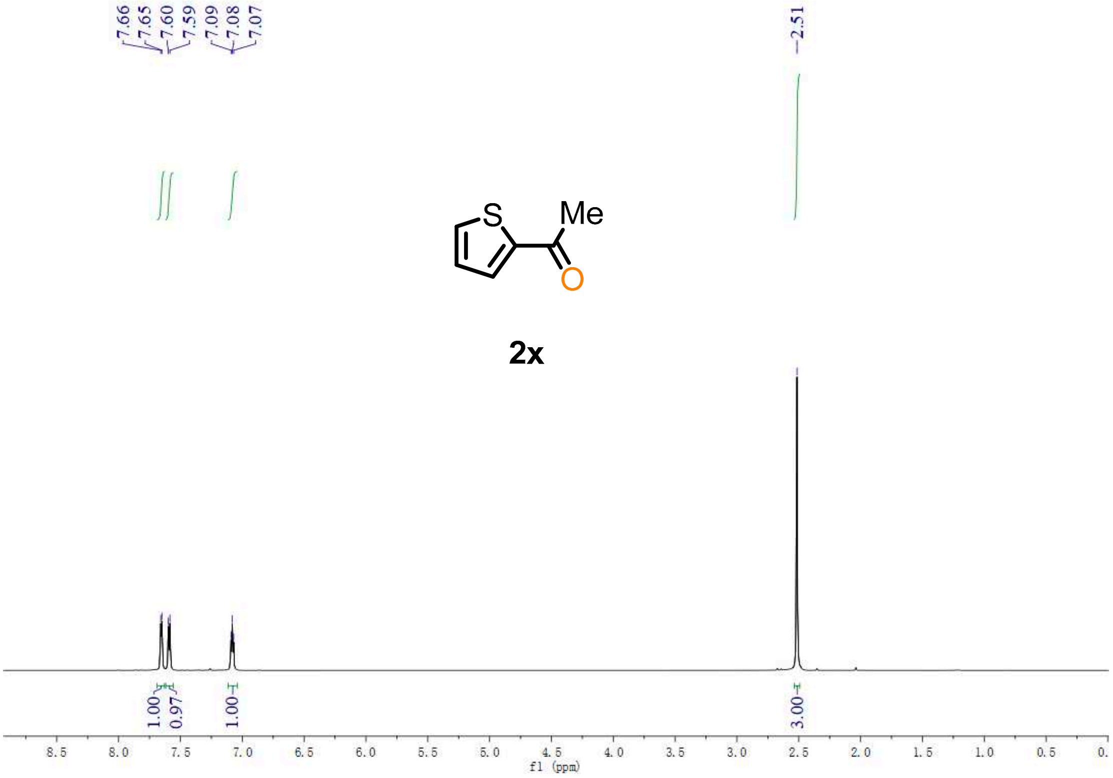

^{13}CNMR spectrum of 1-(thiophen-2-yl)ethan-1-one (2x, 100 MHz, $CDCl_3$)

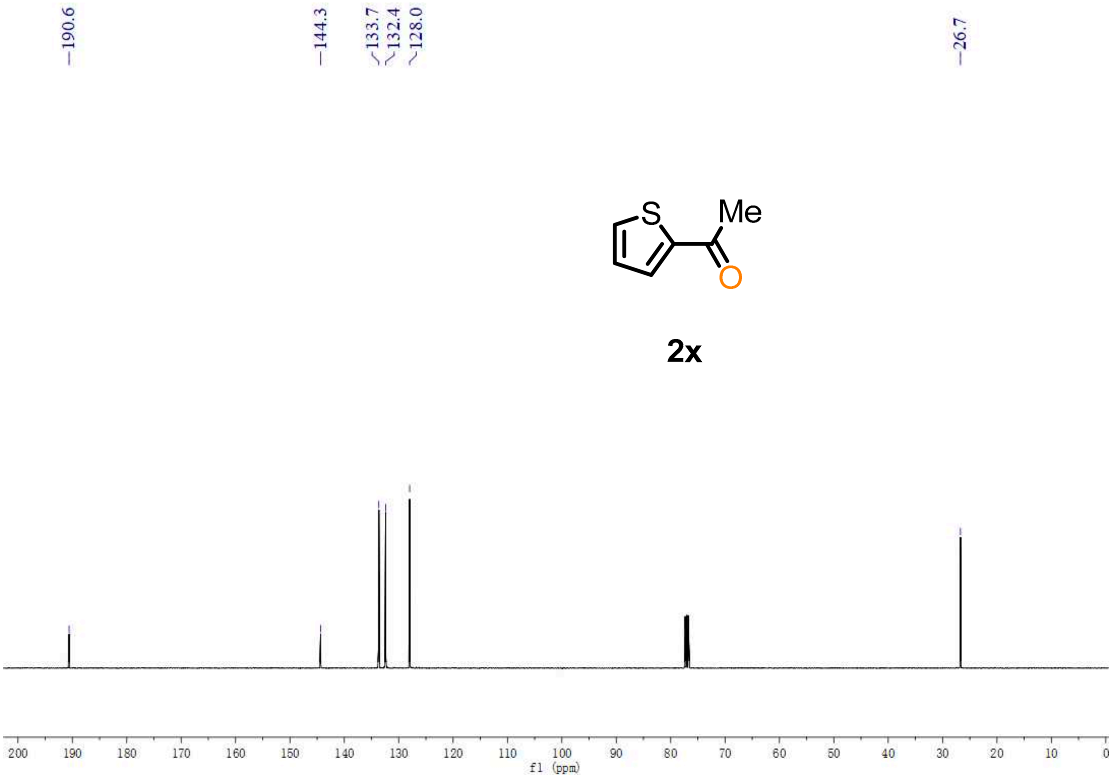

^{1}H NMR spectrum of 1-(benzo[*b*]thiophen-5-yl)ethan-1-one (2y, 400 MHz, $CDCl_3$)

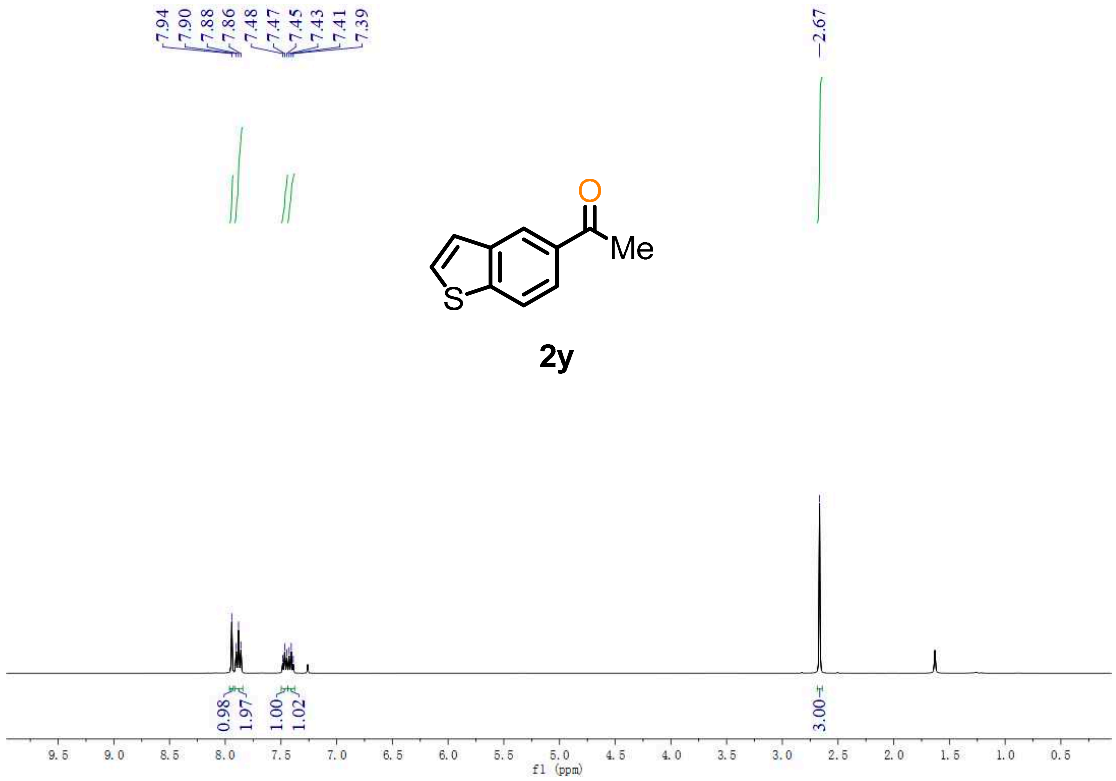

^{13}C NMR spectrum of 1-(benzo[*b*]thiophen-5-yl)ethan-1-one (2y, 100 MHz, $CDCl_3$)

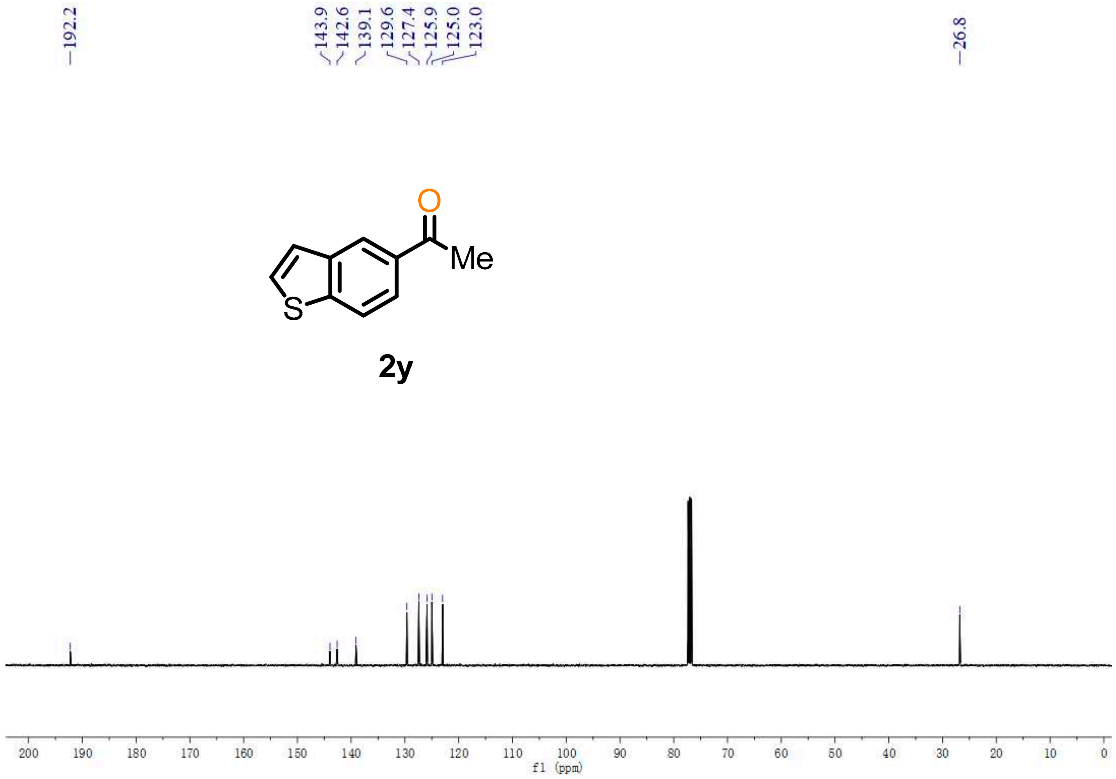

^{1}H NMR spectrum of propiophenone (2z, 400 MHz, $CDCl_3$)

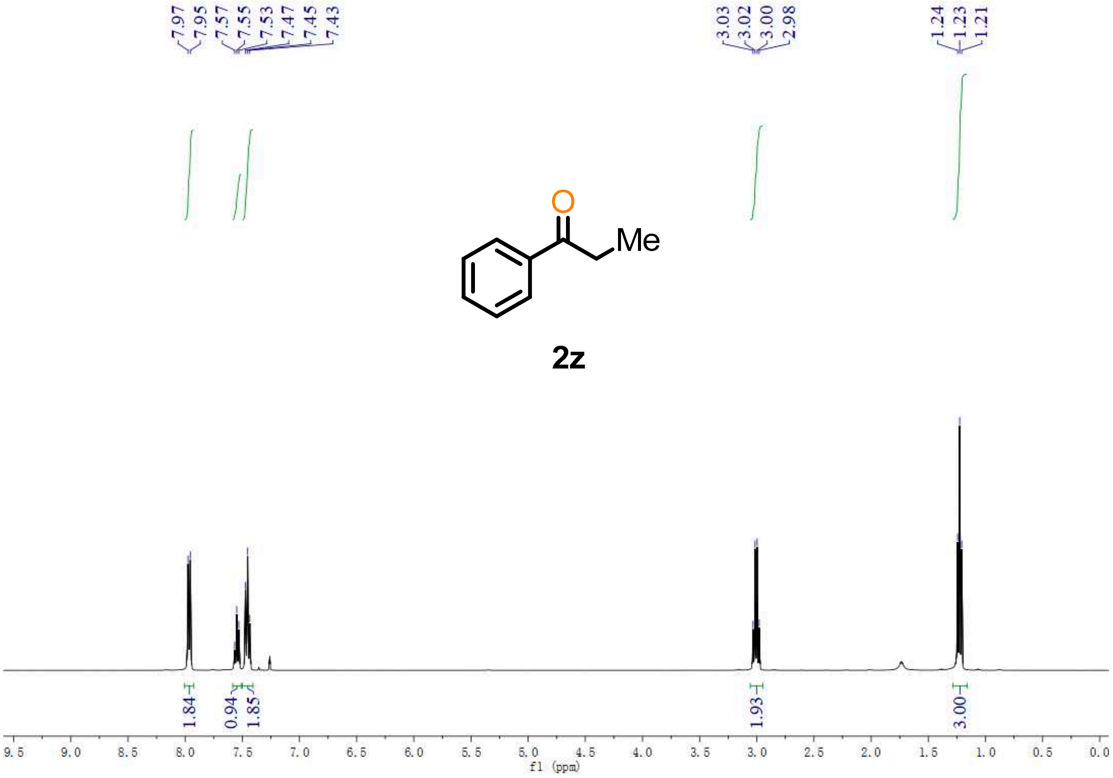

^{13}C NMR spectrum of propiophenone (2z, 100 MHz, $CDCl_3$)

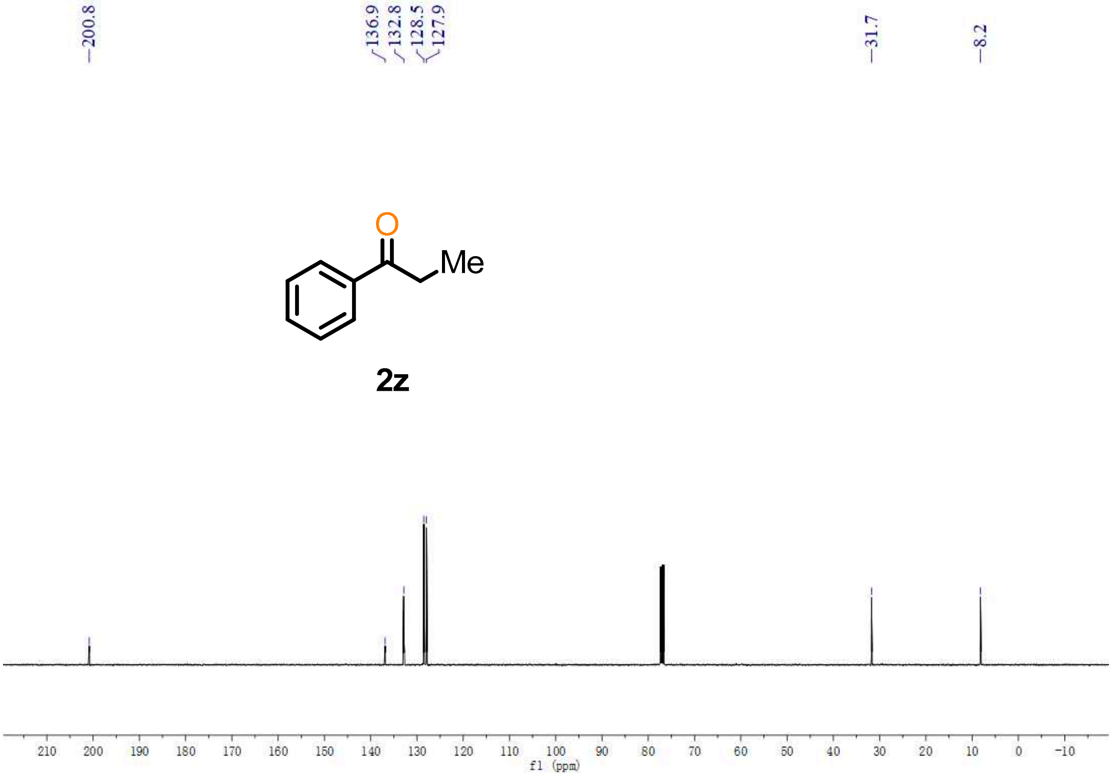

^{1}H NMR spectrum of 1-phenylbutan-1-one (2aa, 400 MHz, $CDCl_3$)

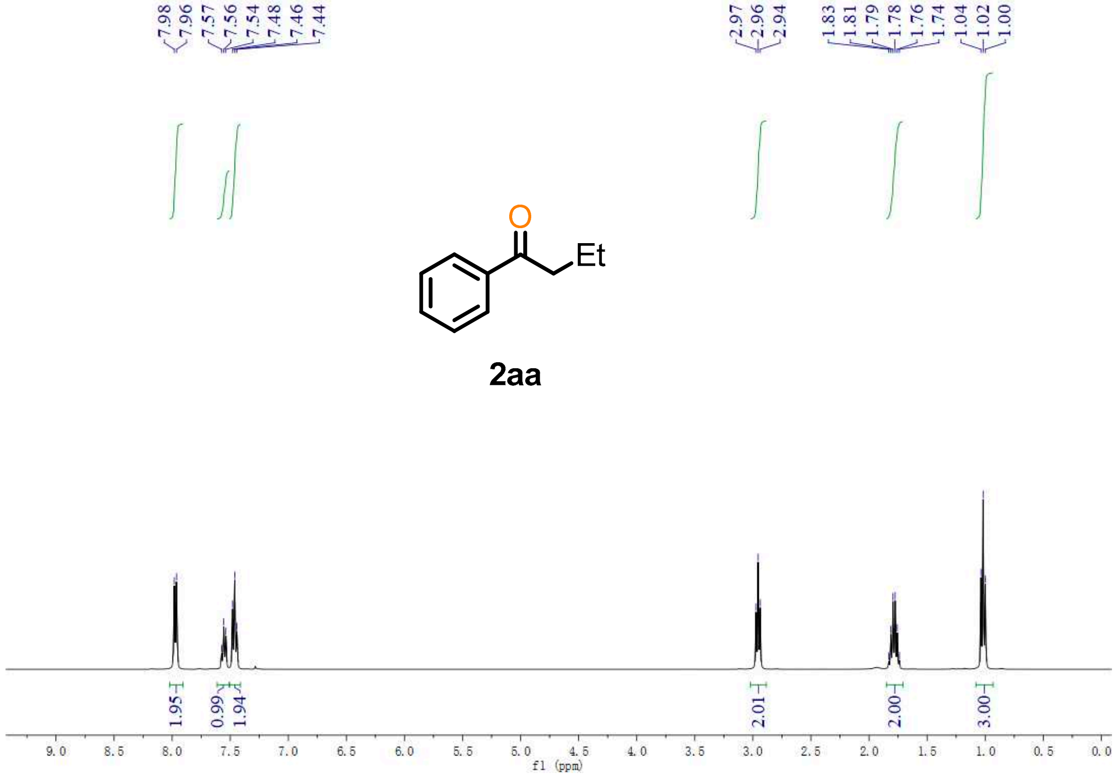

^{13}C NMR spectrum of 1-phenylbutan-1-one (2aa, 100 MHz, $CDCl_3$)

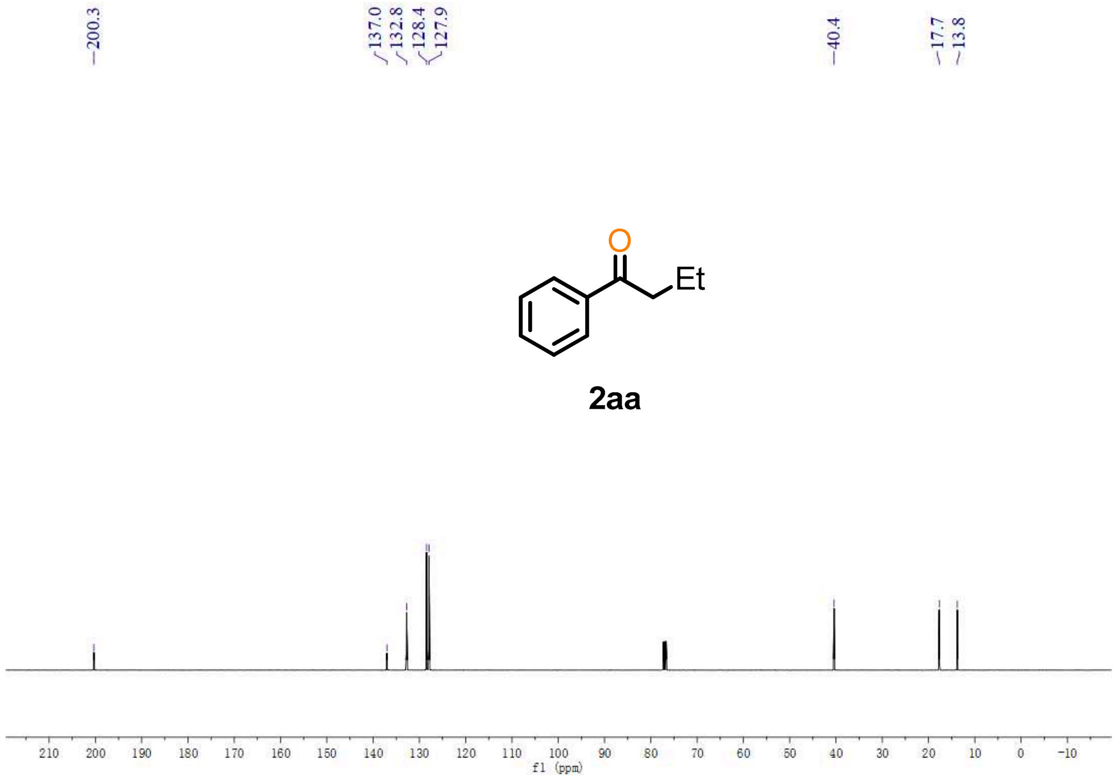

^{1}H NMR spectrum of 1-phenyloctan-1-one (2ab, 400 MHz, $CDCl_3$)

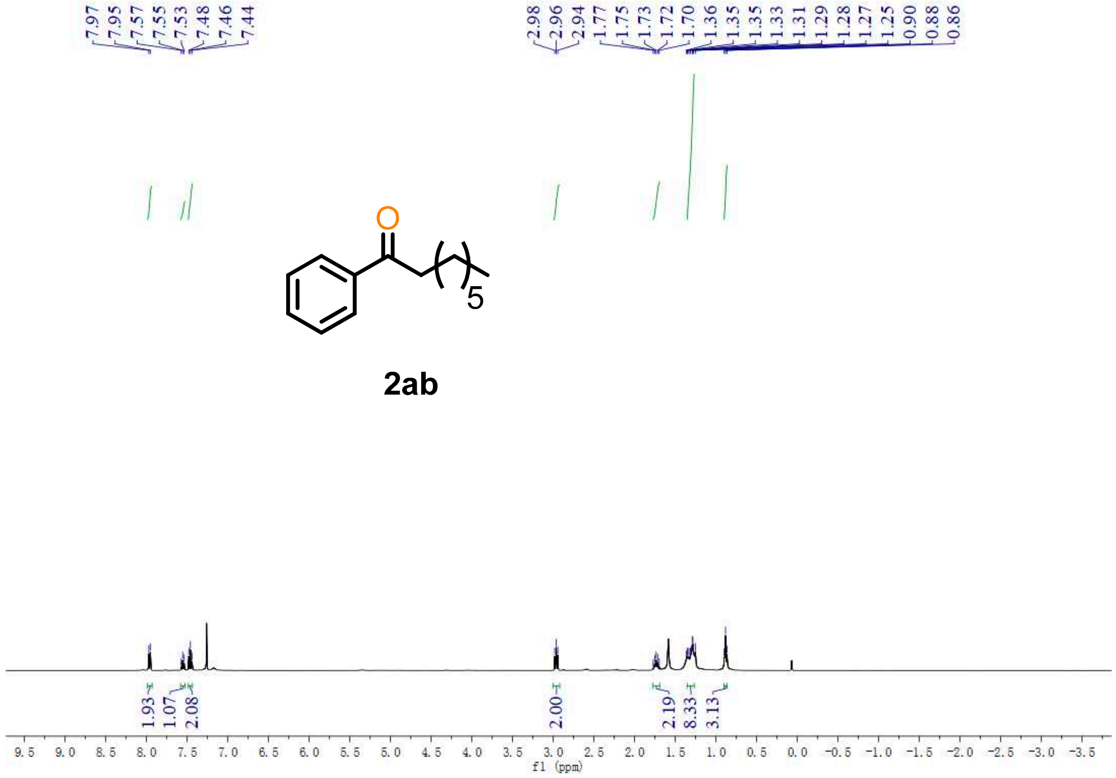

^{13}C NMR spectrum of 1-phenyloctan-1-one (2ab, 100 MHz, $CDCl_3$)

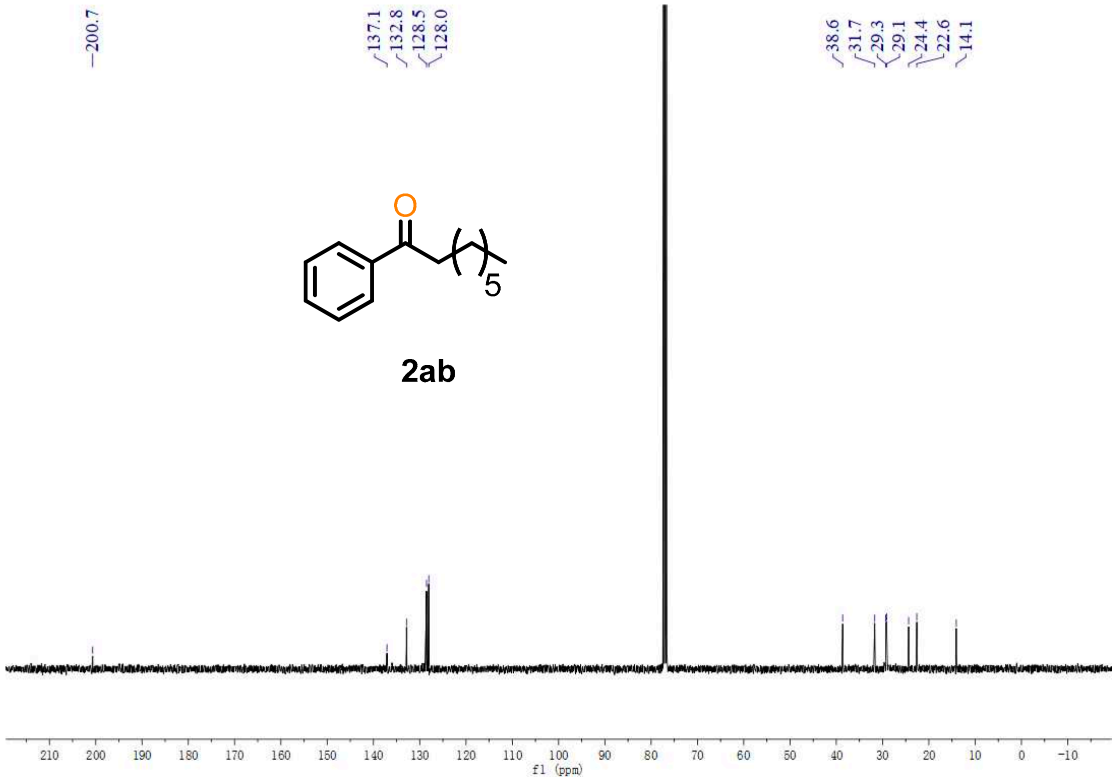

^{1}H NMR spectrum of benzophenone (2ac, 400 MHz, $CDCl_3$)

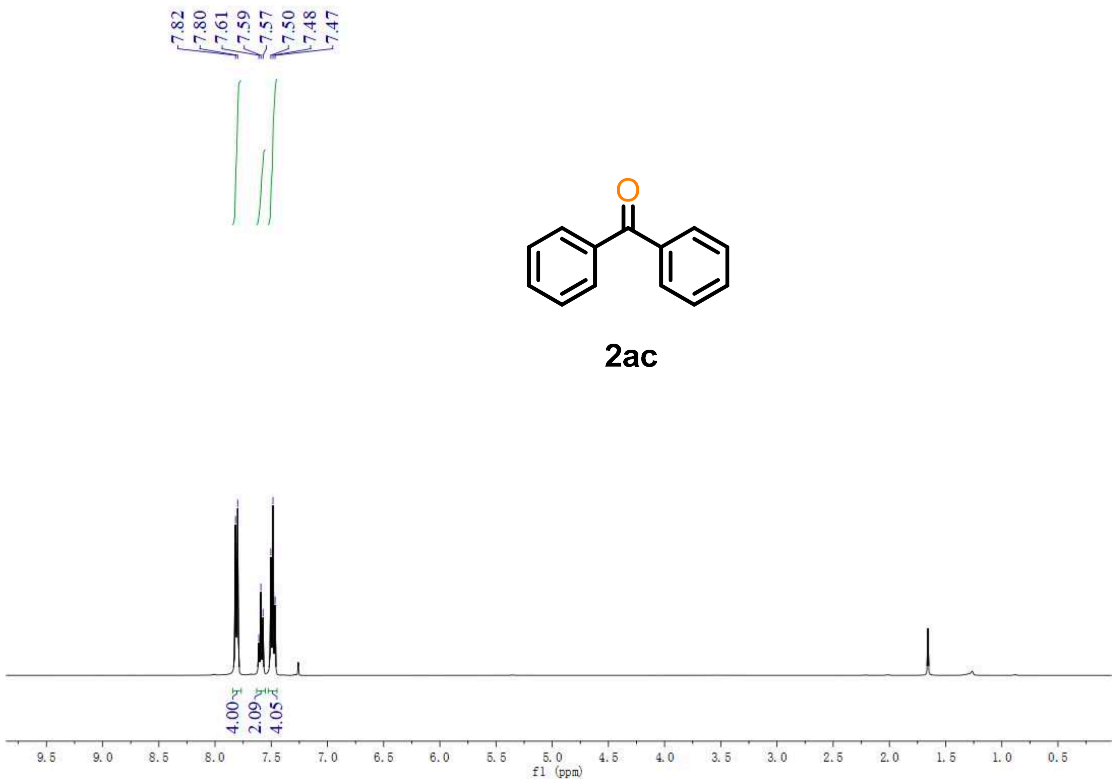

^{13}C NMR spectrum of benzophenone (2ac, 100 MHz, $CDCl_3$)

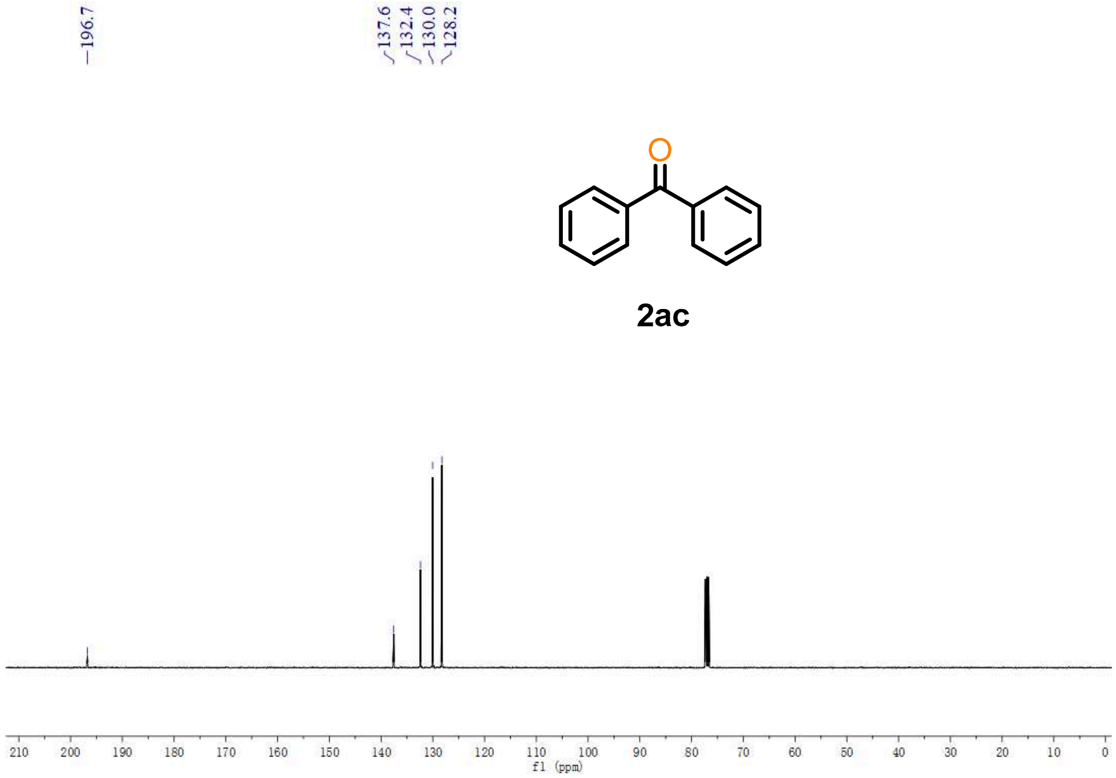

^{1}H NMR spectrum of phenyl(*p*-tolyl)methanone (2ad, 400 MHz, $CDCl_3$)

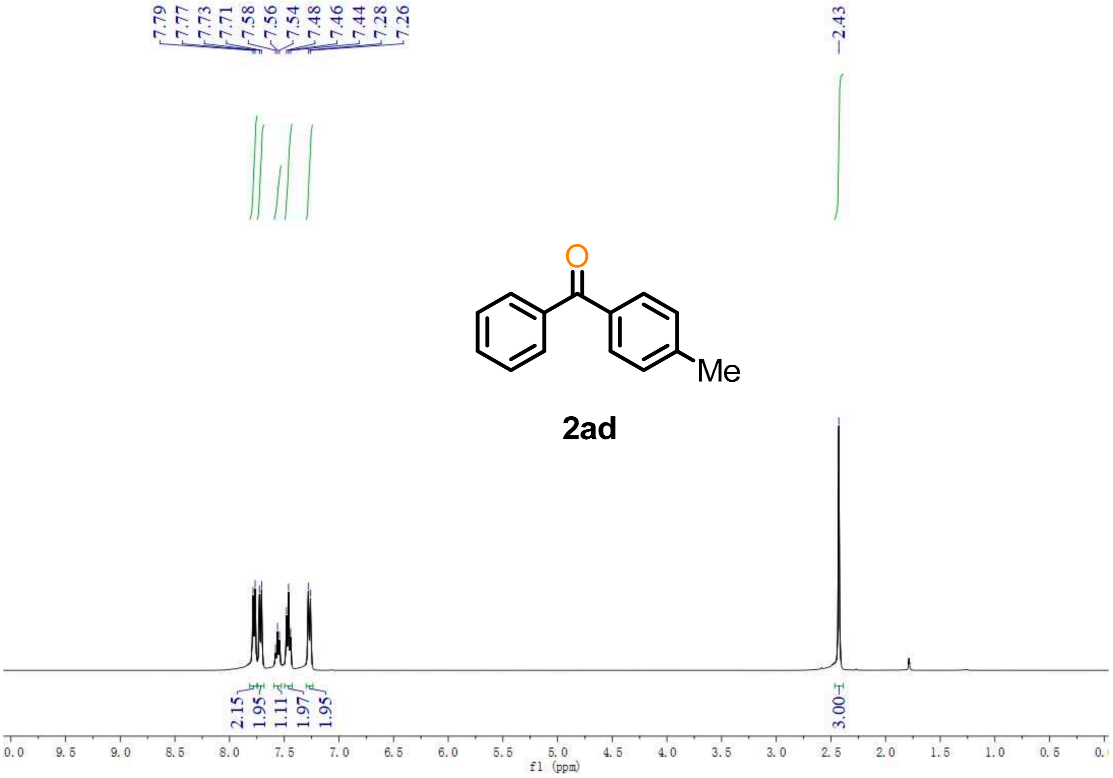

^{13}C NMR spectrum of phenyl(*p*-tolyl)methanone (2ad, 100 MHz, $CDCl_3$)

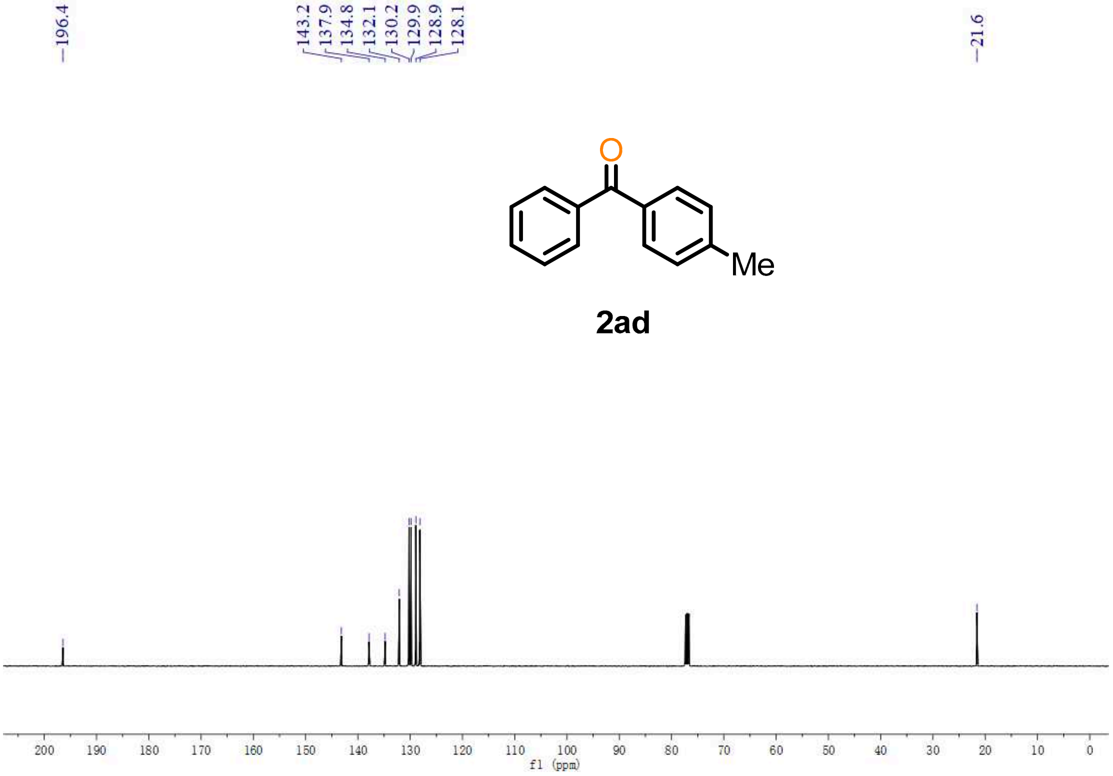

^{1}H NMR spectrum of (4-methoxyphenyl)(phenyl)methanone (2ae, 400 MHz, $CDCl_3$)

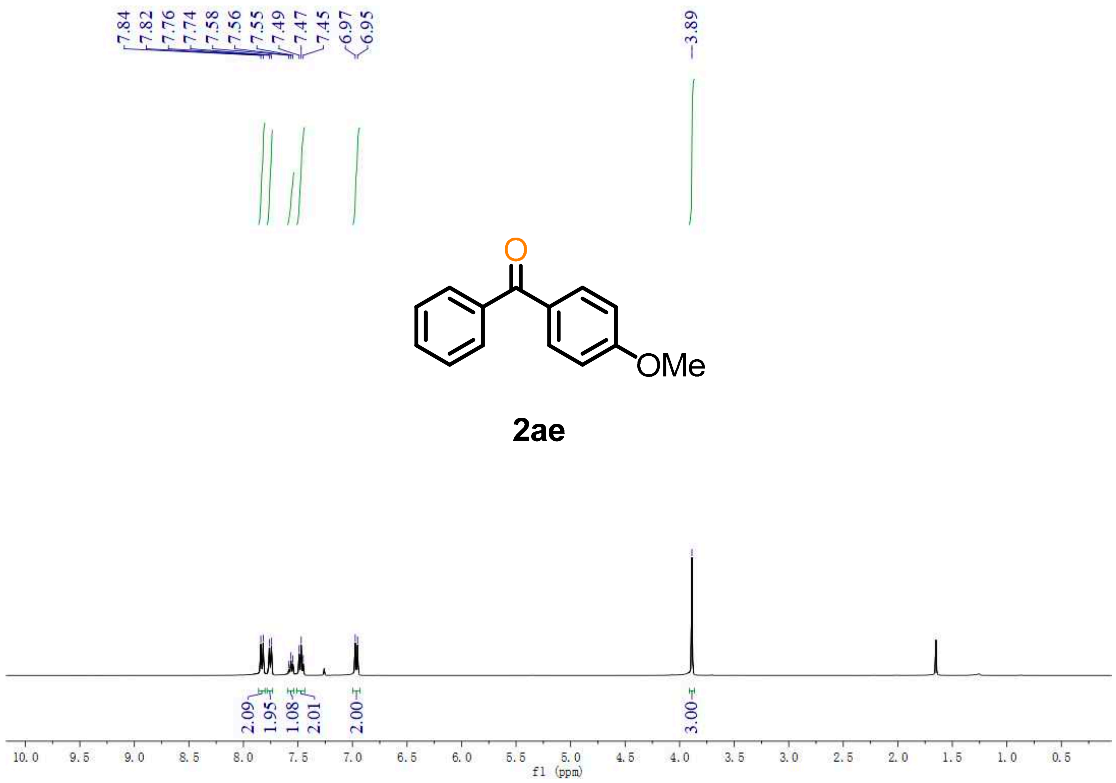

^{13}C NMR spectrum of (4-methoxyphenyl)(phenyl)methanone (2ae, 100 MHz, $CDCl_3$)

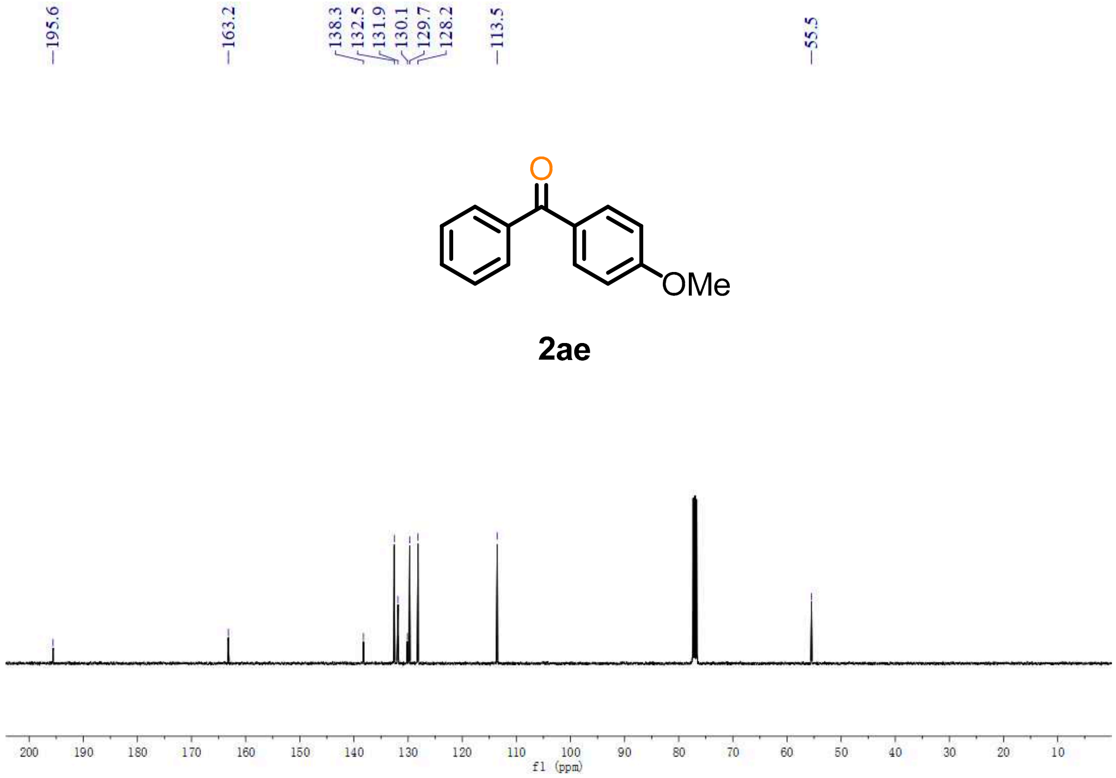

^{1}H NMR spectrum of (4-chlorophenyl)(phenyl)methanone (2af, 400 MHz, $CDCl_3$)

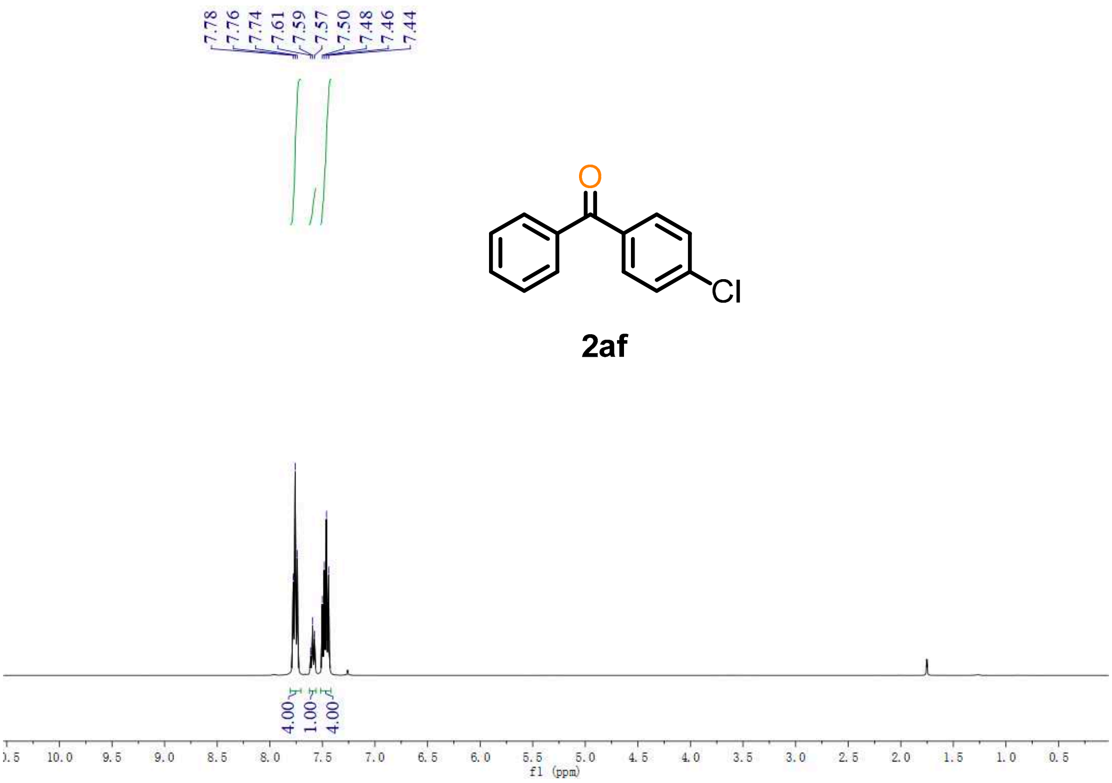

^{13}C NMR spectrum of (4-chlorophenyl)(phenyl)methanone (2af, 100 MHz, $CDCl_3$)

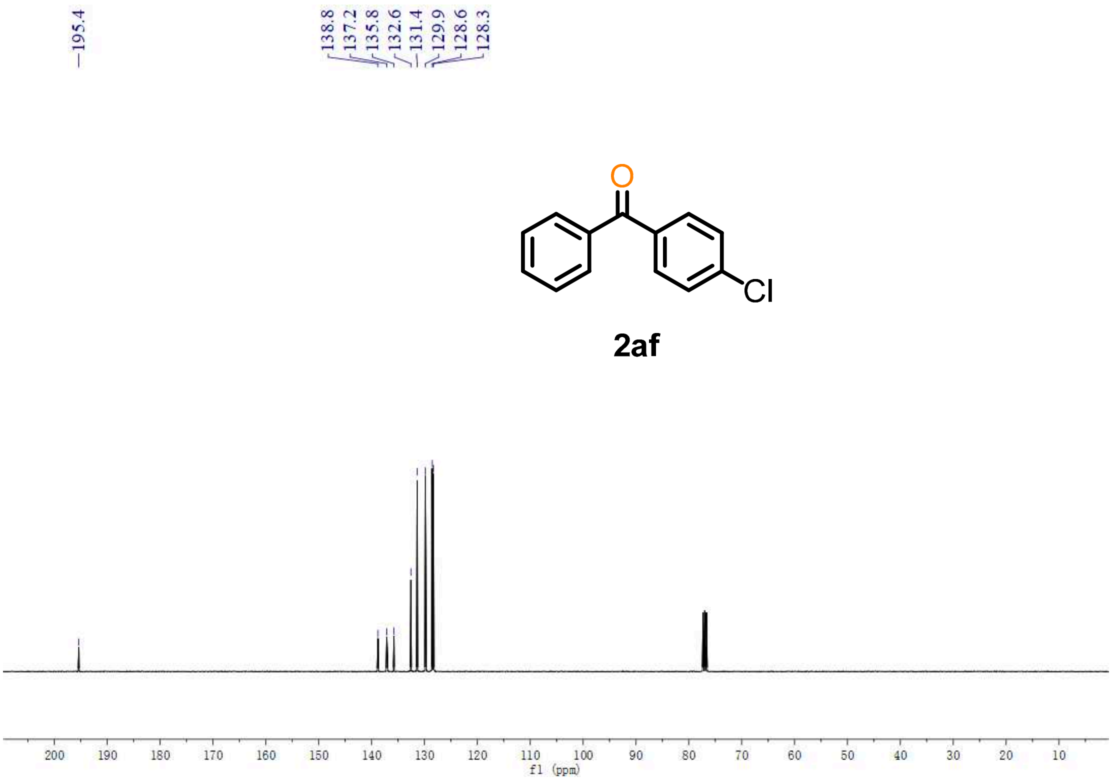

^{1}H NMR spectrum of **anthracene-9,10-dione** (2ag, 400 MHz, $CDCl_3$)

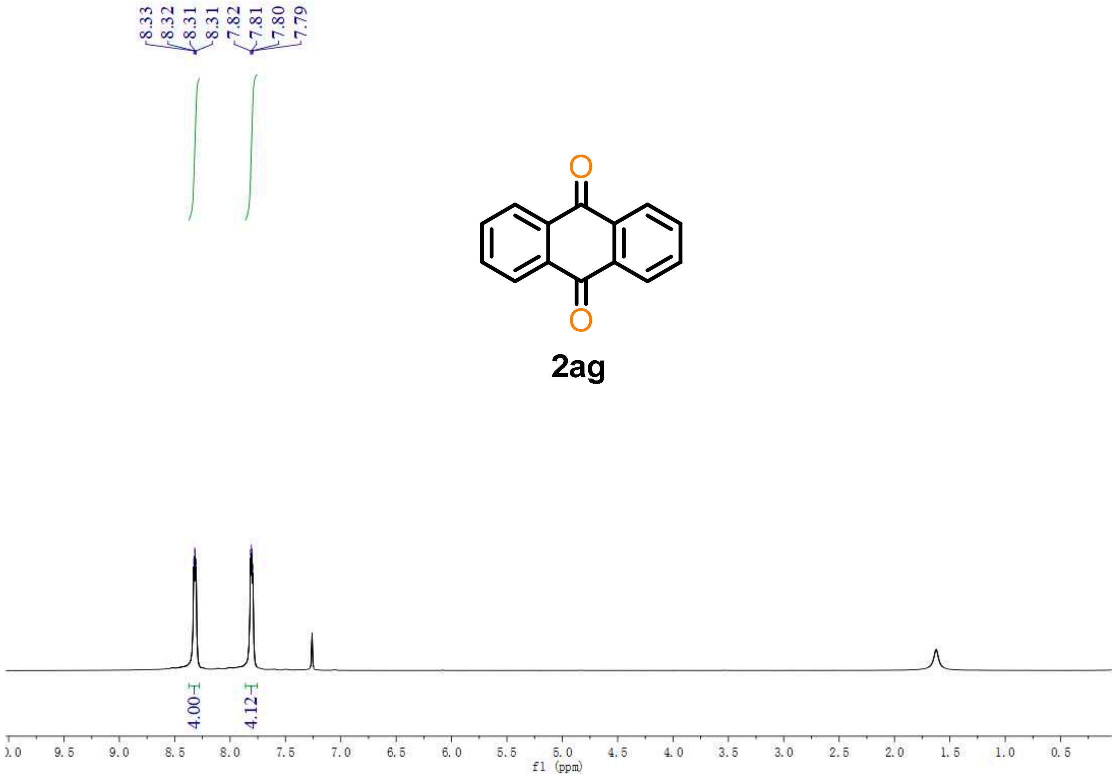

^{13}C NMR spectrum of **anthracene-9,10-dione** (2ag, 100 MHz, $CDCl_3$)

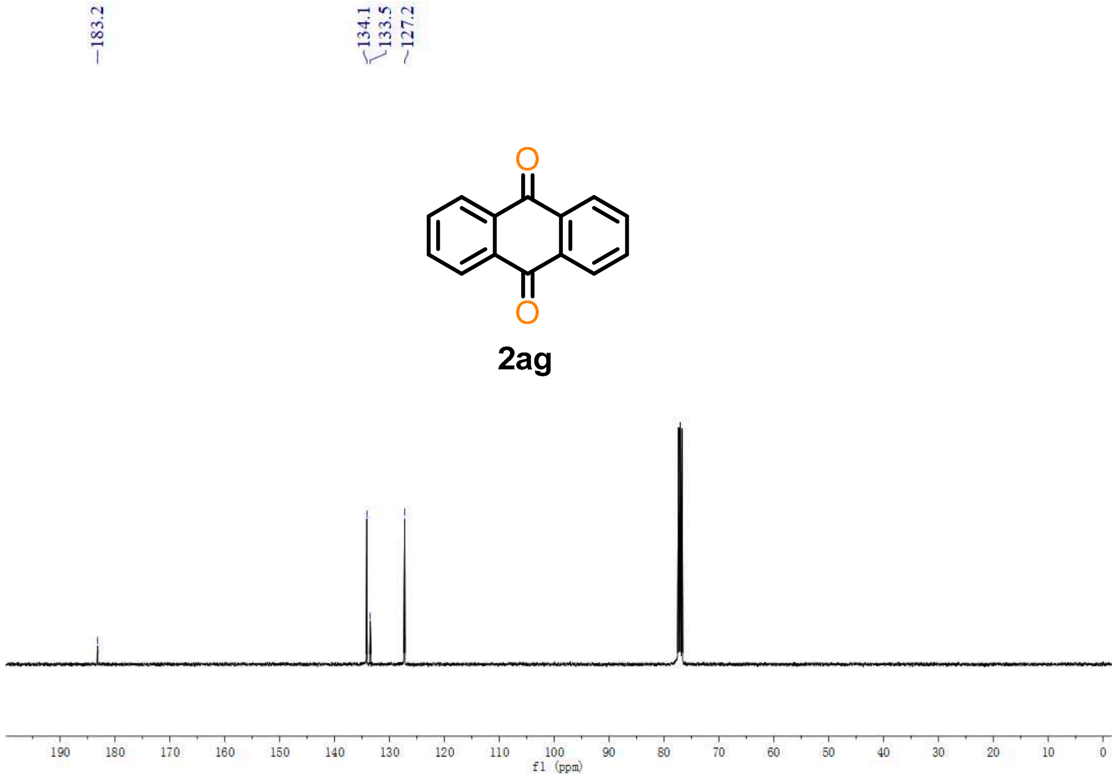

^{1}H NMR spectrum of benzoic acid (2ah, 400 MHz, DMSO-d6)

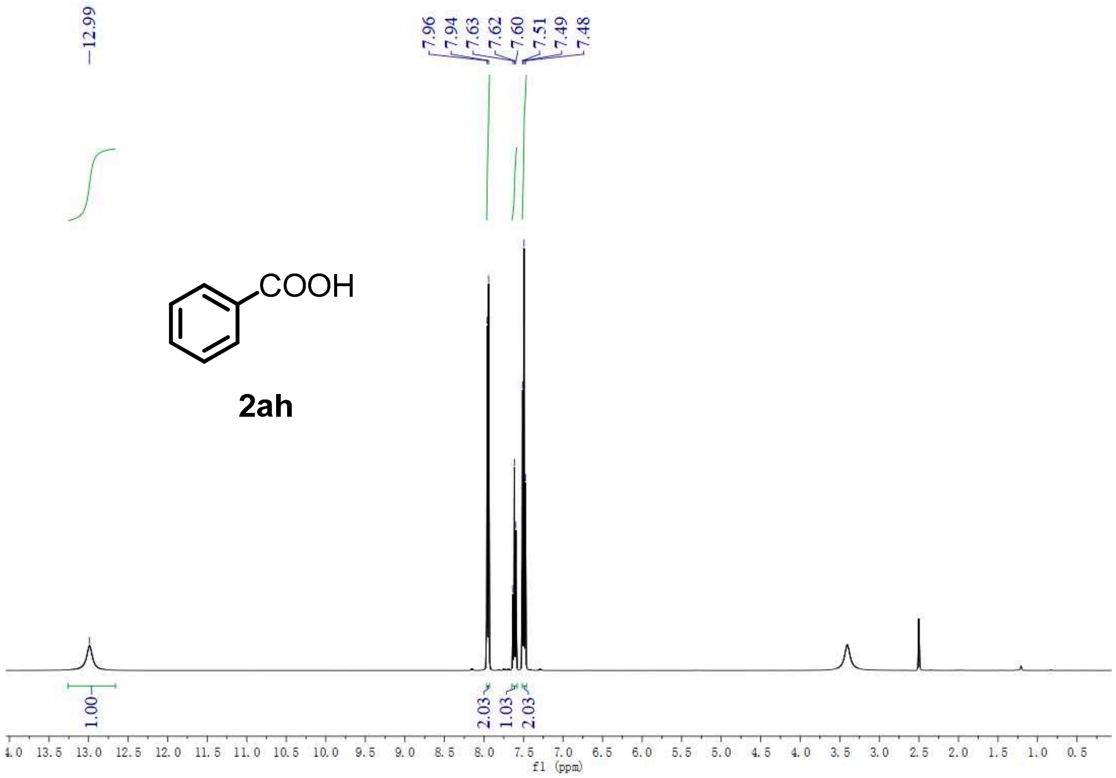

^{13}C NMR spectrum of benzoic acid (2ah, 100 MHz, DMSO-d6)

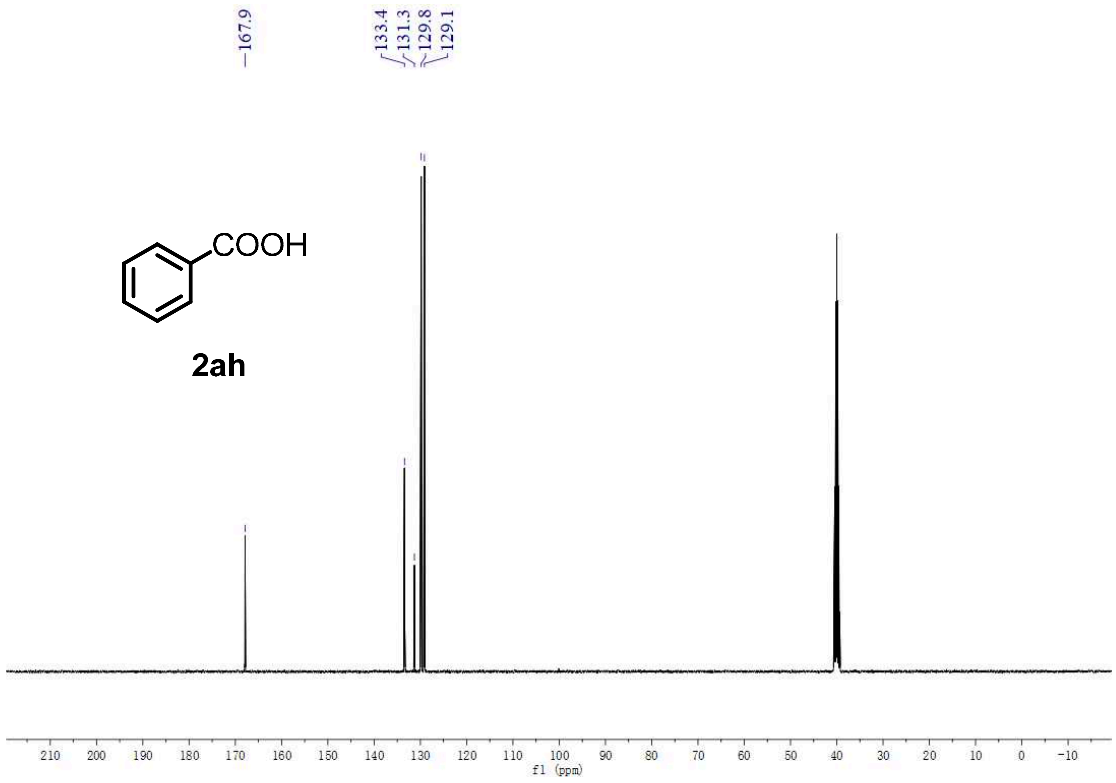

^{1}H NMR spectrum of methyl 2-(1-(4-acetylbenzoyl)-5-methoxy-2-methyl-1*H*-indol-3-yl)acetate (2ai, 400 MHz, $CDCl_3$)

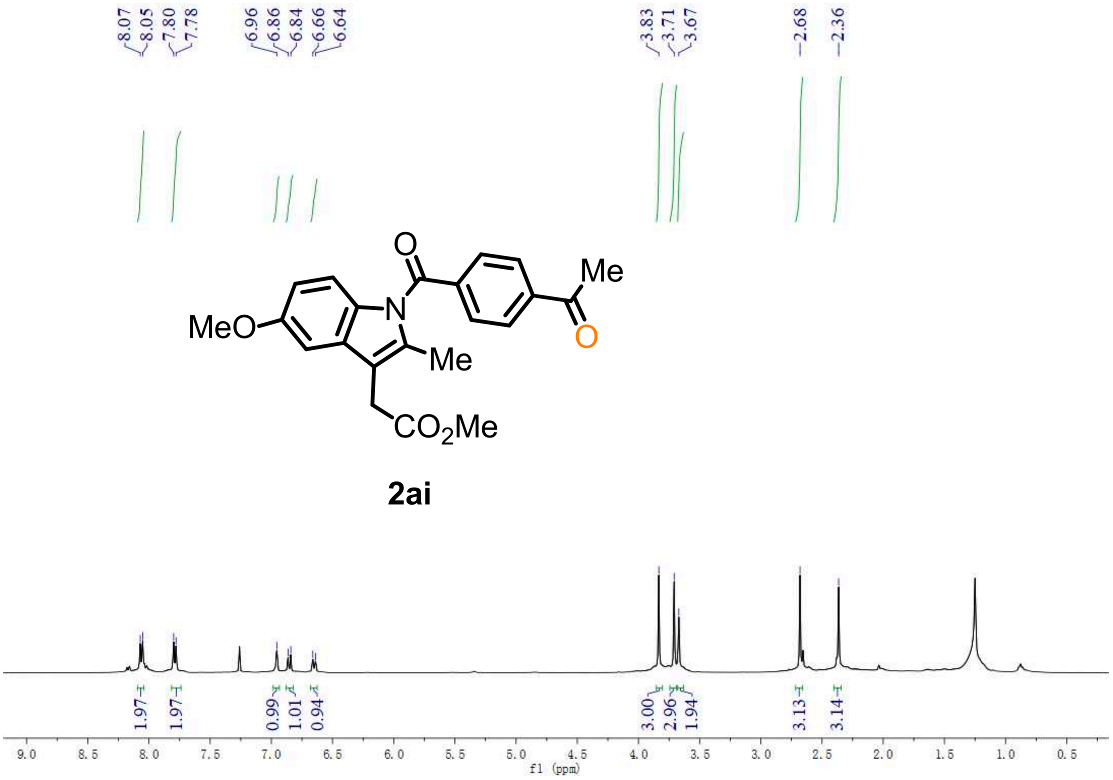

^{13}C NMR spectrum of methyl 2-(1-(4-acetylbenzoyl)-5-methoxy-2-methyl-1*H*-indol-3-yl)acetate (2ai, 100 MHz, $CDCl_3$)

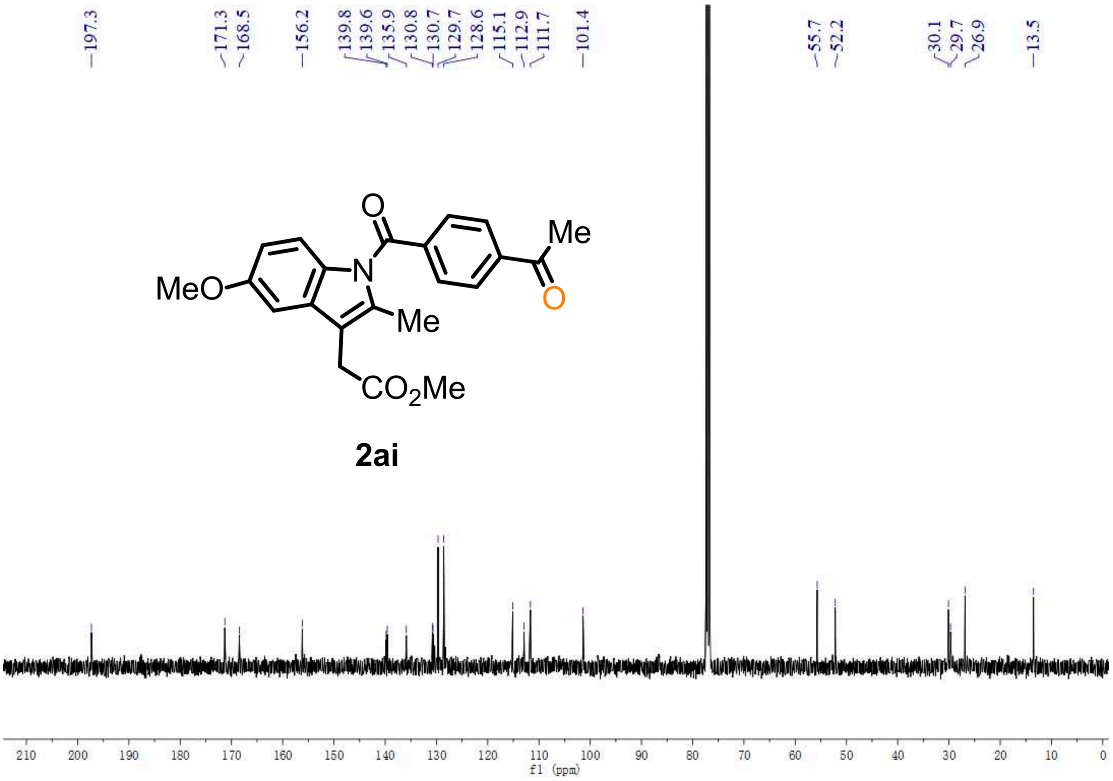

^{1}H NMR spectrum of (3S,8S,9S,10R,13R,14S,17R)-10,13-dimethyl-17-((R)-6-methylheptan-2-yl)-2,3,4,7,8,9,10,11,12,13,14,15,16,17-tetradecahydro-1*H*-cyclopenta[*a*]phenanthren-3-yl 4-acetylbenzoate (2aj, 400 MHz, $CDCl_3$)

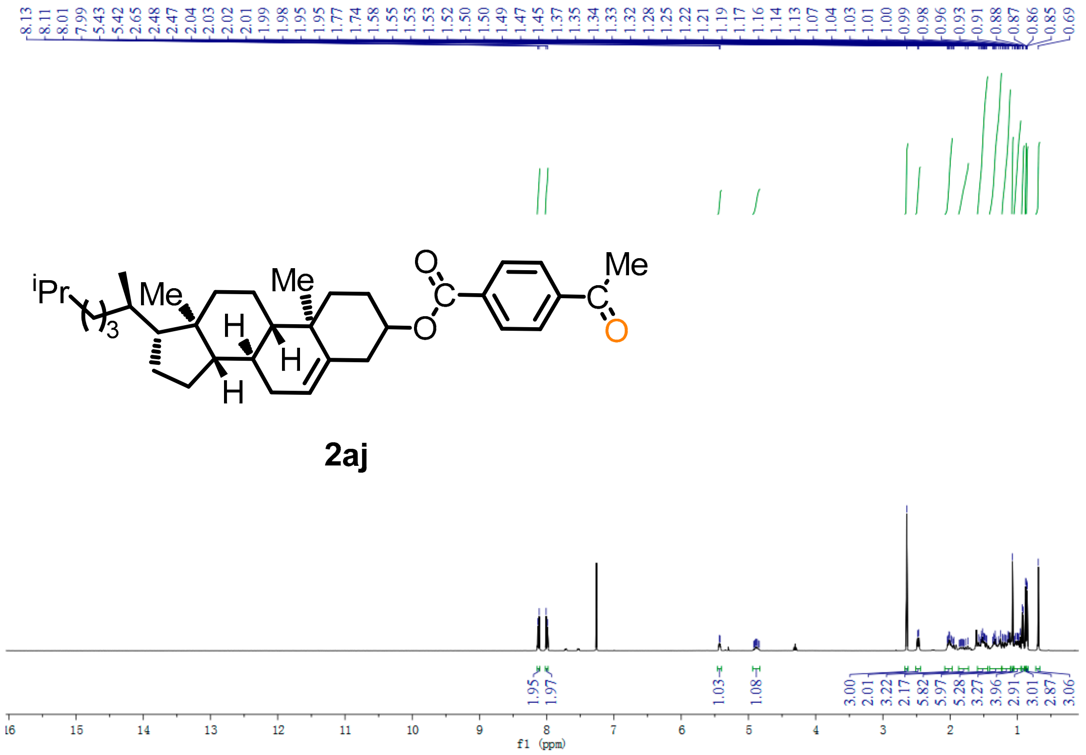

^{13}C NMR spectrum of (3S,8S,9S,10R,13R,14S,17R)-10,13-dimethyl-17-((R)-6-methylheptan-2-yl)-2,3,4,7,8,9,10,11,12,13,14,15,16,17-tetradecahydro-1*H*-cyclopenta[*a*]phenanthren-3-yl 4-acetylbenzoate (2aj, 100 MHz, $CDCl_3$)

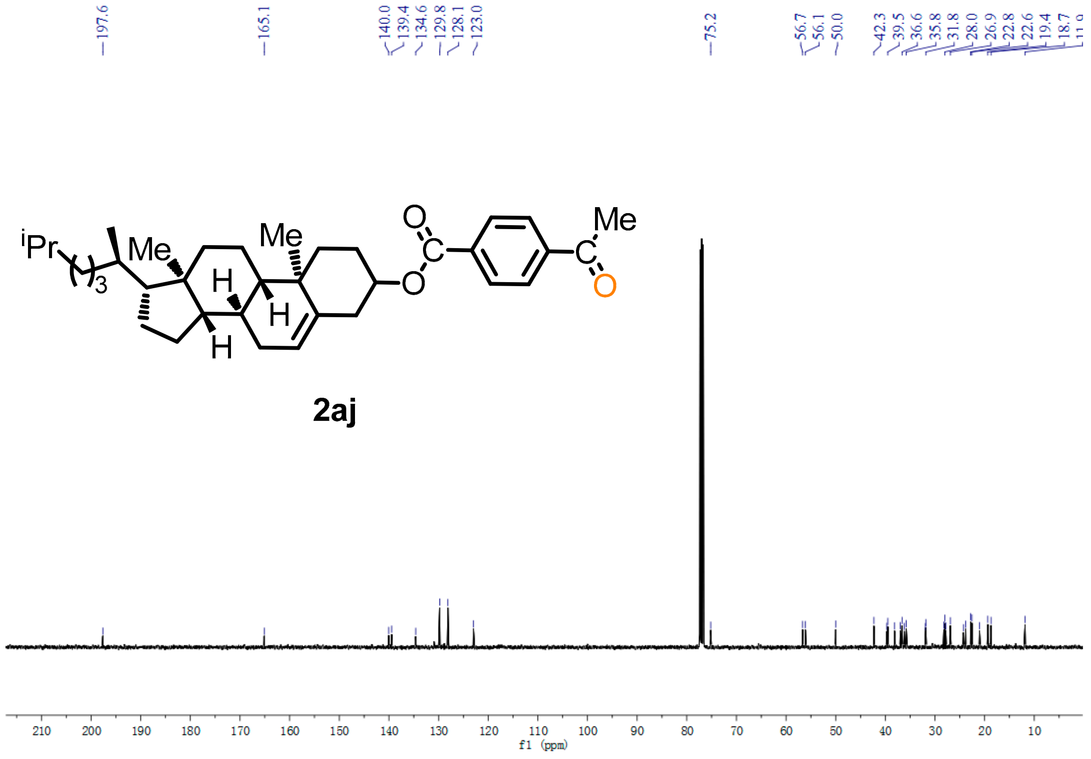

^{1}H NMR spectrum of (3S,8R,9S,10R,13S,14S)-10,13-dimethyl-17-oxo-2,3,4,7,8,9,10,11,12,13,14,15,16,17-tetradecahydro-1*H*-cyclopenta[*a*]phenanthren-3-yl 4-acetylbenzoate (2ak, 400 MHz, $CDCl_3$)

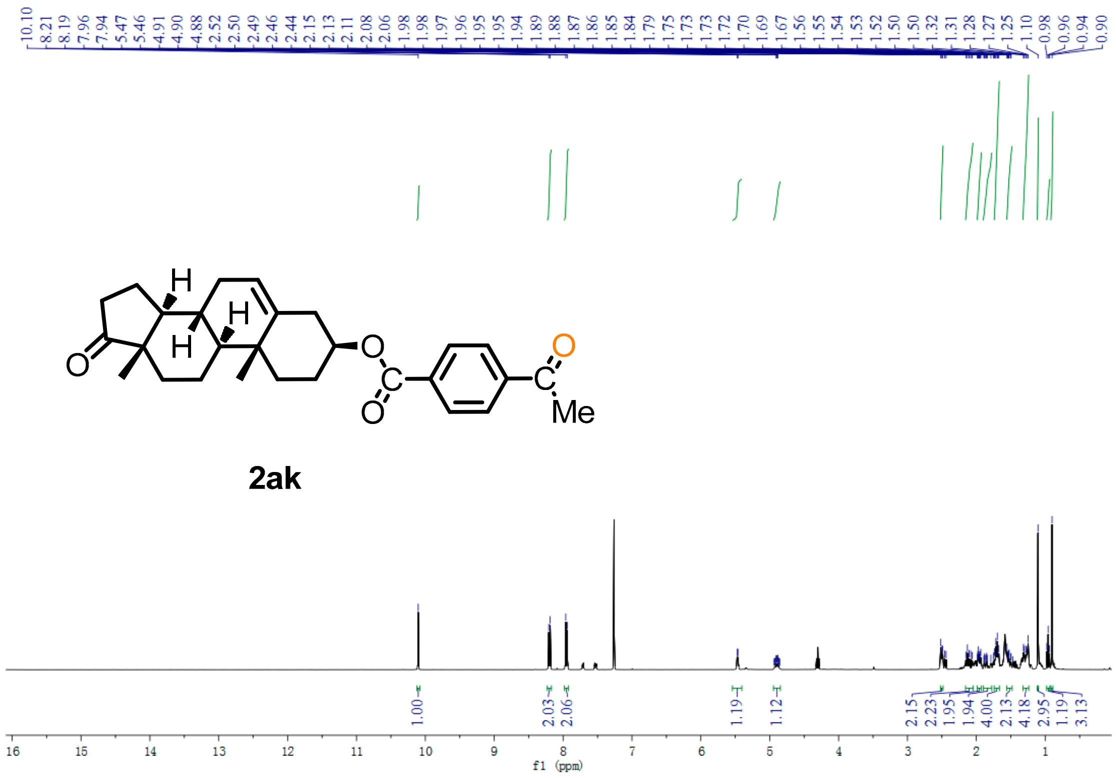

^{13}C NMR spectrum of (3S,8R,9S,10R,13S,14S)-10,13-dimethyl-17-oxo-2,3,4,7,8,9,10,11,12,13,14,15,16,17-tetradecahydro-1*H*-cyclopenta[*a*]phenanthren-3-yl 4-acetylbenzoate (2ak, 100 MHz, $CDCl_3$)

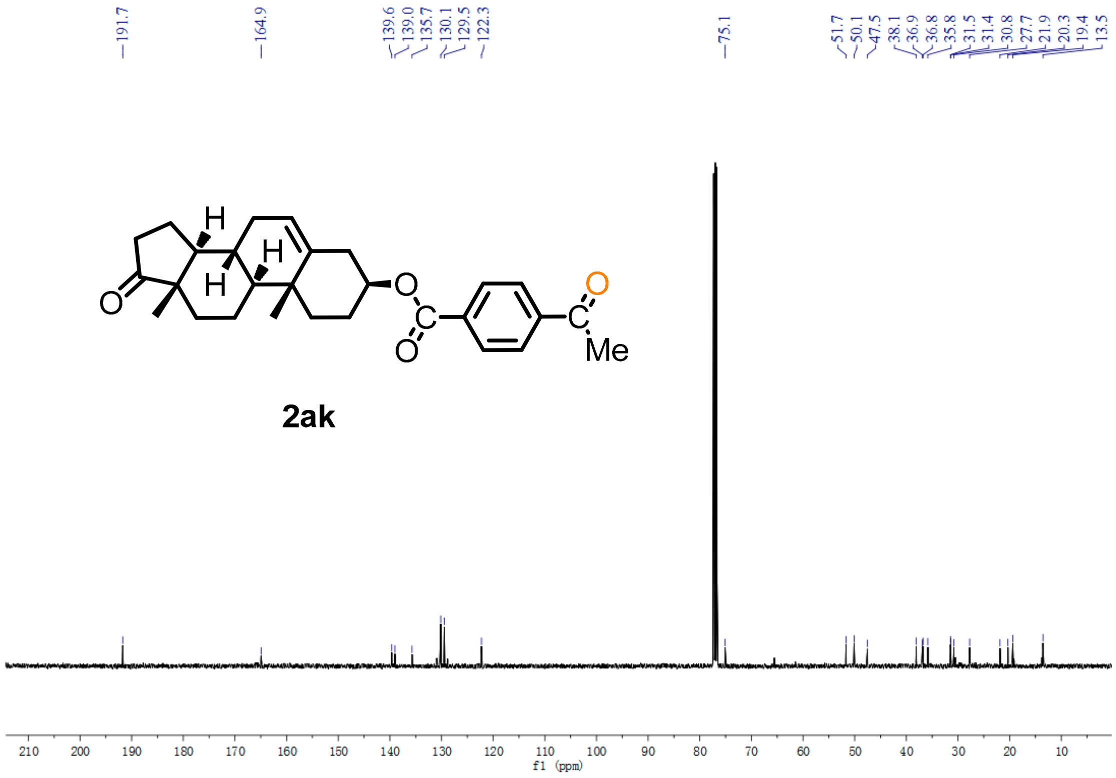

^{1}H NMR spectrum of isopropyl 2-(4-(4-acetylbenzoyl)phenoxy)-2-methylpropanoate (2al, 400 MHz, $CDCl_3$)

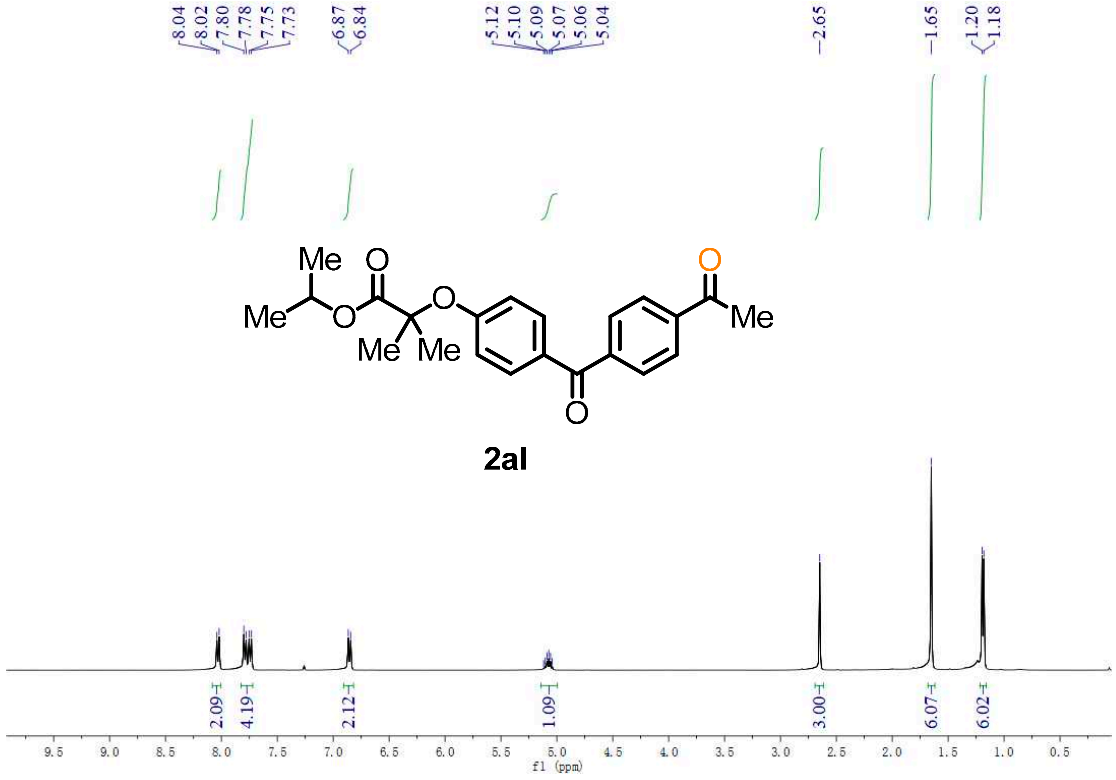

^{13}C NMR spectrum of isopropyl 2-(4-(4-acetylbenzoyl)phenoxy)-2-methylpropanoate (2al, 100 MHz, $CDCl_3$)

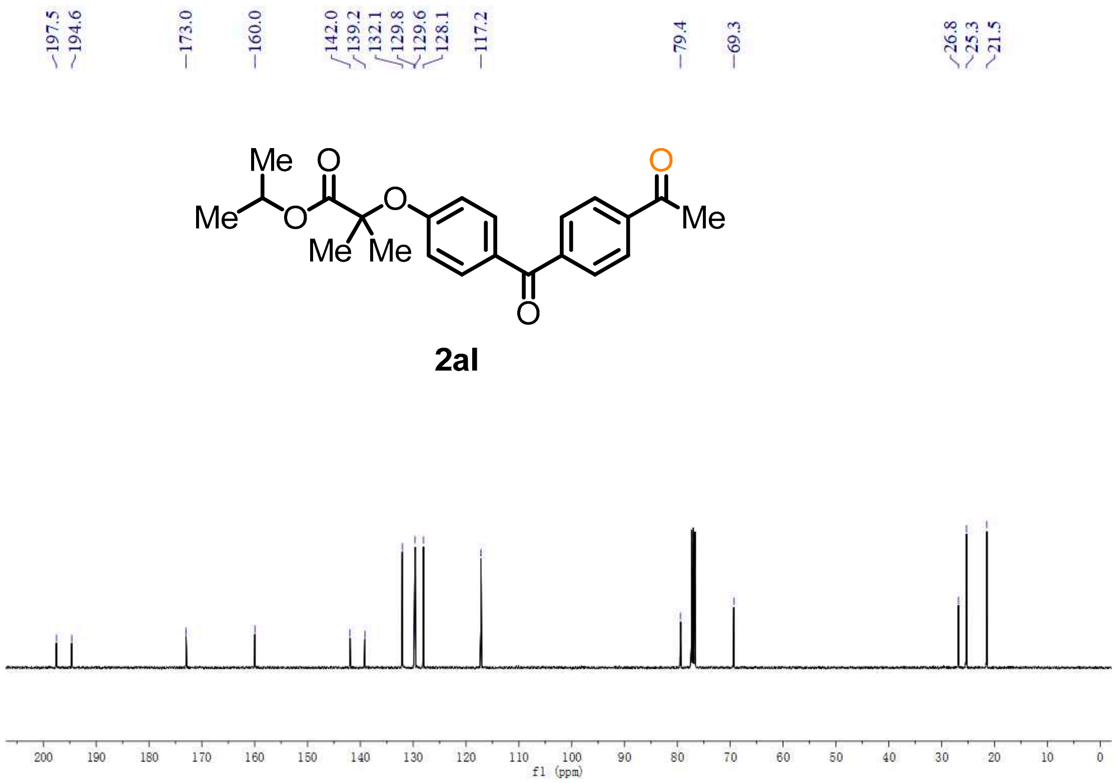

^{1}H NMR spectrum of anthracene (4a, 400 MHz, $CDCl_3$)

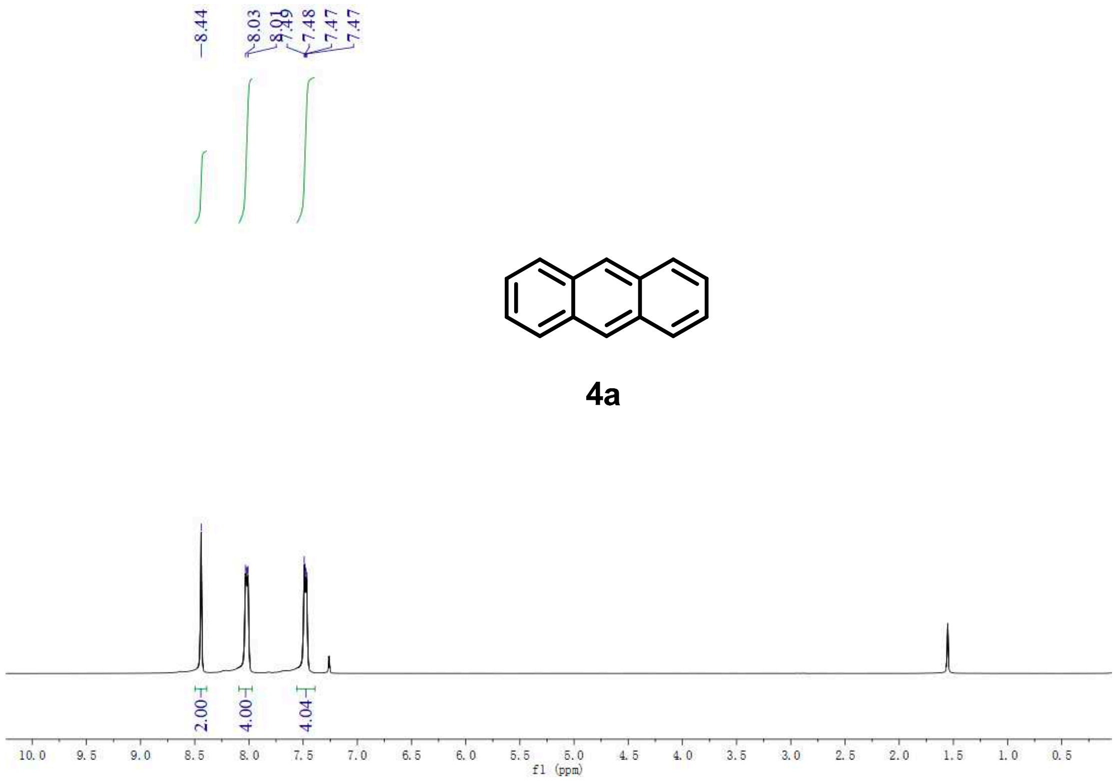

^{13}C NMR spectrum of anthracene (4a, 100 MHz, $CDCl_3$)

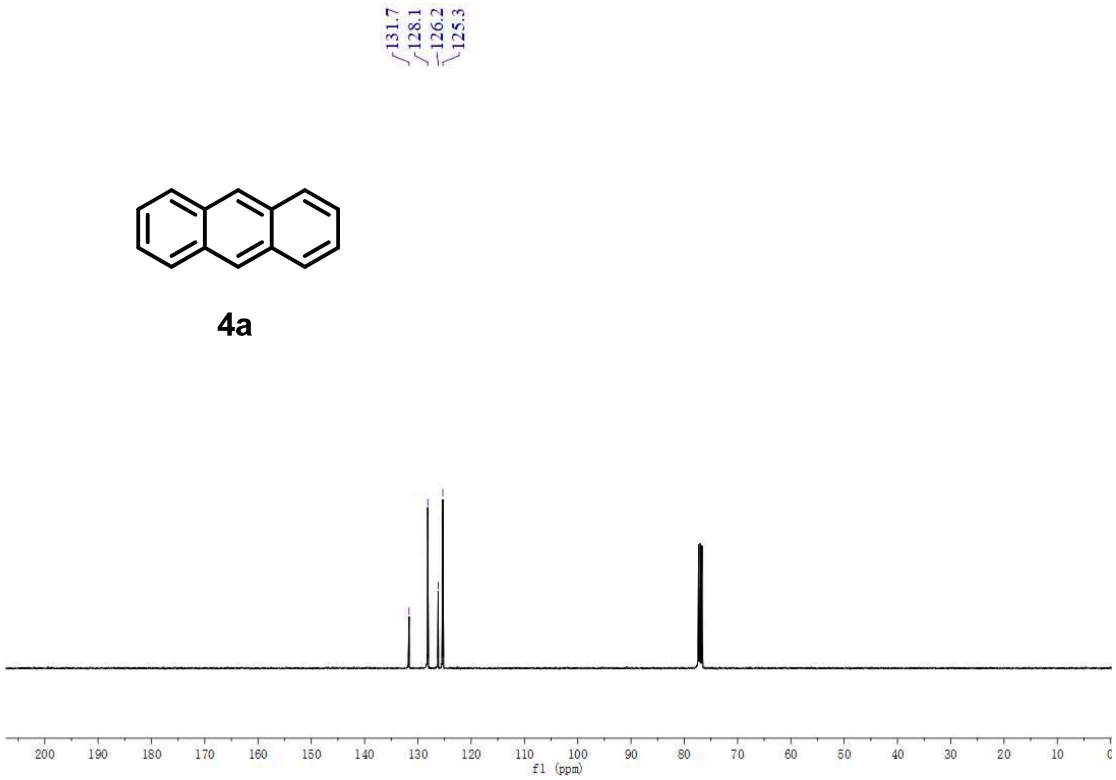

^{1}H NMR spectrum of chalcone (4b, 400 MHz, $CDCl_3$)

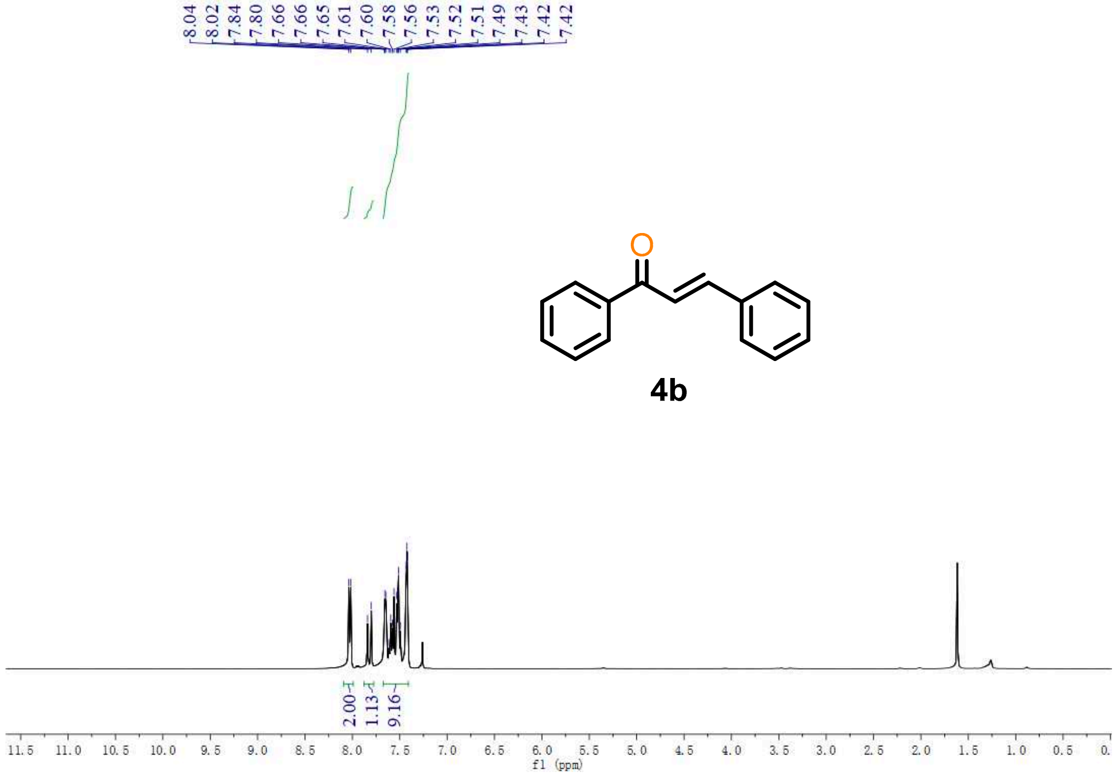

^{13}C NMR spectrum of chalcone (4b, 100 MHz, $CDCl_3$)

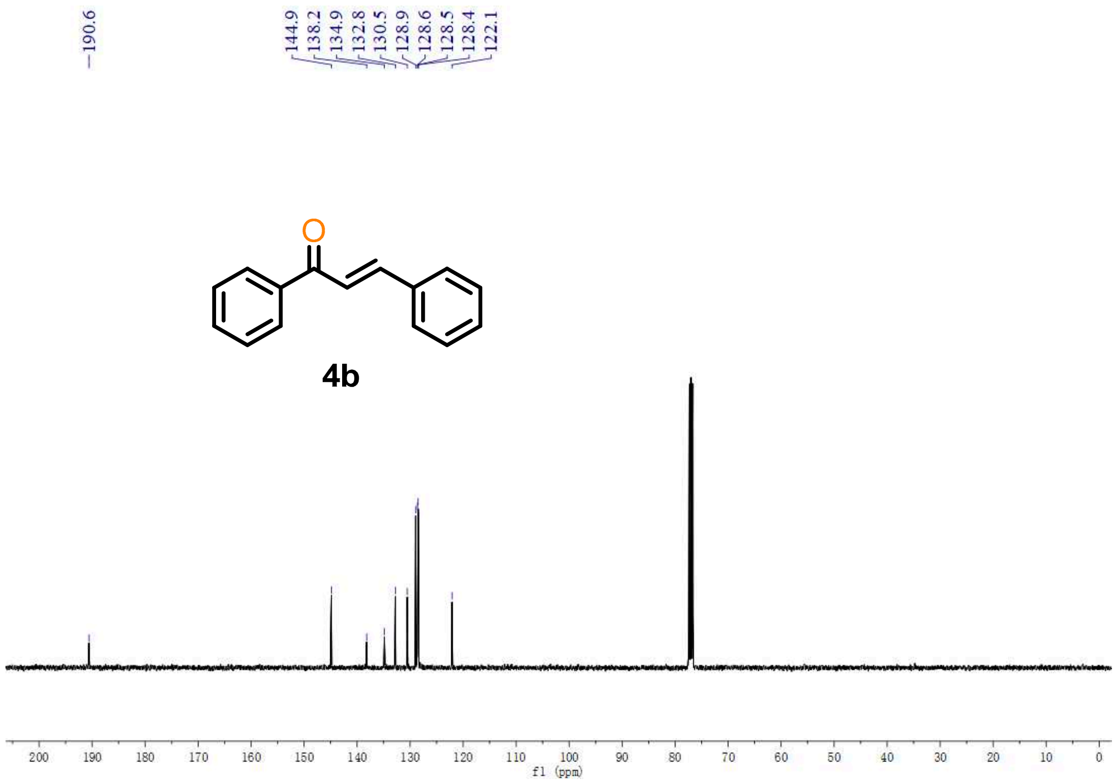

¹H NMR spectrum of 2-phenyl-1*H*-benzo[*d*]imidazole (4c, 400 MHz, DMSO-*d6*)

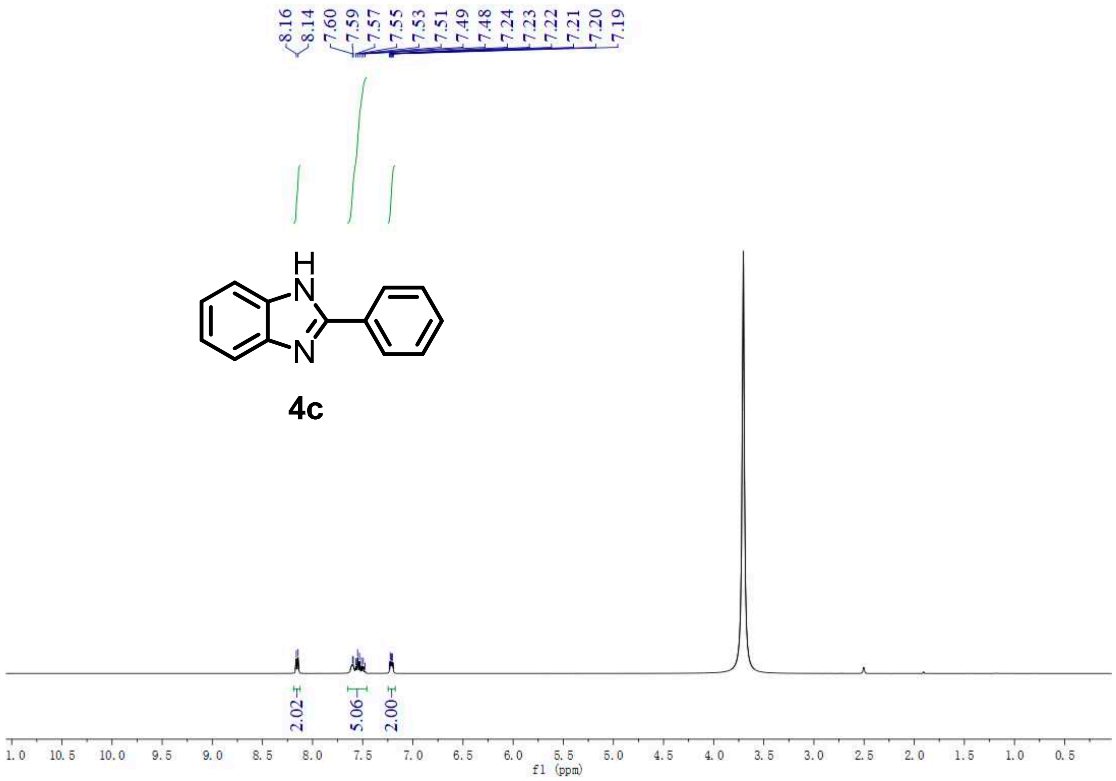

^{13}C NMR spectrum of 2-phenyl-1*H*-benzo[*d*]imidazole (4c, 100 MHz, DMSO-*d6*)

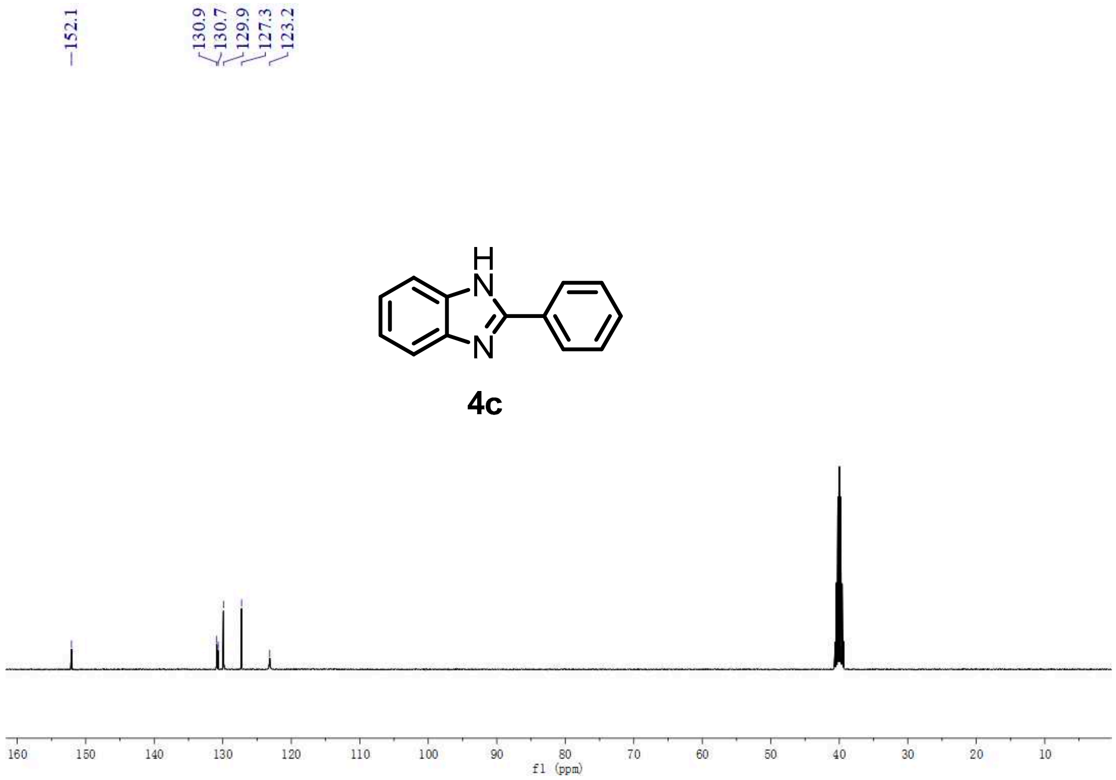

^{1}H NMR spectrum of *N*-phenylacetamide (4d, 400 MHz, $CDCl_3$)

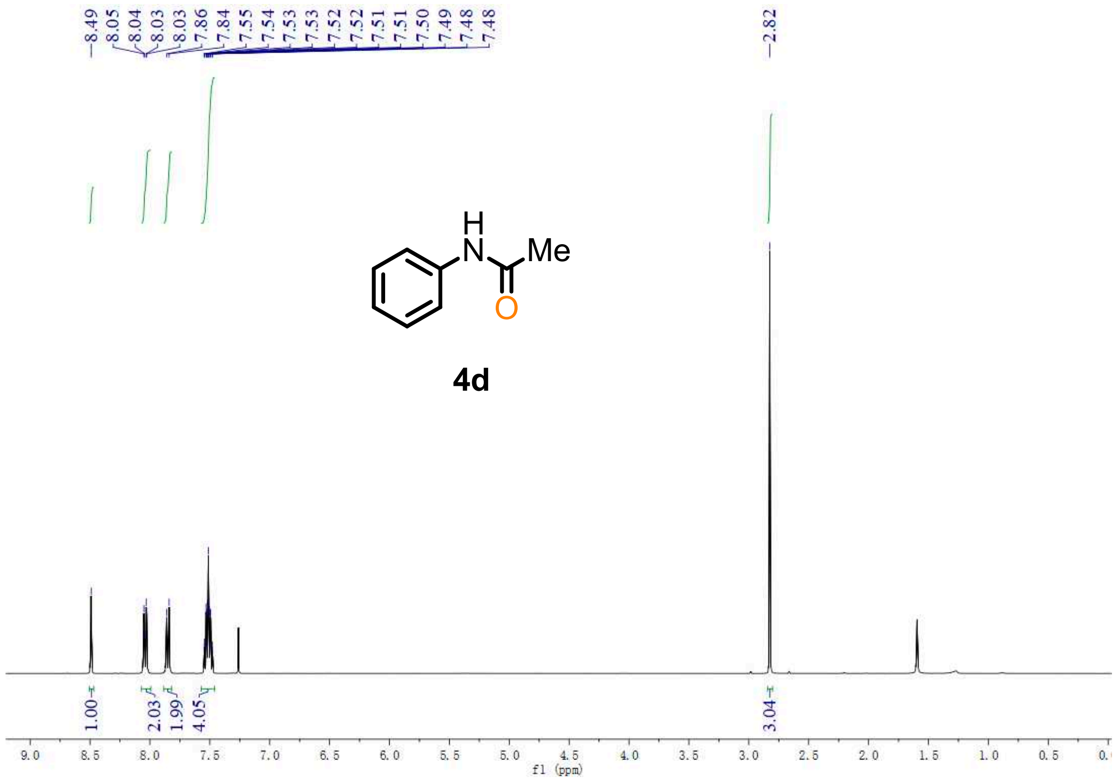

^{13}C NMR spectrum of *N*-phenylacetamide (4d, 100 MHz, $CDCl_3$)

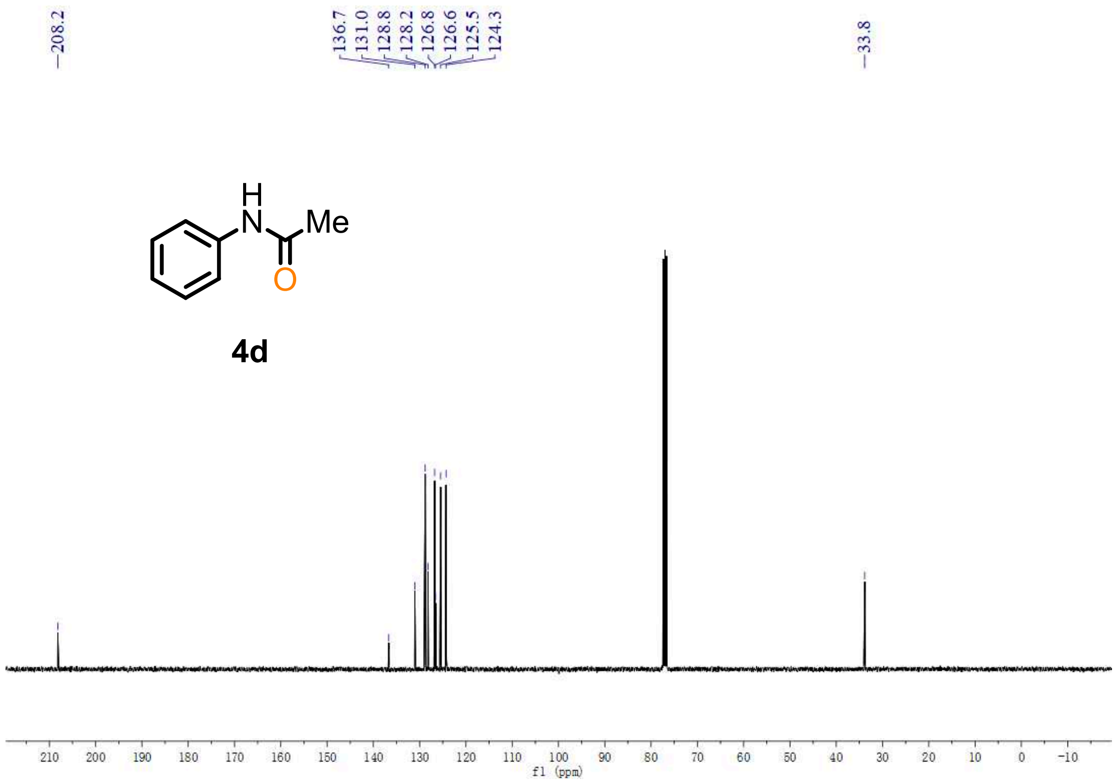

208.2
136.7
131.0
128.8
128.2
126.8
126.6
125.5
124.3
33.8
Me
4d
210
200
190
180
170
160
150
140
130
120
110
100
90
80
70
60
50
40
30
20
10
0
-10
f1 (ppm)
